# Inference of Population Structure using Dense Haplotype Data

**DOI:** 10.1371/journal.pgen.1002453

**Published:** 2012-01-26

**Authors:** Daniel John Lawson, Garrett Hellenthal, Simon Myers, Daniel Falush

**Affiliations:** 1 Department of Mathematics, University of Bristol, Bristol, United Kingdom; 2 Wellcome Trust Center for Human Genetics, Oxford, United Kingdom; 3 Department of Statistics, University of Oxford, Oxford, United Kingdom; 4 Environmental Research Institute, University College Cork, Cork, Ireland; 5 Max Planck Institute for Evolutionary Anthropology, Leipzig, Germany; The University of North Carolina at Chapel Hill, United States of America

## Abstract

The advent of genome-wide dense variation data provides an opportunity to investigate ancestry in unprecedented detail, but presents new statistical challenges. We propose a novel inference framework that aims to efficiently capture information on population structure provided by patterns of haplotype similarity. Each individual in a sample is considered in turn as a recipient, whose chromosomes are reconstructed using chunks of DNA donated by the other individuals. Results of this “chromosome painting” can be summarized as a “coancestry matrix,” which directly reveals key information about ancestral relationships among individuals. If markers are viewed as independent, we show that this matrix almost completely captures the information used by both standard Principal Components Analysis (PCA) and model-based approaches such as STRUCTURE in a unified manner. Furthermore, when markers are in linkage disequilibrium, the matrix combines information across successive markers to increase the ability to discern fine-scale population structure using PCA. In parallel, we have developed an efficient model-based approach to identify discrete populations using this matrix, which offers advantages over PCA in terms of interpretability and over existing clustering algorithms in terms of speed, number of separable populations, and sensitivity to subtle population structure. We analyse Human Genome Diversity Panel data for 938 individuals and 641,000 markers, and we identify 226 populations reflecting differences on continental, regional, local, and family scales. We present multiple lines of evidence that, while many methods capture similar information among strongly differentiated groups, more subtle population structure in human populations is consistently present at a much finer level than currently available geographic labels and is only captured by the haplotype-based approach. The software used for this article, ChromoPainter and fineSTRUCTURE, is available from http://www.paintmychromosomes.com/.

## Author Summary

The first step in almost every genetic analysis is to establish how sample members are related to each other. High relatedness between individuals can arise if they share a small number of recent ancestors, e.g. if they are distant cousins or a larger number of more distant ones, e.g. if their ancestors come from the same region. The most popular methods for investigating these relationships analyse successive markers independently, simply adding the information they provide. This works well for studies involving hundreds of markers scattered around the genome but is less appropriate now that entire genomes can be sequenced. We describe a “chromosome painting” approach to characterising shared ancestry that takes into account the fact that DNA is transmitted from generation to generation as a linear molecule in chromosomes. We show that the approach increases resolution relative to previous techniques, allowing differences in ancestry profiles among individuals to be resolved at the finest scales yet. We provide mathematical, statistical, and graphical machinery to exploit this new information and to characterize relationships at continental, regional, local, and family scales.

## Introduction

Technologies such as high density genotyping arrays and next generation resequencing have recently facilitated the production of an enormous quantity of data with which to investigate genetic relationships in humans and in other organisms. These data have the potential to provide a new level of insight into patterns of dispersal and mating, and recent and ancient historical events. However there are challenges, in terms of computational burden and statistical modelling, that are yet to be fully addressed. Two of the most popular approaches to investigate population structure using genetic data are exemplified by principal components analysis (PCA) [1], which is often regarded as a non-parametric approach, and STRUCTURE [2], based on explicitly modelling population structure. It is common to apply both approaches to the same dataset, in order to provide a useful summary of the basic features of the data. The PCA approach is based on analysing a matrix (which can be defined in several different ways, e.g. [3]–[5]) whose entries quantify the genetic similarity between pairs of individuals. The principal components (PCs) of this matrix thus represent directions in sample space that maximally explain the observed pattern of genetic similarity. Visualisation of key patterns of structure in the data can be achieved by plotting successive PCs: clusters of individuals can be interpreted as genetic populations, while admixture of two populations results in sets of individuals lying along a line [6], although other historical events can also produce identical PC signals [4] and other issues can also complicate the interpretation of PCs [4], [7].

Model-based methods attempt to more directly reconstruct historical events. In the simplest version of the STRUCTURE approach [2], individuals are assumed to come from one of 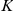 discrete populations. Population membership and allele frequencies in each population are jointly estimated from the data via a Bayesian modelling framework. A group of very widely used (e.g. [8]–[10]) current approaches powerfully extend this model by allowing individuals be admixed, i.e. to have ancestry from more than one population (e.g. [2], [11]–[17]). Individuals are assigned ancestry vectors, representing the proportion of their ancestry that comes from each of the 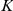 populations. Although powerful, these approaches have drawbacks – determination of 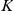 is difficult despite some technical advances [18], [19], and typically 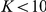 is required for satisfactory convergence, due to issues of computational cost and the presence of distinct local optima, affecting even the fastest methods such as ADMIXTURE [15]. Further, little information is provided about the relationships between inferred populations, though observing how results change with varying 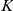 can aid insight.

The central issue that we address in this work is the fact that both PCA, and the most popular STRUCTURE-like approaches analyse single mutations individually, and do not use information about the relative positions of these mutations in the genome. However the advent of high-density variation data, together with both computational [20]–[22] and experimental [23], [24] advances in techniques for haplotype phasing offer new opportunities for researchers investigating ancestry, due to the possibility of exploiting correlated variation patterns, at sets of closely positioned markers. Markers on the same chromosome are inherited together unless separated by recombination. At a population level, this results in linkage disequilibrium (LD) between close markers that reflects a shared history of descent, invalidating the independence assumption. Haplotype based analysis has the potential to harness this information [25]–[31], but there is as yet no accepted paradigm for how to utilise shared haplotypes to infer population structure. Methods to explore admixture have been developed that aim to be robust to the presence of LD [14], [32], [33], or directly model LD patterns [34] to identify ancestry segments. However, the latter model-based approach requires representative individuals from the admixing populations to be specified in advance, so does not represent a framework for *identifying* population structure.

Here we develop and apply both non-model and model based approaches, analogous to the PCA and STRUCTURE approaches described above, that aim to use much of the information present in haplotype structure. Both approaches are based on analysing the same matrix, which we call the coancestry matrix. Although our main aim is to introduce a framework to exploit LD information where present, our methods can also treat markers independently as a limiting case. We show theoretically and in practice that in this setting, the coancestry matrix approximately contains all the information used by both PCA, and the model-based STRUCTURE-like approaches, unifying these apparently different approaches. Moreover, we show in some settings our model based approach can be more sensitive than either STRUCTURE or ADMIXTURE, and is able to reliably infer over 100 populations simultaneously. When dense marker sets are available, our haplotype-based algorithm performs substantially and uniformly better than all methods treating markers independently. We illustrate our approach using the Human Genome Diversity Panel (HGDP) dataset, comprising over 600,000 markers typed on 938 individuals. Worldwide, we show that the use of haplotype information improves separation of groups, and reveals differences in genetic ancestry even among individuals coming from the same labelled population, and not detectable by the non-LD-based equivalent approaches.

## Methods

### Chromosome painting

**Figure 1:**
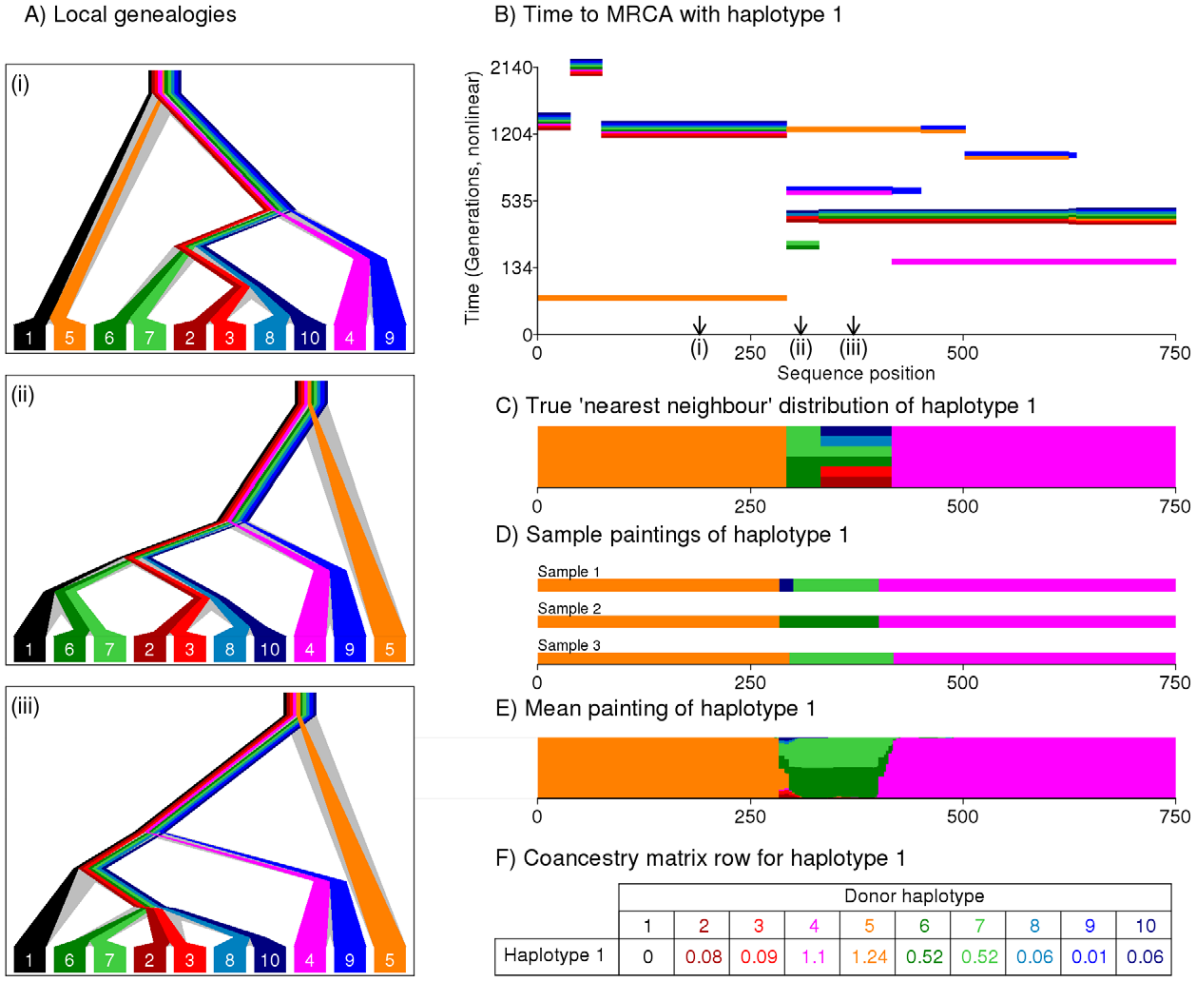
Illustration of the painting process to create the coancestry matrix. We show the process by which a haplotype (haplotype 1, black) is painted using the others. A) True underlying genealogies for eight simulated sequences at three locations along a genomic segment, produced using the program ‘ms’ [52] and showing coalescence times between haplotypes at each position. B) The Time to the Most Recent Common Ancestor (TMRCA) between haplotype 1 and each other haplotype, as a function of sequence position. Note multiple haplotypes can share the same TMRCA and changes in TMRCA correspond to historical recombination sites. C) True distribution of the ‘nearest neighbour’ haplotype. D) Sample ‘paintings’ of the Li & Stephens algorithm. E) Expectation of the painting process, estimating the nearest neighbour distribution. F) Resulting row of the coancestry matrix, based on the expectation of the painting.

Our approach attempts to capture the most relevant genealogical information about ancestry in compact form. We construct and motivate the approach using an example (Figure 1). At each locus within a chromosome, the sample history can be represented by a genealogical tree (Figure 1A), whose structure changes along the genome reflecting ancestral recombination events. First considering a single haplotype, the tree relationship to the other haplotypes is fully represented by the most-recent common ancestor (MRCA) time with each. For every individual haplotype, at each locus there exists one or more closest relative(s), which we denote their “nearest neighbour” haplotype(s) in the sample. Conceptually, we can view our haplotype as the ‘recipient’ of genetic material from a nearest neighbour ‘donor’ haplotype, who donates a contiguous DNA segment, bounded by recombination sites altering the ancestral relationship between the haplotypes (Figure 1B–1C), and thus beginning new segments, from a different ‘donor’. From the point of view of our haplotype, the chain of nearest neighbours along the genome corresponds to the most recent genealogical events, and so we assume it captures most of the information on their *current* population structure that would be provided by the complete genealogy at the locus. Further, we also assume that different nearest neighbour segments (which correspond to distinct coalescence events in regions unbroken by recombination) provide reasonably independent information on the ancestry of the individual. Finally, we aim to capture information on the joint structure of the entire dataset by constructing donor-recipient relationships for every haplotype, in the same way.

Because the set of genealogies consistent with a given dataset is complex to describe, and typically huge, approximate methods are required in order to make inference computationally practical [21], [22]. We use one such method, the Hidden Markov Model (HMM) introduced by Li and Stephens [35], which explicitly reconstructs the chromosome of a ‘recipient’ individual as a series of chunks from the other ‘donor’ individuals in the sample, using information on the types of the recipient, and potential donors, at each mutation. We assume our dataset consists of biallelic markers. We do not order the haplotypes in the same manner as the ‘Product of Approximate Conditionals’ likelihood used by Li and Stephens. Instead, we use an approach in which a single haplotype within an individual is reconstructed using the haplotypes from *all* other individuals in the sample as potential donors. This process is repeated for every haplotype in turn, so every individual is ultimately reconstructed in terms of all the other individuals. We interpret the donor of each chunk as representing a nearest neighbour of the recipient haplotype for that stretch, with each chunk representing a different nearest neighbour relationship. In the simulated setting shown in Figure 1A, haplotype 1 actually shares a common ancestor 80 generations ago with haplotype 5 (orange) from positions 0 to 284, and 150 generations ago with haplotype 4 (pink) from positions 421 to 750. In between, there is a stretch where there are multiple nearest neighbour haplotypes (Figure 1C), with the shared ancestor further back in the past. Figure 1D shows three sample reconstructions - or ‘paintings’ of the haplotype, produced by the Li and Stephens algorithm. The algorithm recovers the true genealogical relationships reasonably well, with some uncertainty about boundary regions, and with regions with multiple nearest neighbour relationships showing sampling variability. In addition to producing specific realizations of the painting process, the powerful toolkit associated with HMMs makes it possible to calculate expectations of which haplotype acts as donor to haplotype 1 as a function of position, over an infinite number of such paintings (Figure 1E). Figure 1F shows the expected number of chunks 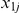 inferred from each donor 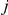 to haplotype 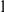, given the data. Extending this across all individuals, the matrix 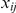 formed by all recipient rows is called the ‘coancestry’ matrix, and is summed over chromosomes. This matrix forms the basis of our inference procedure, motivated by our assumption that chunks provide independent information about ancestry. Intuitively, the coancestry matrix 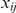 counts the number of recombination events leading to individual 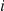 being most closely related to 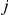, so gives a natural measure of ancestry sharing. We note that the expected lengths of the chunks donated by donor 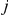 to haplotype 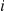, 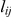, and the number of mutations 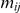 in donated chunks, may provide additional information in principle, but we do not exploit this here. To implement this approach in practice, we require previously phased (e.g. [21]) haplotype data from individuals at a defined set of loci, and (optionally) a previously estimated genetic map of the recombination distance between these loci. The Li and Stephens model has two scaling parameters, the recombination rate 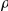 and the mutation rate 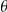, which we set to be the same for all individuals in the dataset. When analysing markers and using LD information, we estimate 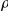 using the Expectation Maximisation (EM) algorithm [36]. Following [35], 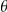 is fixed to Watterson's estimate although the parameter can also be estimated directly from the data using EM. Full details of the algorithm, which is available for download as part of the ChromoPainter package, are provided in Text S1.

One important special case is when markers are widely enough spaced as to be effectively unlinked, i.e. the recombination rate between any pair of markers is infinite. It is straightforward to produce our coancestry matrix in this setting by setting the recombination rate 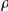 to infinity (full details in Text S1). In this setting, chunks will automatically consist of only a single marker, and thus markers are essentially independent. By painting a single biallelic marker, all potential donor haplotypes carrying the same type as the recipient individual are equally likely to actually be chosen as donors, while potential donor haplotypes carrying the other type will be very unlikely to be donors. If we additionally exclude SNPs that vary in only a single individual, which provide no information in our framework, then this ‘unlinked’ coancestry matrix can be trivially calculated analytically for any given value of 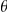. This is a symmetric matrix, and it is advantageously *not* necessary to obtain haplotypic phase (see Text S4). We therefore implement this as a special case in practice, setting 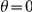. Importantly, the unlinked coancestry matrix can be calculated for any dataset, even in the case where markers in fact are in LD, in which case we view it as summarising available ancestry information, without utilising LD information. As we will explain below, this interpretation is justifiable, by considering the standard PCA and model-based approaches to analyse structure.

### Principal components analysis using the coancestry matrix

We developed and implemented an approach to perform principal components analysis (PCA), by eigenanalysis of a normalised version of our coancestry matrix (Text S4). Our method can be thought of as a natural extension of the approach of Price et al. [5] to a setting where information is available on the relationships between densely typed markers. Specifically, we show (Text S4, Proposition 1) that as 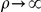, our coancestry matrix 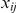 reduces to the symmetric unlinked coancestry matrix described above, 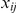 is approximately proportional to that used for the Eigenstrat PCA decomposition, and that our approach yields PCs corresponding to those calculated under the Eigenstrat PCA decomposition [5]. Thus, the Eigenstrat method corresponds approximately to a special case of our approach. In the results section, we demonstrate that in practice both methods indeed give almost identical principal components for 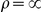. Where we analyse data as linked (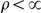), we simply apply an identical approach to the unlinked case, and in this case the identified PCs account for LD patterns, so differ.

### Model-based likelihood of the coancestry matrix

As stated above, our coancestry matrix 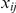 estimates the fraction of chunks in the genome that individual 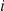
's lineage coalesces with one of the two lineages from (diploid) individual 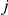 before that of any other individual. Intuitively, if individual 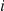 and individual 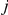 are in the same population, or related populations, they are expected to share more recent common ancestors in this manner than are pairs of individuals from historically separated groups, so 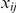 is expected to be relatively large. Even if individual 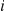 is only partially admixed with a group closely related to that which 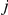 belongs to, we expect an inflation, albeit of smaller magnitude. Thus, the coancestry matrix is expected to contain rich information about population relationships. In developing a model-based approach, we have not yet implemented a model directly incorporating admixture, but concentrate on a clustering model (but where we can infer the number of clusters 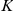, deal with a very large number of potential clusters, explore relationships between groups, and quantify ancestry sources in each group). The aim of such a model is to partition the dataset into 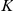 groups with indistinguishable genetic ancestry, which we interpret as individual populations. We utilise a Bayesian approach, employing reversible-jump MCMC.

To formalise this idea, we consider 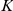 populations characterized by a donor matrix 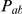, 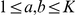 which can be thought of as a population-level coancestry matrix and gives the underlying proportion of chunks from any individual in population 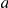 that come from population 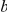. A population 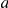 is a group of 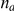 individuals where: (i) all individuals within the group are equally related, so receive the same underlying fraction 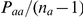 of their chunks from each of the 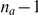 other members of the group, (ii) all individuals within the group share identical relationships with any other population 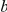, so *receive* the same fraction 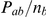 of their chunks from each member of any other population 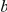, and so (iii) all individuals within the group *donate* the same fraction, 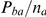, of the chunks found in any member of population 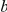. Thus, a chunk from any recipient individual within population 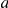 has an identical donor distribution, and an identical recipient distribution, across the sample.

Our model is now defined by our earlier stated assumption that donated chunks within an individual are independent, and no additional information is carried in their size (which for example determines the number of chunks in the genome). For individuals 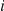, 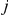 in populations 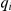 and 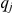 respectively, the likelihood a single chunk is donated to individual 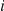 from 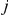 is 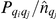 where if 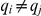, 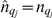 and when 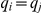, 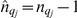 (because individuals cannot donate to themselves). Since chunks are independent, we may simply multiply the likelihood across chunks. Thus, if there are 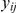 chunks in total donated from individual 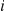 to individual 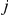, the overall likelihood for individual 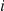 is 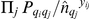. At this point, we make an approximation to the likelihood, which we partially justify later. Specifically, we replace the observed number of chunks 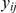 with the expected number of chunks 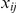 given by the coancestry matrix, which although not an integer still allows a well-defined likelihood. We treat chunks in different individuals as independent, so multiply across individuals to give a complete likelihood:
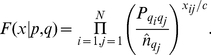
(1)Note that this likelihood depends on the data only through the terms 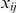 of the coancestry matrix, which we later show are approximately sufficient statistics for our inference which aids computational efficiency. In this likelihood, we have divided the chunk counts 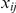 by a value 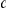 in order to account for a) non-independence of chunks in practice, and b) our substitution of the *expected* for the *observed* number of chunks copied. 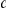 can be thought of as defining an ‘effective number of independent chunks’, which can be either less than, or greater than, the true average number of chunks - we discuss calculation of 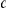 later.

In our Bayesian approach, we must model the number and distribution of the underlying populations via a prior for 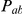. Given sufficient data, the choice of prior should only weakly affect the results (as discussed in Results, we believe this is an important strength of our approach). We choose a Dirichlet prior 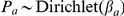 where 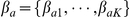, which is conjugate to the multinomial likelihood in Equation 1. The 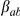 values are proportional to the a-priori expected value of each 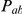, and scaling the vector 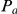 by a value 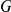 decreases the variance of all elements of 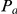 by a factor 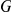. From the genealogical process, we would expect excess donor/recipient relationships within a group, i.e. that 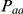 is larger than 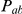 with 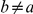. From these elements we construct the prior 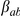 as the product of three elements: a shared variance term 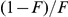 (analogous to the correlated allele frequency of Falush et al. [12]), a within population increase 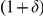 and an otherwise uniform distribution of the 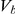 chunks donated by population 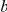 in total. Specifically,
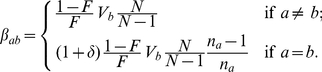
(2)The factors 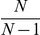 and 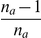 are adjustments for the fact that individuals do not act as donors to themselves. We wish to infer the parameters 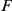 and 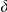 and therefore place on them a broad hyperprior based on Gamma distributions. Finally, the assignment of individuals to populations is given a Dirichlet Process Prior, which is weakly informative and allows for direct estimation of the number of populations 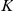. Further details are provided in Text S2.

### MCMC scheme for assigning individuals to populations

We have implemented our approach as a software package we refer to as fineSTRUCTURE. Because we have chosen the prior of 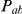 as conjugate to the likelihood in Equation 1, these population specific parameters can be integrated out analytically. The posterior probability of a population configuration, which we call a partition, is conditional on only global parameters (derived in Text S2). The target of inference is these hyper-parameters (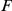 and 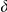) but primarily the population assignment 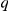. This we represent in an unordered form as a list of co-assignments, avoiding the problem of associating labels with populations. Inference for 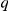 is performed using a Markov chain Monte Carlo (MCMC) algorithm closely related to that of Pella and Masuda [19] and also that implemented in the program STRUCTURAMA [37]. The space of possible partitions is explored using an algorithm which proposes new partitions that are modified versions of the previous one (see Text S3). Specifically, the partition is modified by merging or splitting populations, merging then resplitting, or moving individuals. The proposed partition is accepted, meaning that it replaces the previous one, with a probability that depends on the ratio of the likelihood with the previous partition. 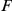 and 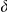 are updated within the algorithm using standard Metropolis-Hastings MCMC updates. In common with other MCMC algorithms, ours is run for a so-called burnin, after which the parameters are periodically recorded. If the algorithm is burned in and run sufficiently long, then the parameter samples converge to the posterior distribution (see e.g. [38]) of the parameters given the data, with variation found between samples reflecting posterior statistical uncertainty of parameter estimates. We test for convergence to the posterior by considering the pairwise assignment of population membership for two runs initialised with different random seeds. If the algorithm is converged then the frequency of coassignment should differ only due to Monte-Carlo error between runs.

### Estimation of the normalization parameter 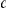


The statistical model that we have derived has a likelihood depending on the terms 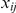 of the coancestry matrix, which are rescaled by dividing each 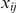 by a factor 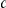 (see above). The factor 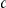 can increase or decrease depending on many factors.

Different chunks will not in practice be fully independent of each other, tending to decrease the ‘effective’ number of chunks and therefore increase 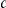. A first reason is that if individuals 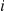 and 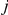 share a distinctive haplotype tract, then they will both be counted as donors for each other and the same chunk will appear twice in the likelihood, once in 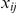 and the second in 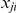. Secondly, adjacent chunks inferred on the same haplotype may not be fully independent of each other due to limitations of the Li and Stephens algorithm in modelling recombining genealogies [39] and to the non-Markovian nature of genealogical relationships themselves [40]. Thirdly, inaccuracies in the data such as phasing errors may create misleading chunk boundaries.

Conversely, by averaging over chunk assignment uncertainty in the painting step we smooth the chunk count distribution for each individual, decreasing 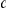 by reducing variability in chunk numbers relative to random draws. The effect is particularly large where there is a great deal of uncertainty about chunk assignment, as is the case for weakly linked or unlinked markers. In Text S4, we show that for the special case of unlinked markers (or more generally when we use the unlinked coancestry matrix for inference), appropriate choice of 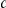 results in our likelihood being asymptotically (in large datasets not dominated by rare markers) equivalent to that of STRUCTURE, provided population structure is not too strong. See Figures S4 and S5 and Text S6 for how strong structure with truly unlinked loci affects our inference. This validates (for moderate structure) the idea of using a multinomial-form likelihood for the coancestry matrix. Further, we show analytically that the correct value of 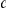 is 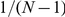 in the unlinked case.

Although we have not been able to derive such a formula for linked data, we can estimate 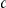 empirically. Specifically, we calculate the variance of contributions to the coancestry matrix 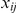 from non-overlapping chromosomal regions that are large enough that the chunk counts in each will be approximately independent. We choose 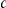 to match the mean observed variance of these contributions to that predicted by the (rescaled) multinomial model using the average number of chunks in the region. The principle of this approach is to achieve a multinomial likelihood matching the statistical uncertainty in the real coancestry matrix terms. In the case of truly unlinked data, this approach will approximately return the theoretically correct value, 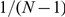. In both this and the linked setting, using extensive empirical validation we find that across a range of settings, our estimation procedure finds a conservative, close to optimal estimate for 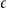 (Text S6).

Our estimation of 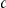 is similar in approach to the block jackknife of SmartPCA [41] though differs in many particulars, and in interpretation given we can observe 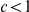 or 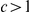 in practice. Our interpretation of 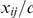 is as an effective number of independent chunks donated from 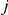 to 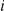.

One helpful property of this approach is that by attempting to correct for the true underlying variance of the 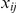, modelling deficiencies are at least partially corrected. In particular, we observe in the Results that treating markers as unlinked, by using the unlinked version of our coancestry matrix (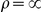), results in robust inference in both simulated and real data – even where strong association between markers in fact exists. This allows us to perform comparisons of the two approaches where we use, and do not use, LD information, on the resolution of fine-scale population structure.

### Tree building

Since the fineSTRUCTURE algorithm can identify fine subdivisions, it is often important in practice to have some indication of historical relationships amongst the inferred populations. We have found that performing inference under the full model using successively reducing values of 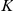 (as is commonly done in ADMIXTURE and related algorithms) does not always perform well in this setting, e.g. by splitting off highly drifted groups. Instead, we recommend an approach that performs inference at the ‘natural’ (i.e. inferred) value of 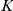, and then generates a tree of relationships amongst these populations. We start with the maximum a posteriori (MAP) state, found by taking the MCMC iteration with the highest observed posterior likelihood and then performing a number of additional hill-climbing moves to identify any merges or splits that further improve the posterior probability. Starting from this ‘best’ partition, we successively merge populations, choosing the merge giving the highest probability for the merged group at each step, resulting in a bifurcating tree relating each of the populations together. One of the biggest discriminators between populations is within-population counts, which largely reflect genetic drift occurring after a split from other groups, and are thus uninformative in choosing among group merges. In order to allow populations that contain related individuals (i.e. with high 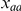) to be merged more easily, during the tree creation we replace the count matrix 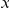 with a modified count matrix 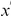 with diagonal ‘flattened’ to be the next highest value in the row, 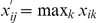 where 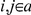 and 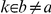. Although this ad hoc approach provides a key advantage over inference at specific 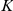 for locating functional population units, we emphasize that this tree is not based on any model of population differentiation. Results may depend significantly on sample size, and so should be treated as an approximate guide to similarity, rather than a full population history. Despite these caveats, the tree empirically performs well in capturing relationships at multiple cases when the data is approximately hierarchical.

## Results

We introduce a new approach, described in detail in Models and Methods and Texts S1, S2, S3, to analyse population structure, designed for application to large datasets, particularly where markers are in strong LD but also in other settings. To summarise, given a dataset of 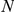 individuals, we construct an 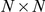 matrix 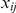, which we term the coancestry matrix, and which forms the basis of all our inference. The 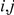 element 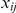 estimates the number of discrete ‘segments’ of the genome of individual 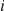 that are most closely related to the corresponding part of the genome of individual 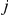. This matrix is most powerful when constructed so as to use joint information provided by tightly linked markers that are in LD. However, we can also construct an ‘unlinked coancestry matrix’ corresponding to ignoring this information, which is the correct approach if markers are widely spread across a genome. Results from using the unlinked matrix can be used to compare our approach to existing methods, and to quantify gains in information from taking into account LD information in measuring coancestry.

Given the linked or unlinked coancestry matrix, we have described how this can be used to learn about population structure: firstly, by performing PCA, and secondly, by using a model-based analysis to identify clusters of individuals with similar historical ancestry, corresponding to genetically related populations. In this section, we extensively evaluate properties of our approach in theory and using simulated data, and perform a new analysis of the HGDP dataset. We also explain how in conjunction with the clustering algorithm, analysis of the coancestry matrix reveals both differences, and details of historical interactions, among human populations in unprecedented detail.

### On large datasets, our “unlinked” method performs at least as well as PCA and STRUCTURE

To understand the properties and performance of our approach in the simplest possible setting, we begin by analysing the case where markers are treated as unlinked, i.e. our unlinked coancestry matrix. In this setting, markers may be truly unlinked, or there may be LD information being ignored. We began by analysing datasets simulated under a setting where there was no underlying population structure, both with and without tight linkage between markers (Text S6). In this setting, PCA will not give meaningful results, but encouragingly, our model-based procedure, which includes a step to estimate the effective number of chunks in the genome, correctly identified 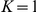 populations (Figure S1). This demonstrates our approach is robust, but we must do more to establish its power to detect structure compared to previously developed methods, and the total information present in the data. First considering the problem mathematically, we related our unlinked coancestry matrix to the 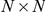 matrix used in a standard PCA approach, Eigenstrat [5]. This revealed that even though it has a rather different construction and motivation (based on the Li and Stephens algorithm [35]), our matrix is simply a linearly scaled version of the Eigenstrat matrix (Text S4, Proposition 1), implying our PCA approach in this setting ought to perform almost identically to Eigenstrat, and our coancestry matrix captures the same information as standard PCA.

**Figure 2:**
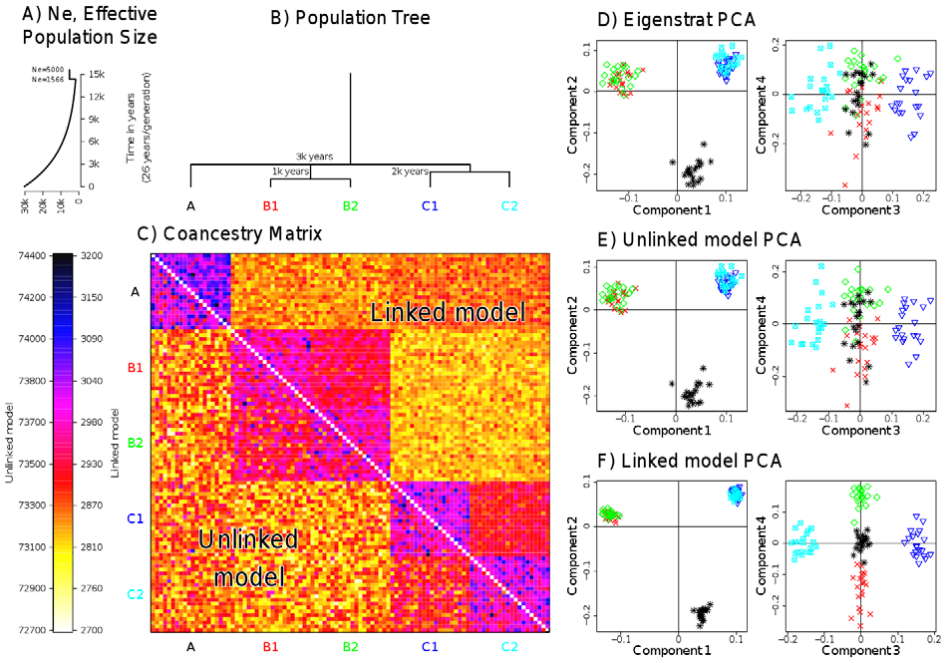
Simulated data scenario and painting results. A) Effective population size and B) population splits used for creating the simulated data. C) Coancestry heatmaps for linked and unlinked model with 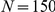 regions and 20 individuals per population, showing 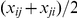 for (bottom left) the unlinked model, and (top right) the linked model; note that the linked heatmap is slightly asymmetric. D) PCA applied to the dataset using Eigenstrat on the raw SNP data. E) PCA on the coancestry matrix assuming markers are unlinked and F) linked (see text for details).

To compare the PCA approaches in practice, we constructed a simulated dataset designed to represent realistic levels of subtle population structure. We simulated data for 100 individuals according to a model containing 5 populations related in a tree-like manner with three major historical splits forming populations A, B and C two of which subsequently split (Figure 2A–2B). We used this scenario for all simulated-data comparisons, and simulated data with LD between markers. We used forward simulation of up to 200 genetic regions each 5 Mb in size, using the program SFS_CODE [42], with parameters chosen to approximate diversity found within and between European populations (see Text S5), and genetic maps based on real estimates for 10 sampled regions of the human genome [26]. We constructed the unlinked coancestry matrix for these data (which is shown for 150 regions in Figure 2C), and performed PCA both using this matrix, and using Eigenstrat on the raw data, yielding as expected almost indistinguishable results (Figure 2D–2E). These no-linkage approaches both show only incomplete separation of the most closely related pair of populations, B1 and B2; we consider the linked coancestry matrix later.

**Figure 3:**
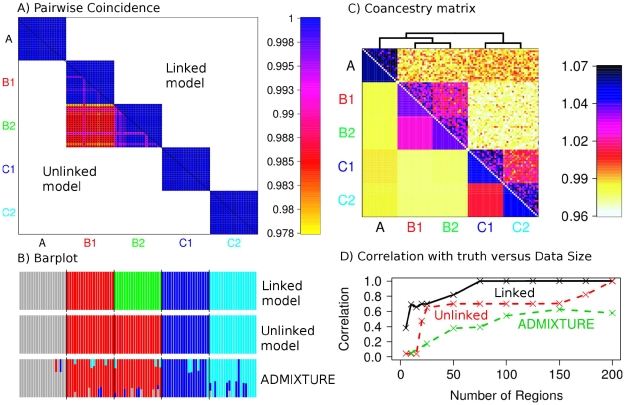
Simulated data population assignment results. A) Pairwise coincidence matrix output by fineSTRUCTURE using chunk counts calculated using (top right) the linked and (bottom left) unlinked model, for the datasets from Figure 2C. The colouring represents the posterior coincidence probability (which does not drop below 97%) and the dots represent the maximum a posteriori (MAP) probability state. B) STRUCTURE-style ‘barplot’ for the results in A as well as ADMIXTURE results for the same dataset, where each colour represents a population (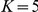, 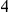 and 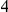 respectively). C) Aggregated coancestry matrix (bottom left, normalized to have row mean 1) for the linked model dataset (top right) rescaled from Figure 2C (also top right), shown with the inferred MAP tree (top). D) Correlation with the truth as a function of the number of 5 Mb data regions for fineSTRUCTURE linked and unlinked models, and ADMIXTURE on the same data.

We next turn to our fineSTRUCTURE model-based analysis, again considering the unlinked coancestry matrix even though strong and variable LD exists in the dataset. We first compared performance of our unlinked model to the popular ADMIXTURE [15] software (Figure 3B and 3D, details in Text S8). Encouragingly, as the number of 5 Mb regions increased from 5 to 200 we saw a monotonic performance increase for the no-linkage model, separating all groups with 200 markers. Further, our approach outperformed ADMIXTURE, with the ADMIXTURE performance levelling at around 60% correlation with the truth. In practice, we observed ADMIXTURE successfully splitting groups A, B and C and mostly splitting C1 and C2, but not B1 and B2, as detailed in Figures S6, S7, S8, S9, S10, S11. ADMIXTURE performs inference under a model where markers are treated as unlinked, and where individuals may have genomes made up of mixtures of inferred source populations, while our simulation incorporated drift between populations, but not admixture. To examine whether violations of both these modelling assumptions explain the different results, we simulated a new dataset with the same underlying population structure of 5 populations as before, but no linkage (i.e. independence) between markers within each population. We analysed these data with STRUCTURE, which uses a similar underlying model to that of ADMIXTURE, but includes a no-admixture model (Text S7). For small datasets, STRUCTURE slightly improved performance relative to our unlinked fineSTRUCTURE model, but for larger SNP numbers, fineSTRUCTURE was able to identify all population splits (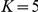) while again, STRUCTURE was able to split only populations A, B and C (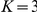). Thus, even when LD information is not used (or even present), fineSTRUCTURE can offer advantages in some settings over these existing approaches.

We sought to understand mathematically why our approach, based on only a summary of the original variation data – the unlinked coancestry matrix – equivalent to the matrix used for Eigenstrat's version of PCA, appears to perform so well relative to the earlier approaches, which carefully model each individual SNP marker (Text S4). This revealed that, surprisingly, the formulation of the likelihood of the data used by both STRUCTURE [2] and ADMIXTURE [15] can be viewed as approximately a function of *only* the terms in the coancestry/PCA matrix (under certain technical assumptions such as large datasets; Proposition 2). Under these assumptions, this result then unifies these apparently different approaches in terms of the underlying information they exploit (and suggests the PCA matrix of Eigenstrat is a particularly ‘good’ choice [43]). Furthermore, we also show that provided structure is weak (if strong, all methods are expected to find it), the multinomial likelihood used by fineSTRUCTURE is approximately the same as that used by STRUCTURE, with correct choice of the normalising parameter 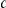 (Text S4, Proposition 4), and we find in practice that this ‘correct’ value of 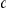 is well estimated by the jack-knife procedure described above (Figure S2). This means that at least for datasets with large numbers of loci, and ignoring linkage, we expect fineSTRUCTURE, PCA, and STRUCTURE/ADMIXTURE to all utilise similar information in the data.

What explains the different behaviour of the model-based approaches? We believe it is differences in prior models used. Both STRUCTURE and ADMIXTURE assume all underlying populations undergo separate genetic drift from some original founder group, and so this prior model penalises shared drift, for every individual marker, and so increasingly strongly as the number of loci increases. Our simulation framework (realistically, we believe), incorporates drift separate to each group, but also shared drift common to clusters of populations (caused for example, by being closer geographical neighbours). By using a more flexible prior model of structure, fineSTRUCTURE is able to separate populations C1 from C2, and B1 from B2, which the existing model-based approaches have difficulty separating even with sufficient data. By not assuming any particular form for the population-level coancestry matrix 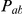, closely related groups are allowed to share genetic material, as visualised in Figure 2C.

### On dense datasets, our linked method outperforms unlinked methods

To examine improvements offered by utilising LD information, we used our linked coancestry matrix as the basis of new PCA and model-based analyses. The genetic maps used to simulated the sequence data were also used for inference in the linked model, though we note (not shown) that the conclusions still hold without this requirement. We estimated 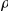 from the data by averaging estimates for 50 of the simulated regions. Using linkage information reduces the within-population variance of the coancestry matrix relative to the between-population variance (by a factor of nearly 3 in the data shown in Figure 2C) but does not change its qualitative structure. We performed PCA decomposition of the linked coancestry matrix (Figure 2F), yielding consistently tighter clustering of points, and in particular clear separation of populations B1 and B2 by the fourth principal component, compared to not using LD information (Figure 2D–2E).

In the model-based setting, linked fineSTRUCTURE strongly outperforms the unlinked version (Figure 3A), confirming the utility of LD-based inference, with only 75 regions required (Figure 3D) to correctly separate all 5 groups vs. 200 when ignoring linkage. Encouragingly, performance improves more dramatically for fewer regions, when structure is at the limits of detection. Examination of a particular case (Figure 3A–3B) with 150 regions shows only a partial separation using unlinked fineSTRUCTURE of the most similar groups B1 and B2, analogously to the PCA result. ADMIXTURE (Text S8) also fails to identify this population split. In practical applications, given a finite genome size, using linkage information will therefore be expected to allow clear identification of more subtle (‘fine’) structure than is detectable otherwise, as we show in the next section. Figure 3C shows the linked model coancestry matrix averaged over populations (using the model-based assignment of individuals to populations), as well as a tree (which is correct except that population A is not equidistant between B populations and C populations), inferred as described in Models and Methods. We view this coancestry matrix and the tree as the ‘outcome’ of our model-based inference procedure – it details groups found, their inferred relationship, but also shows the inferred extent of haplotype sharing between groups, showing for example groups that share closer genetic relationships. As we explain below, we believe that in practical applications, this representation can reveal interesting features of underlying structure.

### Worldwide HGDP data analysis identifies novel features of human populations

**Figure 4:**
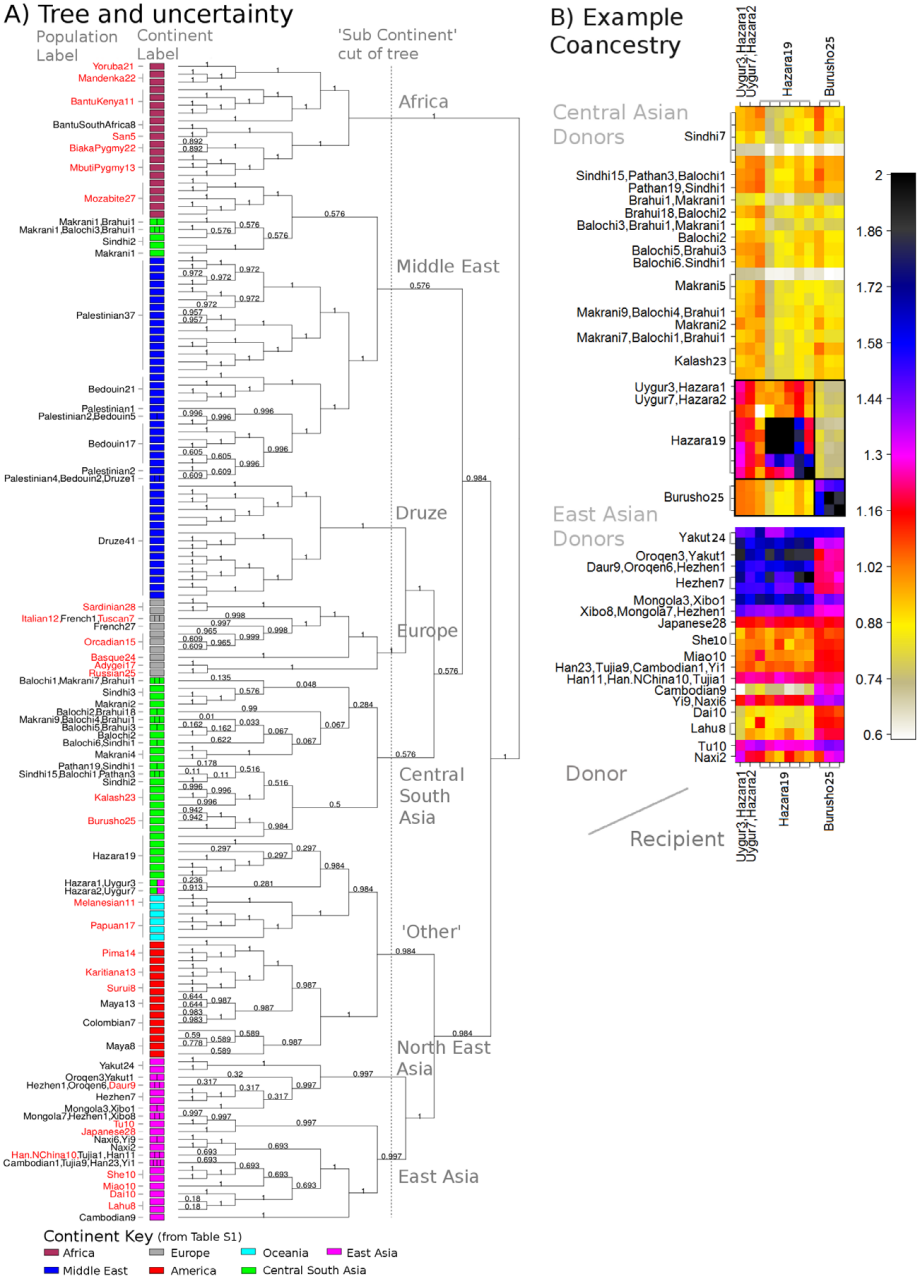
World HGDP results summary. A) Relationship between populations for the whole world data. Each tip corresponds to a population; labels include the number of individuals and are coloured red if all individuals within that label are found in a single clade. See text for an interpretation of the values on the edges; the cut defines the ‘sub-continents’ discussed in the text. B) Transposed coancestry matrix for the Hazara and Burusho (in full: Figure S14), showing CentralSouthAsia and EastAsia donors, which are each normalised to have mean donation rate of 1. The box shows the ‘diagonal’ drift component.

We analysed the pattern of population structure in the Human Genome Diversity Project (HGDP) dataset [9] of 640,698 autosomal SNPs typed in 938 individuals sampled from 53 different labelled groups, with 5 to 46 sampled individuals per group. Complete inferred-phase haplotypes ([21], [44]) were downloaded from http://hgdp.uchicago.edu/. Estimated b36 recombination rates [26] were downloaded from the HapMap website (http://www.hapmap.org). Despite the size of the dataset, the fineSTRUCTURE algorithm (Text S10) converges in independent runs (Figure S25) to a solution with 149 populations in the most probable posterior state using the data calculated based on the linked model (Figure 4A). Our tree building algorithm aims to represent the relationships among the groups and in the tree, for which almost half (25 of 53) of the original labelled groups exactly correspond to a single clade in the tree, 9 corresponding exactly to a single inferred population. In other cases, geographically neighbouring groups (e.g. several groups sampled in Pakistan) are not separated, implying sample labels do not perfectly correspond to identifiable ancestry signals. Higher up the tree, branches correspond to large continental-level groups, similar to those seen before [45].

In general, many groups are not related through simple hierarchical ‘tree-like’ drift, but also through complex admixture events. These relationships are captured directly in our representation by the coancestry matrix. Although this is high-dimensional even after clustering individuals into groups, and in future we think it is important to incorporate admixture in our modelling framework, we nevertheless believe the very complex structure of the data itself means visual examination of the coancestry matrix provides important insights using linkage information. Previous analysis of the worldwide HGDP using ADMIXTURE, and to an extent PCA, has identified signals of admixture [9], [28], [45] in certain groups. In practice, the number of groups that these methods can infer is typically limited to 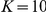 or fewer, resulting in limited resolution in identifying the detail of such admixture events. In addition, both PCA and ADMIXTURE analyses do not consistently signal the extent of genetic drift in the dataset. Follow-up ‘regional’ analyses, for example focussing on Europe, partially address these issues for drift and admixture within such regions, but not across larger distances. The linked coancestry matrix allows simultaneous visualisation of drift, and admixture, and fine-scale resolution for both (Figure S14). For example (Figure 4B), previous observations [46] of both Central and East Asian ancestry in the Hazara (from Pakistan) can now be refined. The coancestry matrix demonstrates strong haplotype sharing of the Hazara from other Pakistani groups (e.g, the Pathan) as well as varying continuously in admixture fraction with groups from today's north-east Asia (e.g. the Mongola). This provides direct genetic evidence corroborating historical evidence [47] of ancestry sharing between the Hazara and the Mongols. The Burusho, another Pakistani group showing East Asian admixture, are separated from the Hazara by fineSTRUCTURE, but have relatively less North-East Asian DNA, implying distinct admixture histories for these two groups. Many other HGDP admixture signals could be analysed similarly.

Although fineSTRUCTURE performs well on the global dataset, for easier visualisation of results, we developed an approach analysing structure in only sub-regions of the data, but based on the same (worldwide) coancestry matrix as before. In practice, we found this had the second advantage of a small increase in resolution, while retaining the ability to identify many long-range population relationships. This increase in power is related to our prior model – we assume ancestry proportions are independent across groups, while in fact worldwide historical relationships among populations result in correlations in these vectors. Although the prior is overwhelmed by the data for clear splits (unlike that used by other approaches), our algorithm nevertheless can merge very similar groups. Within a subregion of the world, however, differences in ancestry proportions are much closer to independent, potentially improving precision.

For a regional analysis, we chose to split the dataset into eight regions, approximately corresponding to ‘sub-continents’, based only on the results of the merging algorithm used to produce the population tree (Figure 4A). Each geographic region is analysed individually by fineSTRUCTURE under the full model, with other regions considered only via donation of genetic material when pooled into seven overall counts, corresponding to the total received from each (the number of individuals is also used). This approach is a balance of retaining broad-scale information relating to admixture from external sources, while substantially reducing dimensionality. Figure S15 shows the tree for these results which is broadly similar to Figure 4A though differs in some particulars (for example Maya and Colombian are now split but BantuKenya are not) partly due to different ‘diagonal flattening’ restrictions across subcontinents. 226 populations are now found, many of which may simply be related individuals (e.g. within the Druze) whilst others reflect real but subtle population structure.

**Figure 5:**
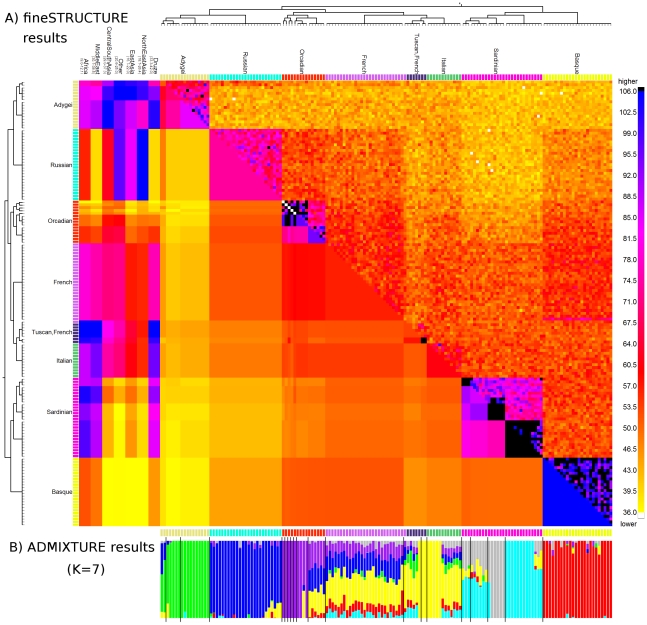
Coancestry heat map for the Europe sub-continent. A) (bottom left) population averages, (top right) the raw data matrix, and (left) chunks from other sub-continents. To symmetrise the matrices we show the average of the donor/recipient chunk counts; read the row *and* column for an individual to see their full profile. The tree has the same interpretation as Figure 4, and the heatmap between individuals in Europe has the same interpretation as Figure 2C, with extremely high (black) and low (white) values capped. Each continent has its own scale (top), with the lowest value in yellow and the highest in blue. B) ADMIXTURE barplot for the same dataset.

We focus on the European results as an example (Figure 5A), with other continents shown in Figures S16, S17, S18, S19, S20, S21, S22, S23, S24. Convergence in all cases was excellent (Figures S26, S27, S28, S29, S30, S31, S32, S33), despite significant uncertainty. The smaller scale of the problem here allows more detail of results to be discussed, but also meaningful comparison with other approaches. We identified 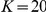 populations with fineSTRUCTURE, identifying (and in some cases further splitting) the 8 labelled European groups precisely, apart from one French individual showing an ancestry pattern closer to the Tuscans in the dataset (and visually intermediate from both). Again, examination of the identified coancestry matrix parameters is helpful in revealing relationships among the groups, and with outside populations. For example a large coancestry value within some populations (along diagonal blocks in the coancestry matrix) can be interpreted as strong genetic drift, which appears in some groups (e.g. the island Orcadian and Sardinian populations) but is absent in the French. The multiple populations found for Orcadians, Sardinians and Tuscans, with particular subgroups having significantly elevated coancestry even within the same label, suggests more recent kinship perhaps related to geography (which we do not have additional information on). The Adygei (from the Caucasus) are split into three groups, which instead differ mainly in their levels of Russian admixture within Europe, and of Central and East Asian ancestry from outside. Similarly, Tuscans are split from a different North Italian group, due to a very subtle ‘drift’ signal along the diagonal, but mainly by having more African and Middle Eastern ancestry (corroborating results on mitochondrial DNA [48]). Similar signals are seen across other continents.

We applied ADMIXTURE to the same HGDP European data as analysed by fineSTRUCTURE (Text S9). Although the populations are very subtle and ADMIXTURE cross-validation implies 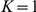 (Figure S13), we still obtained meaningful results with 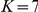 (Figure 5B) and fewer (Figure S12) populations, but noise for higher 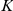. As expected for this powerful approach, ADMIXTURE gave useful information on European groups, with clear separation of Adygei, Russian and Basque for example and some, but not all, of the within-population splits represented. Based on this analysis, it is not possible to separate certain groups, e.g. the Tuscans and Italians, where inferred non-admixed and admixed individuals are spread among both groups, neither corresponding to sample labels nor supported by other analyses (including ADMIXTURE at different 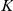), and thus results may reflect modelling uncertainty. More generally, the French, Italians/Tuscans and some Orcadians are closer to lying along an admixture continuum in this analysis, while appearing much more cleanly separated, and homogeneous in ancestry makeup, in the linked coancestry matrix (which has identifiable ‘blocks’ of colour for these groups). As expected from the earlier simulations, the differences with fineSTRUCTURE seem to be concentrated in the more subtle splits, and also in the fact that ADMIXTURE analysis cannot here easily benefit from information on outside genetic contributions, e.g. to distinguish a third Adygei group. Finally, for the subtle structure present here, care clearly must be taken in interpreting ADMIXTURE results – in each of the Orcadian, Italian/Tuscan and Sardinian groups, some individuals appear genetically mixed and others do not, while the coancestry matrix does not support such a genuinely distinct relationship.

**Figure 6:**
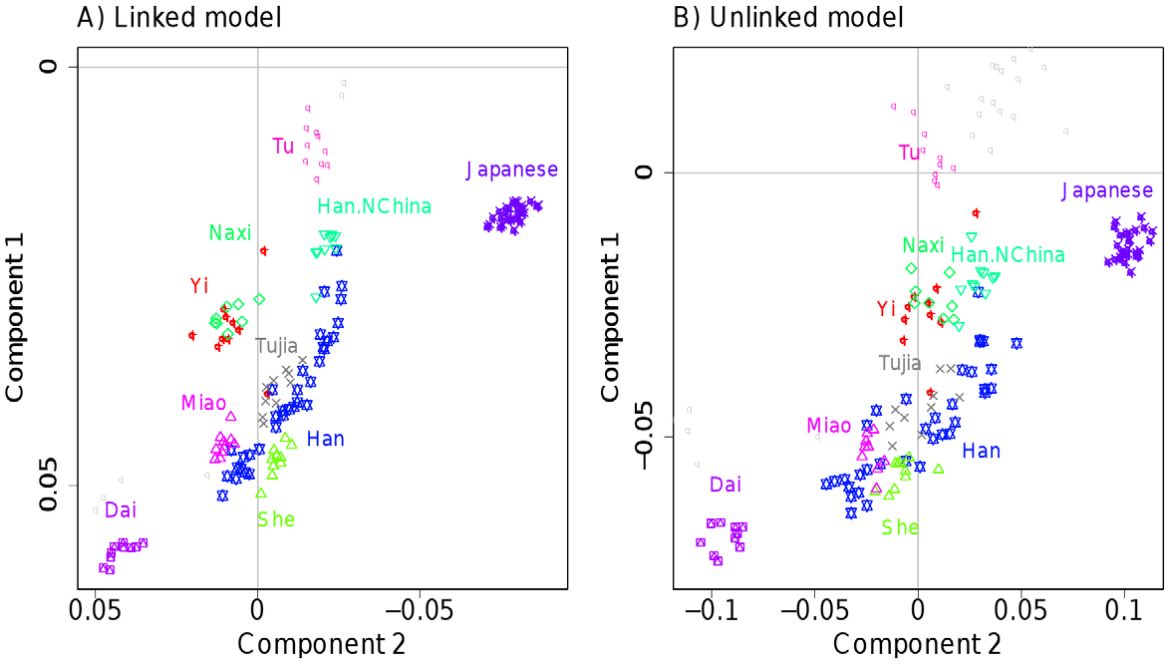
PCA for East Asia HGDP data. The first 2 PCA components of the East Asian ‘continent’ as defined in Table S1 are shown for A) the linked model and B) the unlinked model. Only the named labels are displayed for clarity; Figure S37 shows the full set. Further structure will be present in other principal components (not shown).

In addition to using fineSTRUCTURE, we also used our linked (and unlinked) PCA approaches to analyse the data for Europe and other continents (Figures S34, S35, S36, S37, S38, S39, S40). Results in general were consistent with our simulations and with the model-based analysis, giving better separation of groups for the linked PCA version, e.g. clean separation of Italians and Tuscans only when LD information is utilised (Figure S38). Figure 6 illustrates this improvement for a subset of populations in central East Asia. Only the linked model shows clear separate clusters for Miao, She and Tujia, or any obvious separation of Tujia and Han. The latter group are revealed as lying along a line, much noisier in the unlinked case and suggesting variable levels of coancestry between Han individuals and other Chinese groups, presumed to have occurred during the North to South spread of the Han [49], and directly visible in the coancestry matrix (Figure S10).

**Figure 7:**
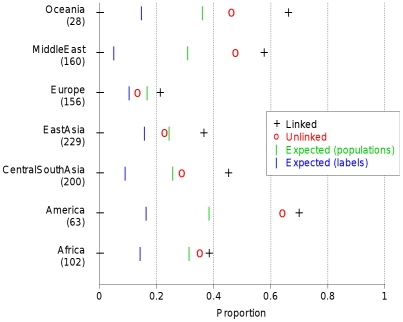
Half-matching using correlations for HGDP data. For each continent, we show the proportion of times in which two sets of chromosomes of a particular individual are matched correctly based on similarity of their coancestry profile. Coancestry profiles are calculated using a training set as described in the text. Results for coancestry matrices are calculated using correlation between individuals based on the linked and unlinked models. Also shown are the expected success in clustering if individuals within the same label or same inferred (linked results) fineSTRUCTURE population each had the same ancestry profile.

The strongest advantage to using the linked model is in separating subtly different groups, and we see many cases in our data where labelled groups are split into smaller populations by fineSTRUCTURE, but although these show features consistent with their representing genuine ancestral differences, we do not have additional information, for example on geography to confirm these populations. We therefore devised a scheme to overcome our incomplete information, using the fact that although completely unlinked, two approximately equally sized halves ‘A’ and ‘B’ of an individual's genome automatically share all sampling details, and thus have the same underlying ancestry. Examining similarity in ancestral profiles for the two halves thus provides an indication of whether ancestry differences observed (from half the genome) are genuine, at the finest possible scale. Specifically, we analysed half of the individuals at a time (splitting the dataset approximately evenly for each label), painting their chromosomes using an identical donor set consisting of the other half of the sample, so chunk counts for individual ‘A’ or ‘B’ halves are comparable across individuals. For each individual ‘A’ half, we paired with the most correlated individual ‘B’ half, and recorded the fraction of times this ‘B’ half came from the same individual (Figure 7), and compared this to random chance when using population or label groupings. The results validate our populations as reflecting genuine ancestral differences, pairing halves within clusters more of the time than using labels alone. Interestingly, we paired up genomic halves within individuals consistently more often than predicted by than our clustering (and uniformly more often using linked than unlinked information) demonstrating that human population structure exists at finer scale than the clustering detects, and is most powerfully identified using linkage information.

## Discussion

Partial or complete barriers to mating create groups with distinct genetic ancestry, or, in the present terminology, populations. In our approach, we assume that chromosomes within a particular population have characteristic probabilities of sharing stretches of similar DNA from individuals in their own and in other populations, and view these probabilities as defining population composition and relationships. To infer groups, we first reduce data dimensionality by estimating the relationships among all pairs individuals using a “coancestry matrix”, which is central to our method and based on ‘painting’ the chromosomes of each individual [35]. Loci can be treated as linked or unlinked in the genome. In the unlinked case, we have shown that in theory and in practice, our model-based (MCMC) and PCA approaches are very closely related to the previous approaches exemplified by STRUCTURE and Eigenstrat [2], [5], and that the parametric and non-parametric approaches can all be thought of as, approximately, interpretations of information present in the coancestry matrix. This helps explain previous observations [50] that structure is frequently detectable using both types of approach, or neither. Other approaches to summarizing matrices, such as sparse value decomposition, might bring out additional features [43].

We have also shown that the linked approach substantively improves performance, where LD information is present among tightly packed markers, achieving a resolution in the HGDP that is to our knowledge unprecedented. Intuitively, we believe that the underlying reason is that using haplotype sharing identifies relationships among individuals in the recent past much more strongly than individual ancient SNP sharing, enabling more subtle, recent population structure to be captured [31]. This does not mean the approach is optimal – additional improvements may be found by utilising information (within our framework) on the size of shared haplotypes, mutations private to particular groups, and haplotypic sharing further back in time. We believe the advantages offered by exploiting haplotypic information will continue to grow as full sequence data becomes predominant [51].

In practical implementation, our approach uses two initial, parallelizable analyses: a phasing step, common in modern population genetic analyses, and a subsequent chromosome painting step, both run once on a given dataset, and feasible for datasets with millions of markers using computer clusters. Subsequent steps using the resulting coancestry matrix have computational time depending only on the number of individuals, which with our efficient algorithmic implementation enable us to, for example, analyse far larger numbers of populations – hundreds in the HGDP - than other approaches that reanalyse each mutation at each iteration. We observed a substantial performance improvement for the linked model, when applied to the HGDP data phased jointly using fastPHASE [21], despite inevitable errors in the haplotypes produced by all such phasing approaches. However, we caution against naively combining and analysing datasets phased *separately*, or by different approaches, which may introduce spurious differences in haplotype composition.

In the model-based approaches discussed here, we have described how the coancestry matrix captures key relationships among groups. However, future approaches may aid interpretation of results, and power, by explicitly modelling the processes of drift, and subsequent admixture, among identified populations and their effect on this matrix. The theory developed here for the unlinked case suggests a close connection between population level genetic drift and the coancestry matrix. Although this (like average pairwise coalescent times [4]) will not uniquely specify historical events, genetic drift specific to a population will have the effect of elevating the within-population coancestry value, while admixture causes a population to become more similar, both as a donor and recipient to the group it is admixing with. Relating identified groups in this manner and developing new ways of representing population structure are both needed, given both the very fine stratification (into 226 groups) achieved by the approach and the half-matching results demonstrating even more structure present in the data. Allowing individuals to show continuous variation in proportions of ancestry from multiple groups might capture this signal [2]. However, because we observe a drift signal private to most of our identified groups, we believe a necessary but difficult modelling challenge is to incorporate successive rounds of genetic drift, admixture, further genetic drift, and even familial relationships into such models.

Overall, our results demonstrate we have not yet reached the limits of the information available using genetic information, and particularly the precision with which ancestry sources can be determined. As full sequence data and larger sample sizes become increasingly available, we anticipate resolution will improve further beyond the level of countries, to regions within countries in many cases, and this will be of value in a range of settings. The methods described here can produce highly accurate clustering and sensible choices of the number of populations in humans and other species, and can be applied to full genome sequences for thousands of individuals.

The algorithms described in this article have been implemented in computer software packages ChromoPainter and fineSTRUCTURE, which are available at http://www.paintmychromosomes.com/.

## Supporting Information

Figure S1Correlation with truth for Unlinked data. 15000 non-rare (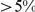 allele frequency) unlinked SNPs were simulated, and inference considered with a varying number of individuals and with varying chunk scaling 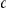, when there is no true population structure. Black indicates perfect correlation, which is always achieved at the theoretical (black line) and empirical estimated (dots) values of 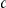. (Note that at 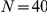 the correlation is perfect at the theoretical value of 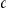, but not at 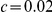, the nearest point on the grid.)(TIFF)

Figure S2Correlation with the truth for linked data. A varying number of individuals with varying chunk scaling 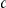 are considered, with the 5 populations described in Figure 2 of the main text (and 150 regions of data). Left (a) is for the linked model, Right (b) is for the unlinked model. The empirical estimated values of 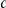 are shown as dots.(TIFF)

Figure S3Correlations within the coancestry matrix for unlinked data. Left: the raw coancestry matrix for the same scenario as simulated in the main text but with 15000 unlinked SNPs. Centre: the renormalized coancestry matrix based on the true population distribution. Right: The difference, highlighting the correlated nature of the error terms for the coancestry matrix (there are differences for the merged B1 and B2 populations only). Top: These matrices based on the ‘true’ population structure given by the labels. Bottom: These matrices based on merging the most recent split, setting 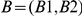.(TIFF)

Figure S4Correlation with truth for Unlinked data with strong population structure. This is a demonstration of how our model breaks down in the presence of strong population structure and *unlinked* data, and our method for fixing this. This figure shows the correlation with the truth for 15000 non-rare (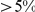 allele frequency) unlinked SNPs under the simulation demographic model described in the main text. Left: results for the raw data. Right: results for the modified data matrix 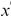 as described above.(TIFF)

Figure S5Correlation with truth for fineSTRUCTURE and STRUCTURE. (black) is fineSTRUCTURE and (red) is STRUCTURE, considered as a function of the number of unlinked SNPs. Data are simulated as described, with all SNPs having minor frequency 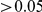. The fineSTRUCTURE results are based on the unlinked model as described above, and the STRUCTURE results are based on the no-admixture model using the ‘F model’ prior started at the best possible configuration for a particular K. Optimal correlations are obtained at this configuration when there is no uncertainty in the assignment. Note that the scale is logarithmic to emphasise the behaviour with few SNPs.(TIFF)

Figure S6 ADMIXTURE results for simulated data at 25 linked regions. Top: cross-validation error (lower is better). True populations are separated by a black line. The maximum correlation with truth is obtained at K = 3.
(TIFF)

Figure S7 ADMIXTURE results for simulated data at 50 linked regions. Top: cross-validation error (lower is better). True populations are separated by a black line. The maximum correlation with truth is obtained at K = 3.
(TIFF)

Figure S8 ADMIXTURE results for simulated data at 75 linked regions. Top: cross-validation error (lower is better). True populations are separated by a black line. The maximum correlation with truth is obtained at K = 3.
(TIFF)

Figure S9 ADMIXTURE results for simulated data at 100 regions. Top: cross-validation error (lower is better). True populations are separated by a black line. The maximum correlation with truth is obtained at K = 4.
(TIFF)

Figure S10 ADMIXTURE results for simulated data at 150 regions. Top: cross-validation error (lower is better). True populations are separated by a black line. The maximum correlation with truth is obtained at K = 4.
(TIFF)

Figure S11 ADMIXTURE results for simulated data at 200 regions. Top: cross-validation error (lower is better). True populations are separated by a black line. The maximum correlation with truth is obtained at K = 4.
(TIFF)

Figure S12 ADMIXTURE results for the HGDP Europe dataset. A range of K is considered as described in the text. Dashed lines separate fineSTRUCTURE populations, solid lines separate labelled populations. fineSTRUCTURE agrees with all labelled populations with the exception of the Tuscan/French.
(TIFF)

Figure S13ADMIXTURE cross validation error as a function of 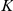. The recommended procedure is to choose the 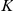 with the minimum cross-validation error, here 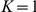.(TIFF)

Figure S14 Whole world HGDP coancestry matrix. Some population labels are omitted for clarity; this has only been done when the neighbouring population contains the same labels and the exact distribution is recoverable from the tree and Figure 4 of the main text. The colour scale is non-linear, and population sizes have been square-rooted for clarity.
(TIFF)

Figure S15 “Sub-continental” tree for all HGDP populations. Inference was performed in separate subcontinents groupings as defined in Figure 4 of the main text, with details for each subcontinent given in Figures S16, S17, S18, S19, S20, S21, S22, S23, S24.The interpretation is the same as Figure 4 of the main text (except that probabilities have been removed for clarity).
(TIFF)

Figure S16 “Sub-continental” coancestry matrix. Groupings as defined in Figure 4 of the main text. Recipient groups are on the left. Note that Africa has been capped, and copies 232 chunks to itself.
(TIFF)

Figure S17 “Sub-continent” of Africa coancestry matrix. (bottom left) the Population coancestry matrix and (top right) the Individual coancestry matrix.
(TIFF)

Figure S18 “Sub-continent” of CentralSouthAsia coancestry matrix. (bottom left) the Population coancestry matrix and (top right) the Individual coancestry matrix.
(TIFF)

Figure S19 “Sub-continent” of Druze coancestry matrix. (bottom left) the Population coancestry matrix and (top right) the Individual coancestry matrix.
(TIFF)

Figure S20 “Sub-continent” of EastAsia coancestry matrix. (bottom left) the Population coancestry matrix and (top right) the Individual coancestry matrix.
(TIFF)

Figure S21 “Sub-continent” of Europe coancestry matrix. (bottom left) the Population coancestry matrix and (top right) the Individual coancestry matrix.
(TIFF)

Figure S22 “Sub-continent” of MiddleEast coancestry matrix. (bottom left) the Population coancestry matrix and (top right) the Individual coancestry matrix.
(TIFF)

Figure S23 “Sub-continent” of NorthEastAsia coancestry matrix. (bottom left) the Population coancestry matrix and (top right) the Individual coancestry matrix.
(TIFF)

Figure S24 “Sub-continent” of “Other” populations. ‘Other’ is defined as America, Oceania and some Asian individuals. (bottom left) the Population coancestry matrix and (top right) the Individual coancestry matrix.
(TIFF)

Figure S25 Whole HGDP pairwise coincidence matrix. (bottom left) run 1 and (top right) an independent run 2. It is recommended to view this figure online and use zoom tools.
(TIFF)

Figure S26 Africa pairwise coincidence matrix. (bottom left) run 1 and (top right) independent run 2.
(TIFF)

Figure S27 CentralSouthAsia pairwise coincidence matrix. (bottom left) run 1 and (top right) independent run 2.
(TIFF)

Figure S28 Druze pairwise coincidence matrix. (bottom left) run 1 and (top right) independent run 2.
(TIFF)

Figure S29 EastAsia pairwise coincidence matrix. (bottom left) run 1 and (top right) independent run 2.
(TIFF)

Figure S30 Europe pairwise coincidence matrix. (bottom left) run 1 and (top right) independent run 2.
(TIFF)

Figure S31 MiddleEast pairwise coincidence matrix. (bottom left) run 1 and (top right) independent run 2.
(TIFF)

Figure S32 NorthEastAsia pairwise coincidence matrix. (bottom left) run 1 and (top right) independent run 2.
(TIFF)

Figure S33 “Other” populations pairwise coincidence matrix. (bottom left) run 1 and (top right) independent run 2.
(TIFF)

Figure S34 PCA for the continent of Africa. The first two components are shown; furhter structure will be present in the higher components.
(TIFF)

Figure S35 PCA for the continent of America. The first two components are shown; furhter structure will be present in the higher components.
(TIFF)

Figure S36 PCA for the continent of CentralSouthAsia. The first two components are shown; furhter structure will be present in the higher components.
(TIFF)

Figure S37 PCA for the continent of EastAsia. The first two components are shown; furhter structure will be present in the higher components.
(TIFF)

Figure S38 PCA for the continent of Europe. The first two components are shown; furhter structure will be present in the higher components.
(TIFF)

Figure S39 PCA for the continent of MiddleEast. The first two components are shown; furhter structure will be present in the higher components.
(TIFF)

Figure S40 PCA for the continent of Oceania. The first two components are shown; furhter structure will be present in the higher components.
(TIFF)

Table S1 Population labels assigned to “continents” for PCA.
(PDF)

Text S1 Mathematical description of the Painting algorithm.
(PDF)

Text S2 Derivation of the fineSTRUCTURE Partition Posterior probability.
(PDF)

Text S3 Mathematical details of the fineSTRUCTURE MCMC moves and acceptance probabilities.
(PDF)

Text S4 Theory linking PCA, STRUCTURE and fineSTRUCTURE. This includes Propositions 1–4 and a brief summary of what they imply.
(DOCX)

Text S5 Simulation procedure for linked data using SFS_CODE.
(PDF)

Text S6Empirical evaluation procedure for the scaling parameter 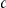. This includes the simulation procedure for unlinked data, and the empirical validation that our procedure correctly identifies 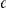.(PDF)

Text S7 Empirical comparison of fineSTRUCTURE to STRUCTURE.
(PDF)

Text S8 Details of the ADMIXTURE linked simulation evaluation procedure.
(PDF)

Text S9 Details of the ADMIXTURE HGDP analysis.
(PDF)

Text S10 Results for HGDP data. These comments interpret Figures S14, S15, S16, S17, S18, S19, S20, S21, S22, S23, S24, S25, S26, S27, S28, S29, S30, S31, S32, S33, S34, S35, S36, S37, S38, S39, S40, i.e. the fineSTRUCTURE and PCA-based continent and sub-continent analyses for the HGDP dataset.
(PDF)
