# Supplementary figures and images for: Inference of Population Structure using Dense Haplotype Data
Source: PLoS Genet. 2012 Jan 26;8(1):e1002453. doi: 10.1371/journal.pgen.1002453 (PMC3266881; doi:10.1371/journal.pgen.1002453)

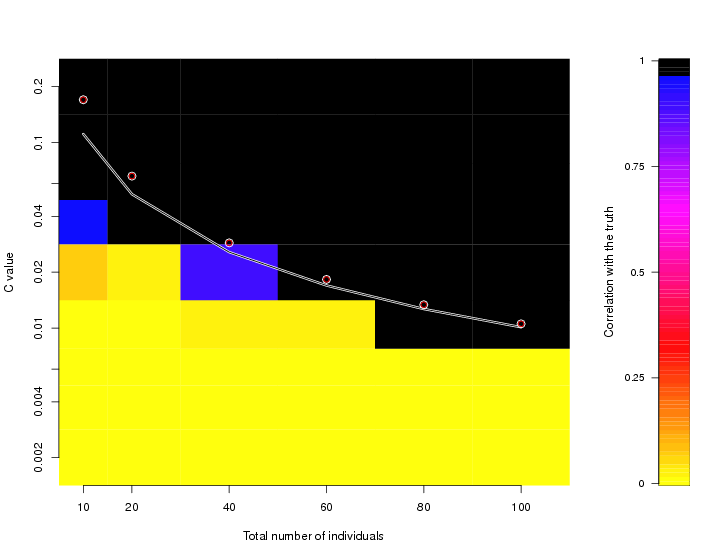

Supplement: Figure S1 — Correlation with truth for Unlinked data. 15000 non-rare ( allele frequency) unlinked SNPs were simulated, and inference considered with a varying number of individuals and with varying chunk scaling , when there is no true population structure. Black indicates perfect correlation, which is always achieved at the theoretical (black line) and empirical estimated (dots) values of . (Note that at the correlation is perfect at the theoretical value of , but not at , the nearest point on the grid.) (TIFF) [file pgen.1002453.s001.tiff]

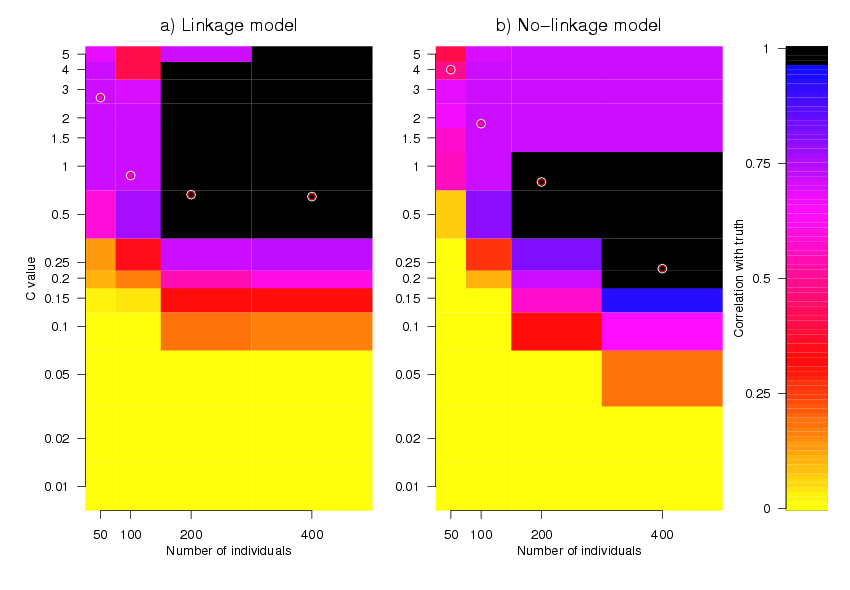

Supplement: Figure S2 — Correlation with the truth for linked data. A varying number of individuals with varying chunk scaling are considered, with the 5 populations described in Figure 2 of the main text (and 150 regions of data). Left (a) is for the linked model, Right (b) is for the unlinked model. The empirical estimated values of are shown as dots. (TIFF) [file pgen.1002453.s002.tiff]

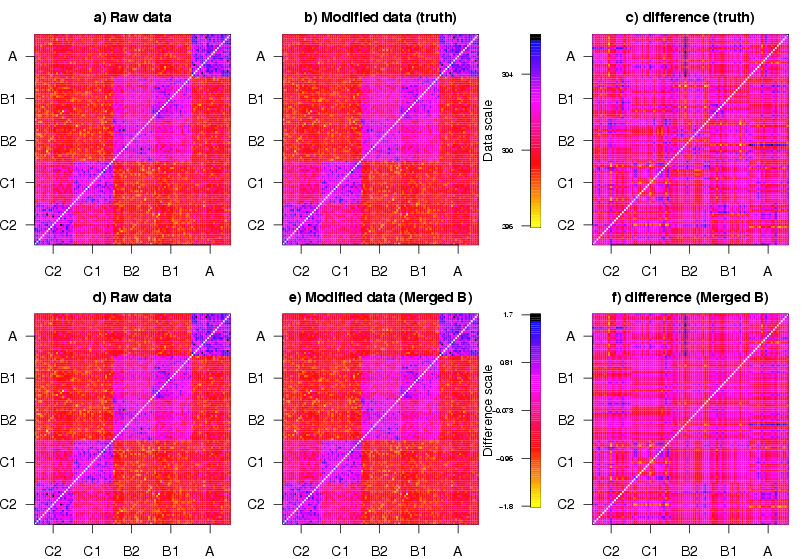

Supplement: Figure S3 — Correlations within the coancestry matrix for unlinked data. Left: the raw coancestry matrix for the same scenario as simulated in the main text but with 15000 unlinked SNPs. Centre: the renormalized coancestry matrix based on the true population distribution. Right: The difference, highlighting the correlated nature of the error terms for the coancestry matrix (there are differences for the merged B1 and B2 populations only). Top: These matrices based on the ‘true’ population structure given by the labels. Bottom: These matrices based on merging the most recent split, setting . (TIFF) [file pgen.1002453.s003.tiff]

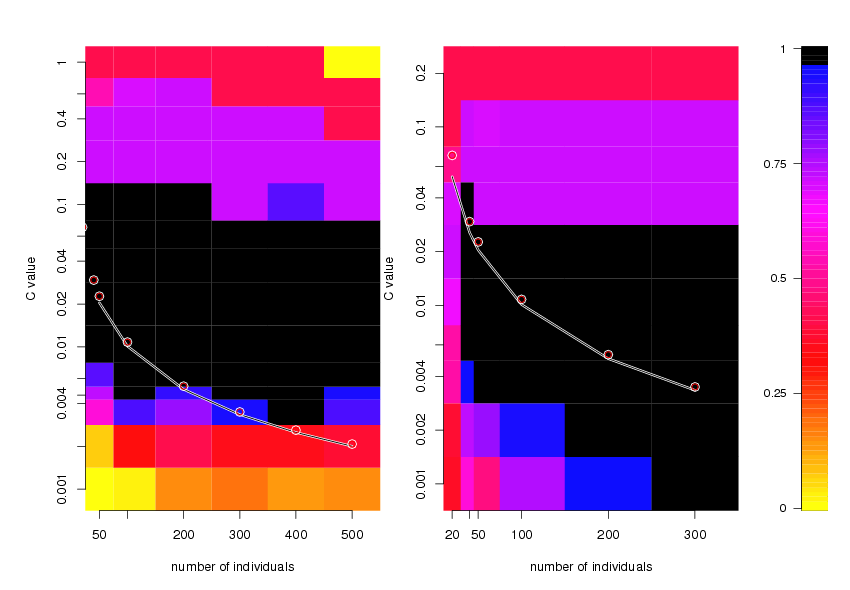

Supplement: Figure S4 — Correlation with truth for Unlinked data with strong population structure. This is a demonstration of how our model breaks down in the presence of strong population structure and unlinked data, and our method for fixing this. This figure shows the correlation with the truth for 15000 non-rare ( allele frequency) unlinked SNPs under the simulation demographic model described in the main text. Left: results for the raw data. Right: results for the modified data matrix as described above. (TIFF) [file pgen.1002453.s004.tiff]

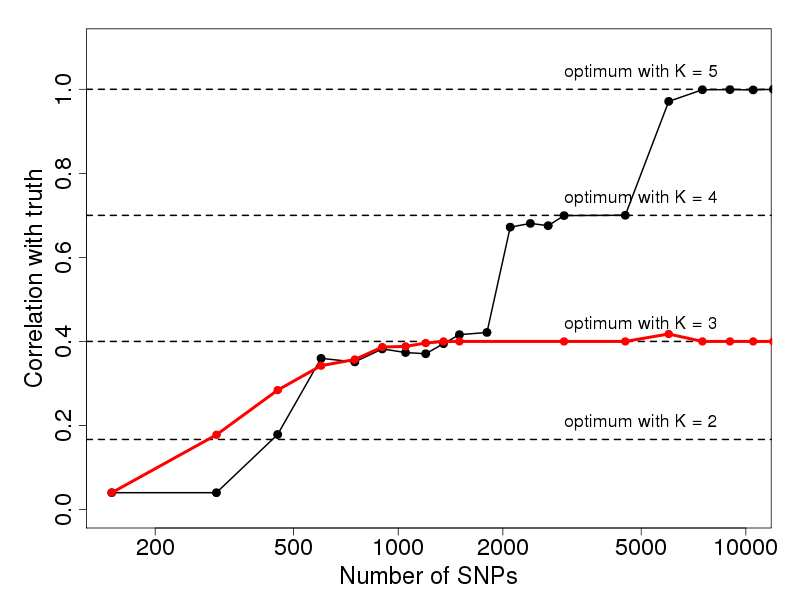

Supplement: Figure S5 — Correlation with truth for fineSTRUCTURE and STRUCTURE. (black) is fineSTRUCTURE and (red) is STRUCTURE, considered as a function of the number of unlinked SNPs. Data are simulated as described, with all SNPs having minor frequency . The fineSTRUCTURE results are based on the unlinked model as described above, and the STRUCTURE results are based on the no-admixture model using the ‘F model’ prior started at the best possible configuration for a particular K. Optimal correlations are obtained at this configuration when there is no uncertainty in the assignment. Note that the scale is logarithmic to emphasise the behaviour with few SNPs. (TIFF) [file pgen.1002453.s005.tiff]

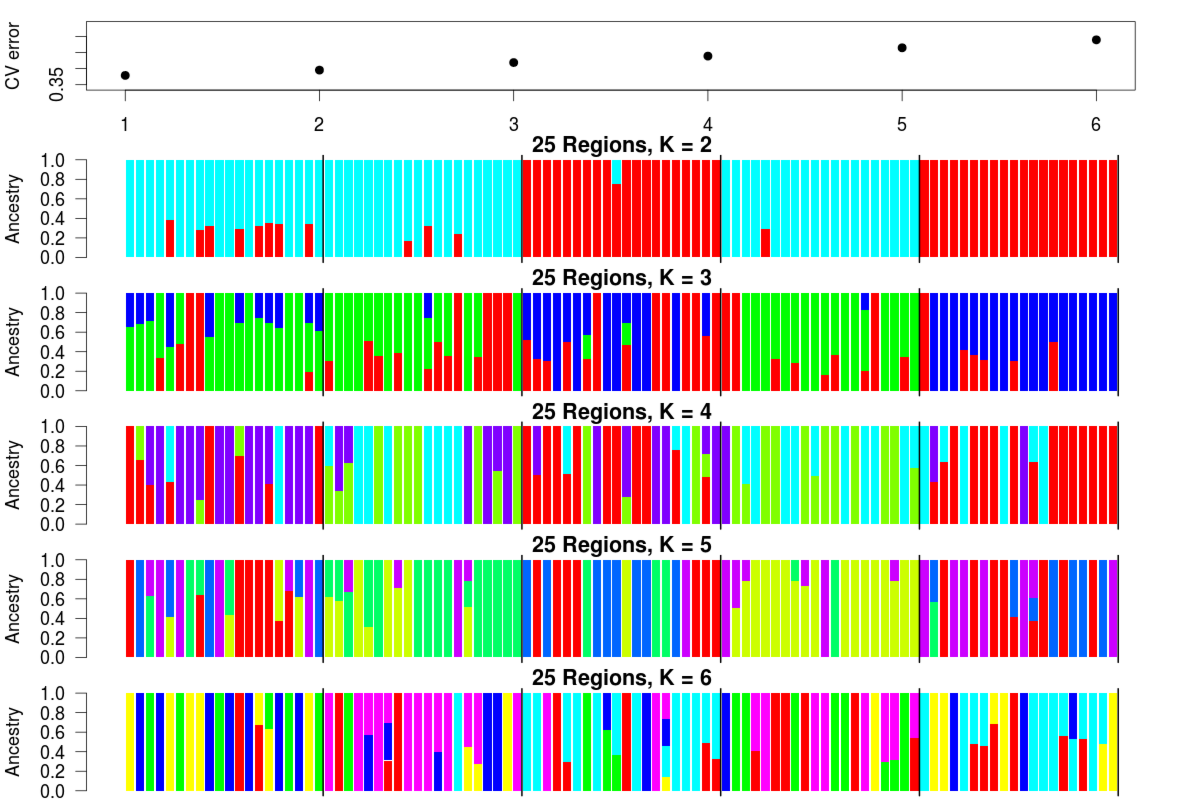

Supplement: Figure S6 — ADMIXTURE results for simulated data at 25 linked regions. Top: cross-validation error (lower is better). True populations are separated by a black line. The maximum correlation with truth is obtained at K = 3. (TIFF) [file pgen.1002453.s006.tiff]

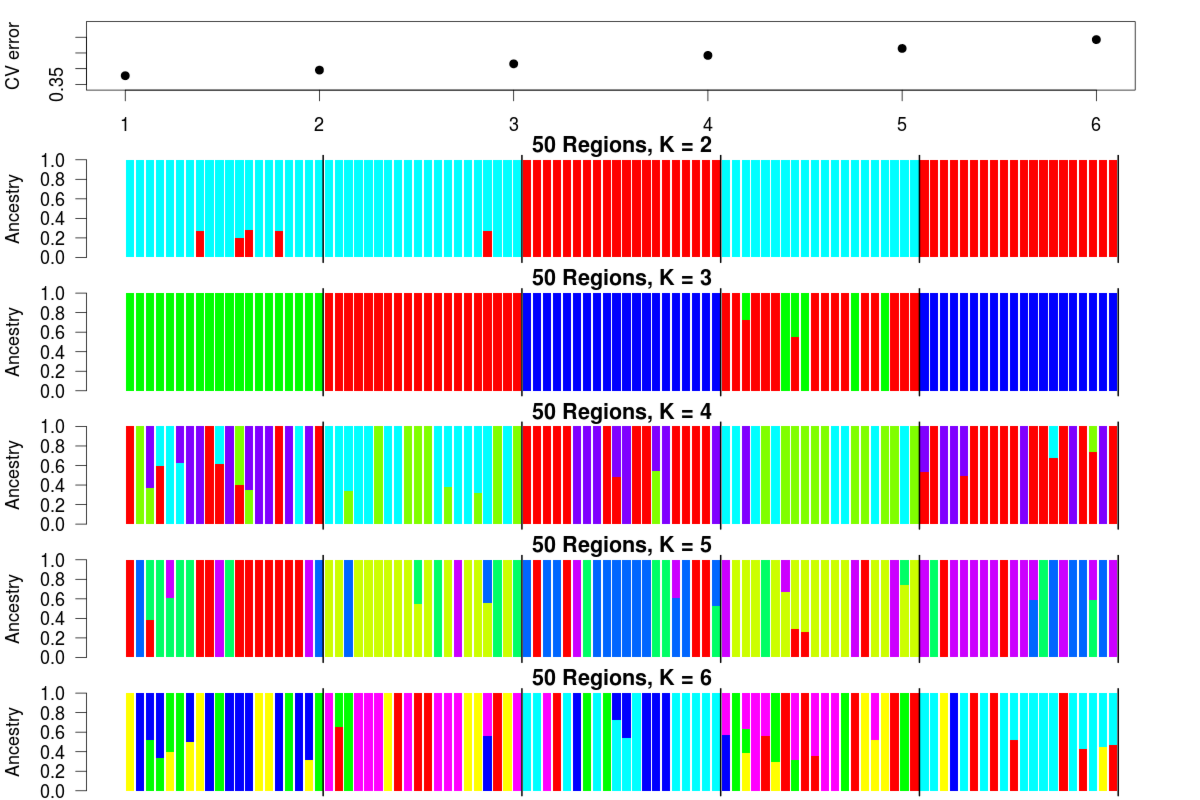

Supplement: Figure S7 — ADMIXTURE results for simulated data at 50 linked regions. Top: cross-validation error (lower is better). True populations are separated by a black line. The maximum correlation with truth is obtained at K = 3. (TIFF) [file pgen.1002453.s007.tiff]

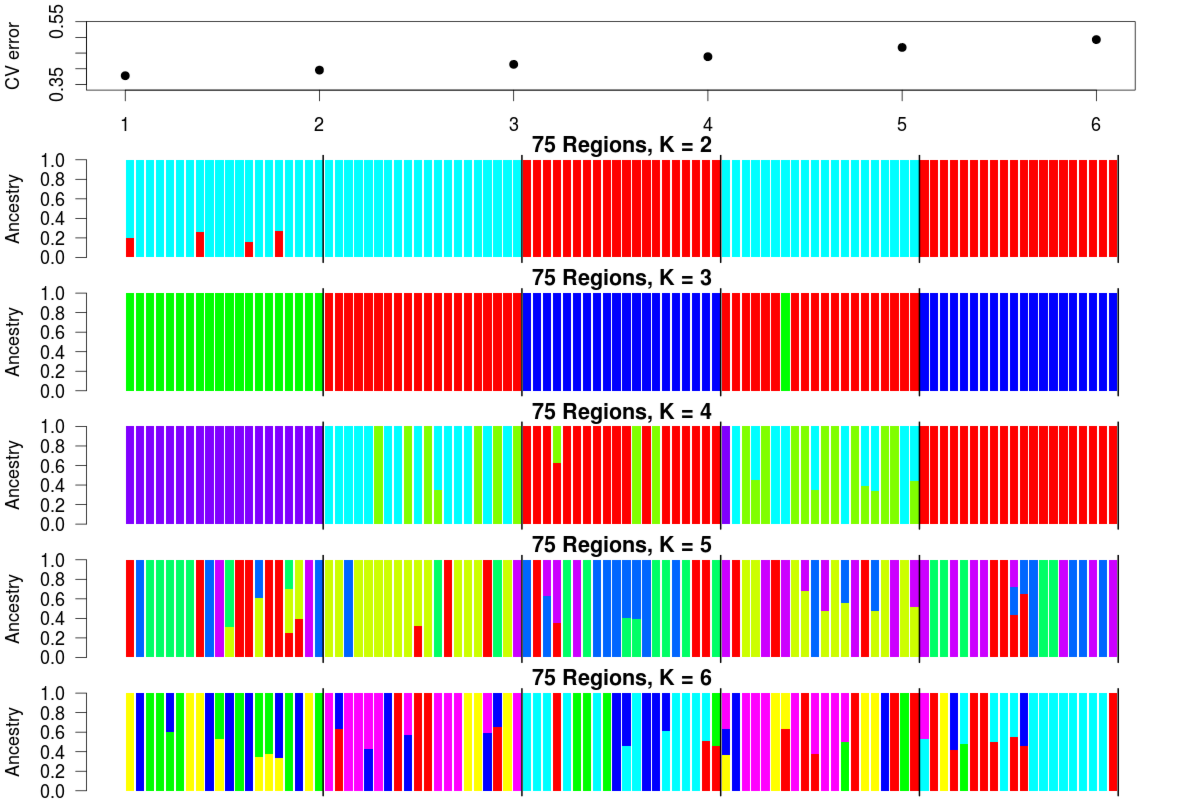

Supplement: Figure S8 — ADMIXTURE results for simulated data at 75 linked regions. Top: cross-validation error (lower is better). True populations are separated by a black line. The maximum correlation with truth is obtained at K = 3. (TIFF) [file pgen.1002453.s008.tiff]

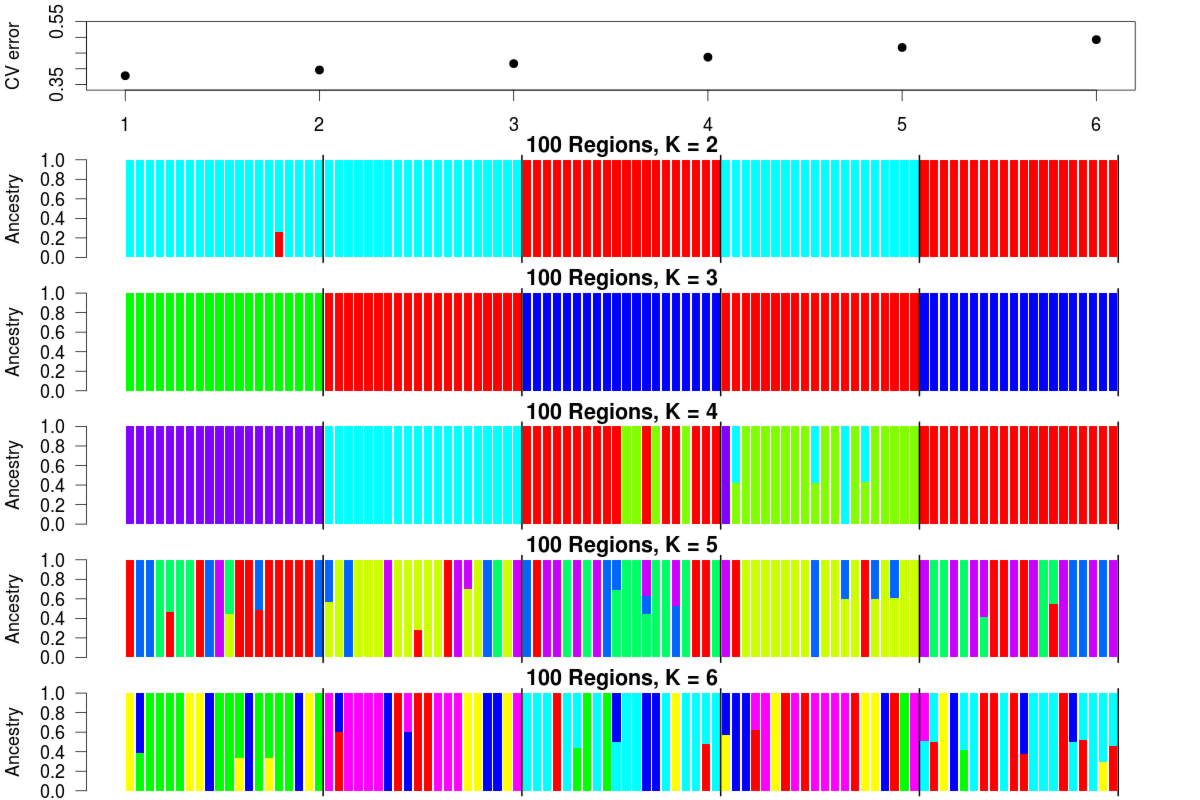

Supplement: Figure S9 — ADMIXTURE results for simulated data at 100 regions. Top: cross-validation error (lower is better). True populations are separated by a black line. The maximum correlation with truth is obtained at K = 4. (TIFF) [file pgen.1002453.s009.tiff]

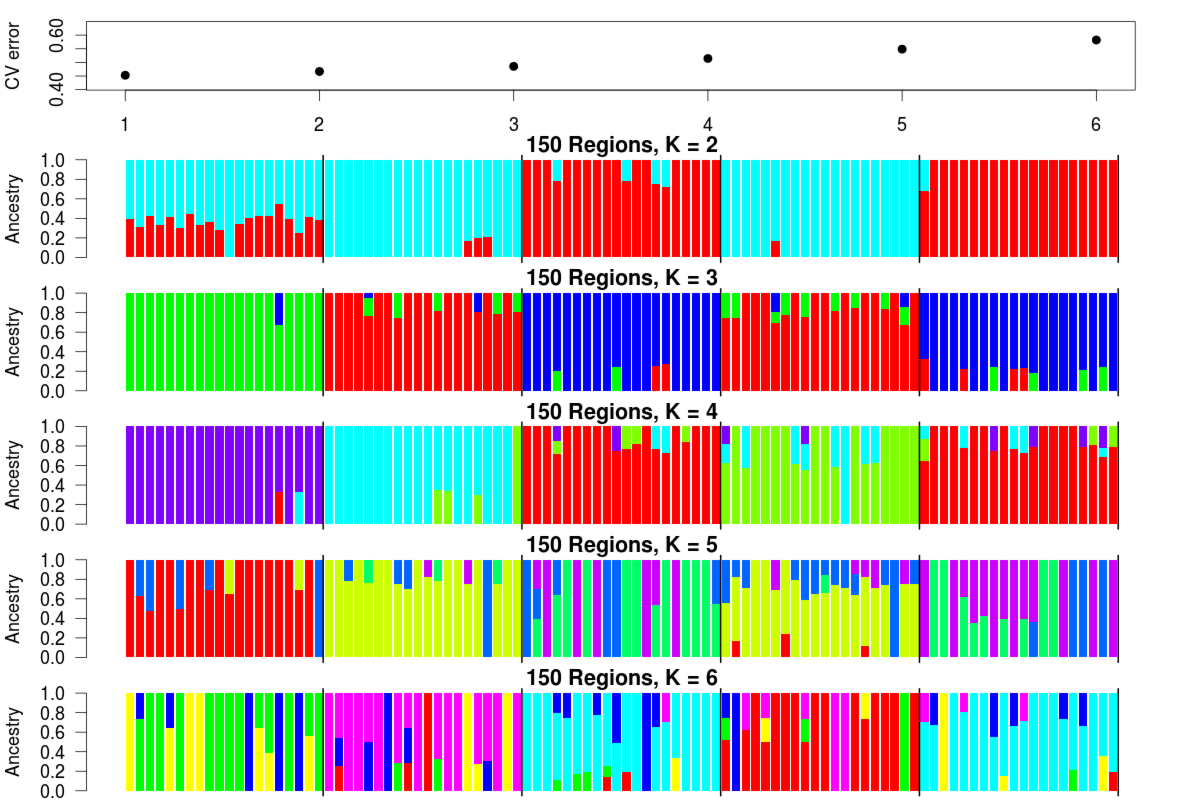

Supplement: Figure S10 — ADMIXTURE results for simulated data at 150 regions. Top: cross-validation error (lower is better). True populations are separated by a black line. The maximum correlation with truth is obtained at K = 4. (TIFF) [file pgen.1002453.s010.tiff]

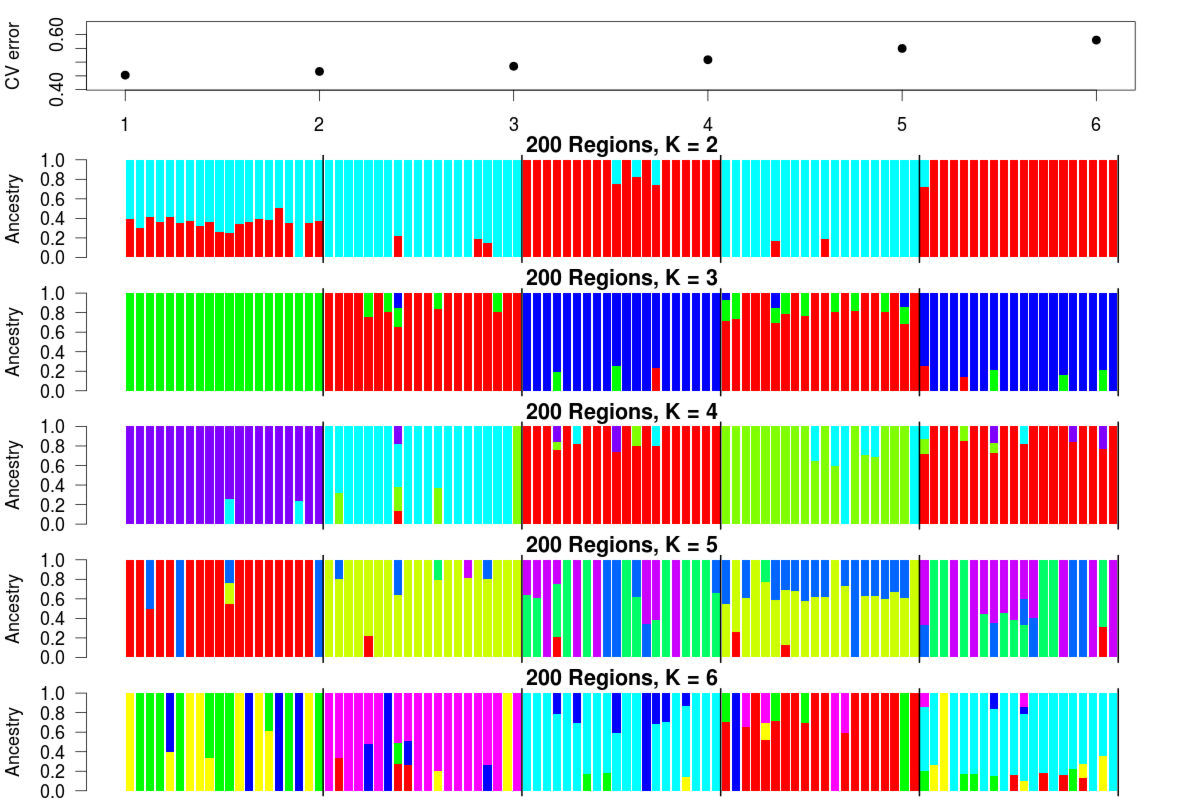

Supplement: Figure S11 — ADMIXTURE results for simulated data at 200 regions. Top: cross-validation error (lower is better). True populations are separated by a black line. The maximum correlation with truth is obtained at K = 4. (TIFF) [file pgen.1002453.s011.tiff]

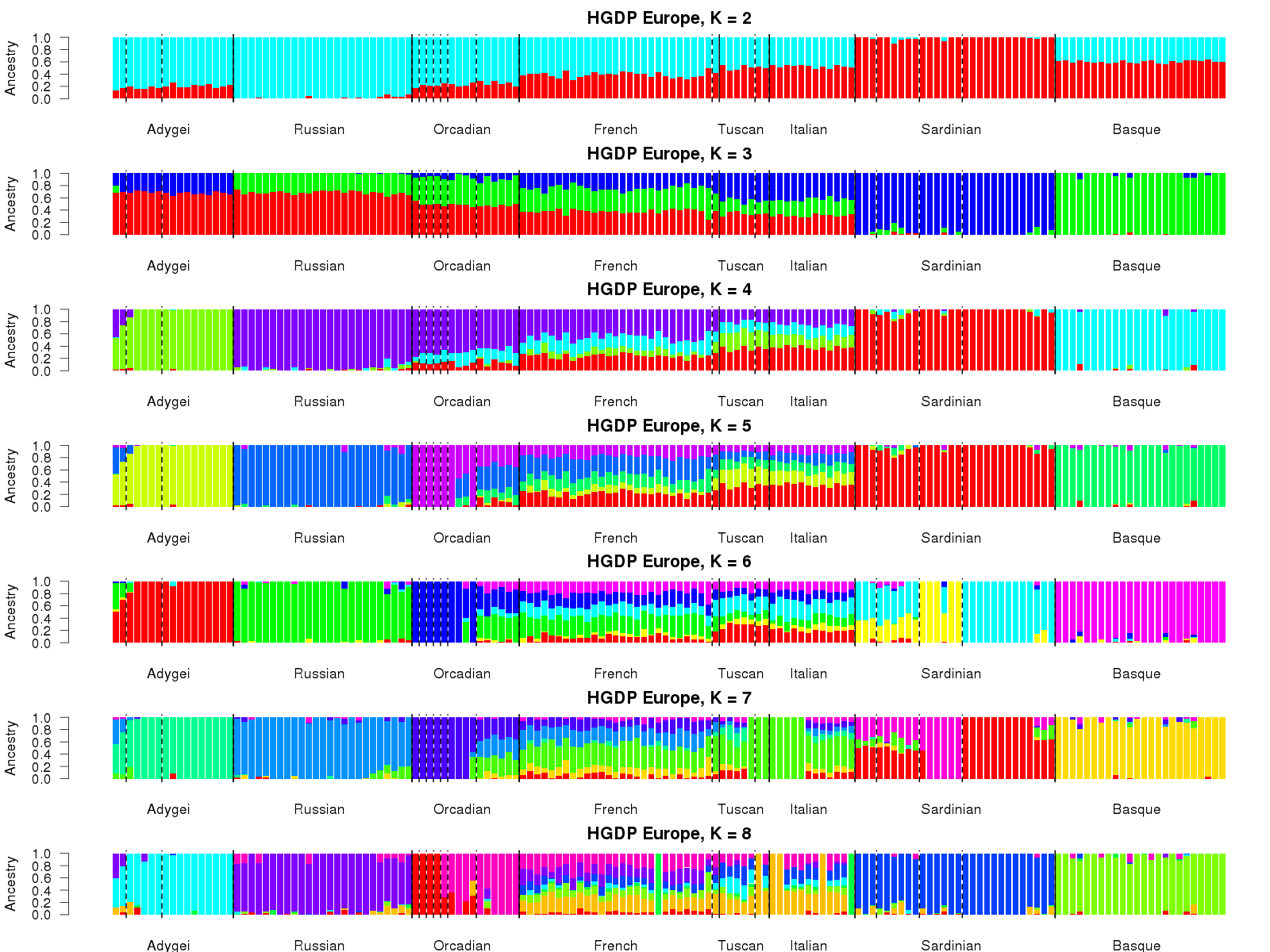

Supplement: Figure S12 — ADMIXTURE results for the HGDP Europe dataset. A range of K is considered as described in the text. Dashed lines separate fineSTRUCTURE populations, solid lines separate labelled populations. fineSTRUCTURE agrees with all labelled populations with the exception of the Tuscan/French. (TIFF) [file pgen.1002453.s012.tiff]

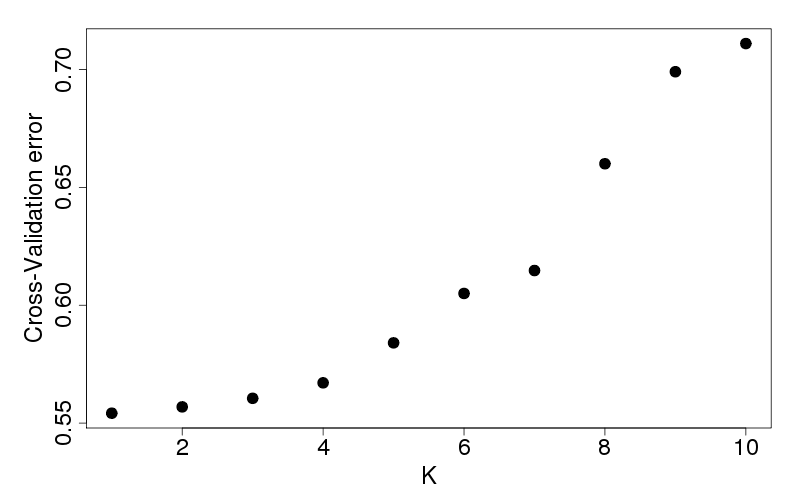

Supplement: Figure S13 — ADMIXTURE cross validation error as a function of . The recommended procedure is to choose the with the minimum cross-validation error, here . (TIFF) [file pgen.1002453.s013.tiff]

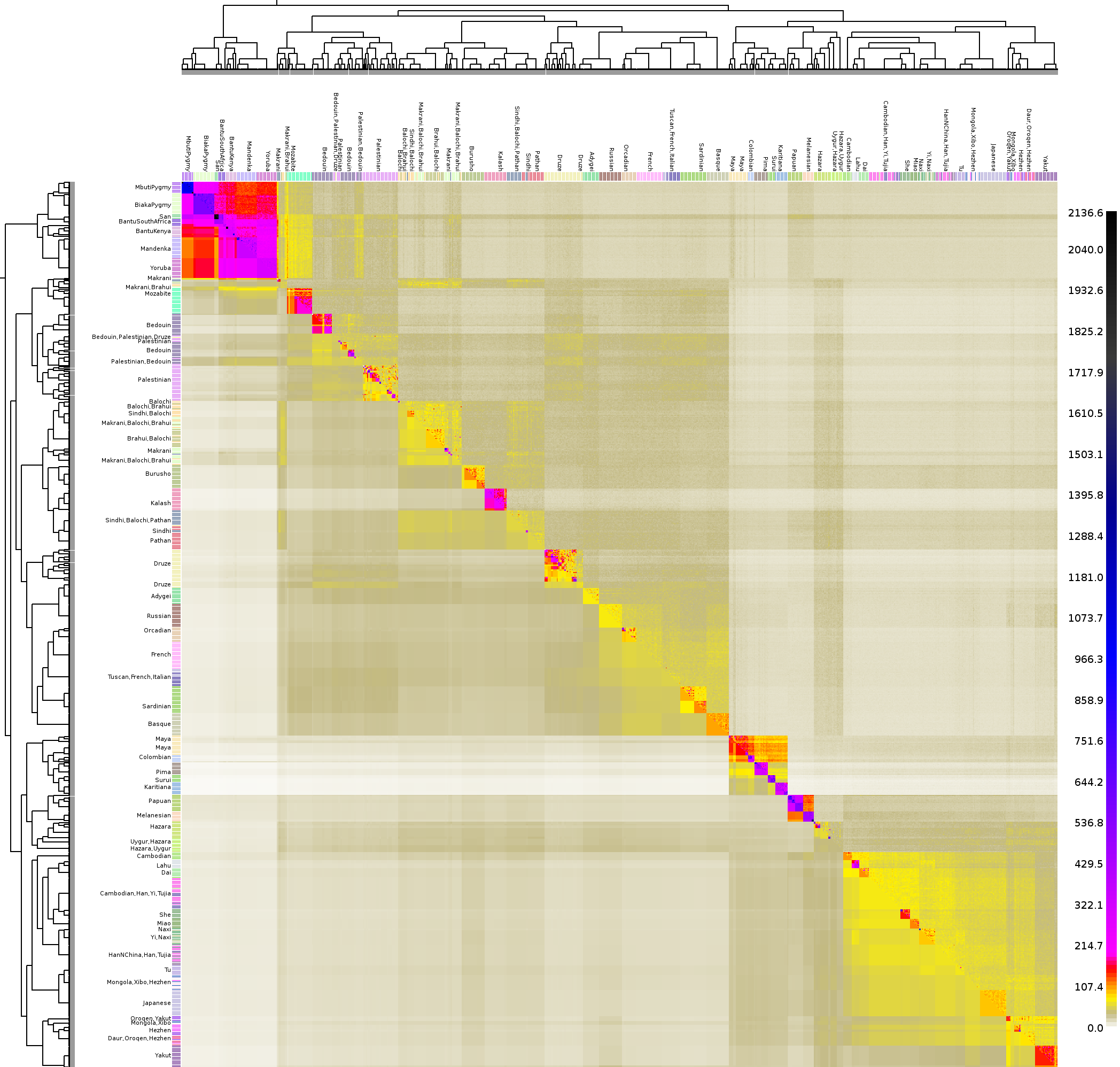

Supplement: Figure S14 — Whole world HGDP coancestry matrix. Some population labels are omitted for clarity; this has only been done when the neighbouring population contains the same labels and the exact distribution is recoverable from the tree and Figure 4 of the main text. The colour scale is non-linear, and population sizes have been square-rooted for clarity. (TIFF) [file pgen.1002453.s014.tiff]

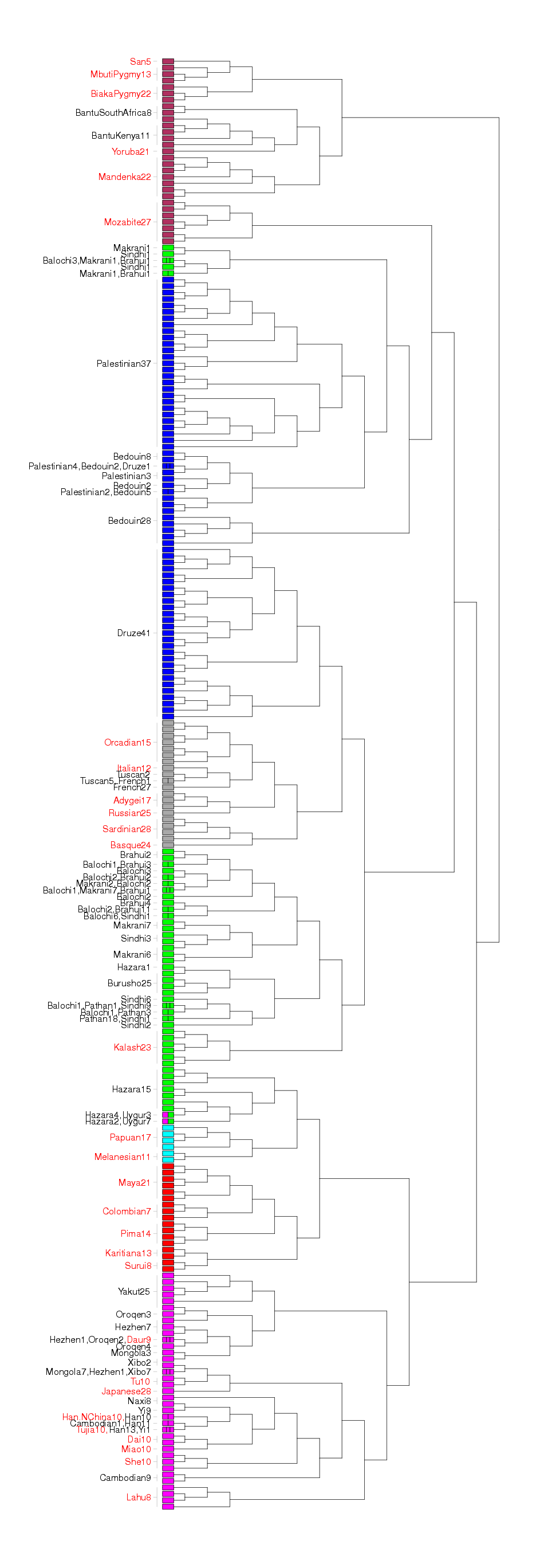

Supplement: Figure S15 — “Sub-continental” tree for all HGDP populations. Inference was performed in separate subcontinents groupings as defined in Figure 4 of the main text, with details for each subcontinent given in Figures S16, S17, S18, S19, S20, S21, S22, S23, S24.The interpretation is the same as Figure 4 of the main text (except that probabilities have been removed for clarity). (TIFF) [file pgen.1002453.s015.tiff]

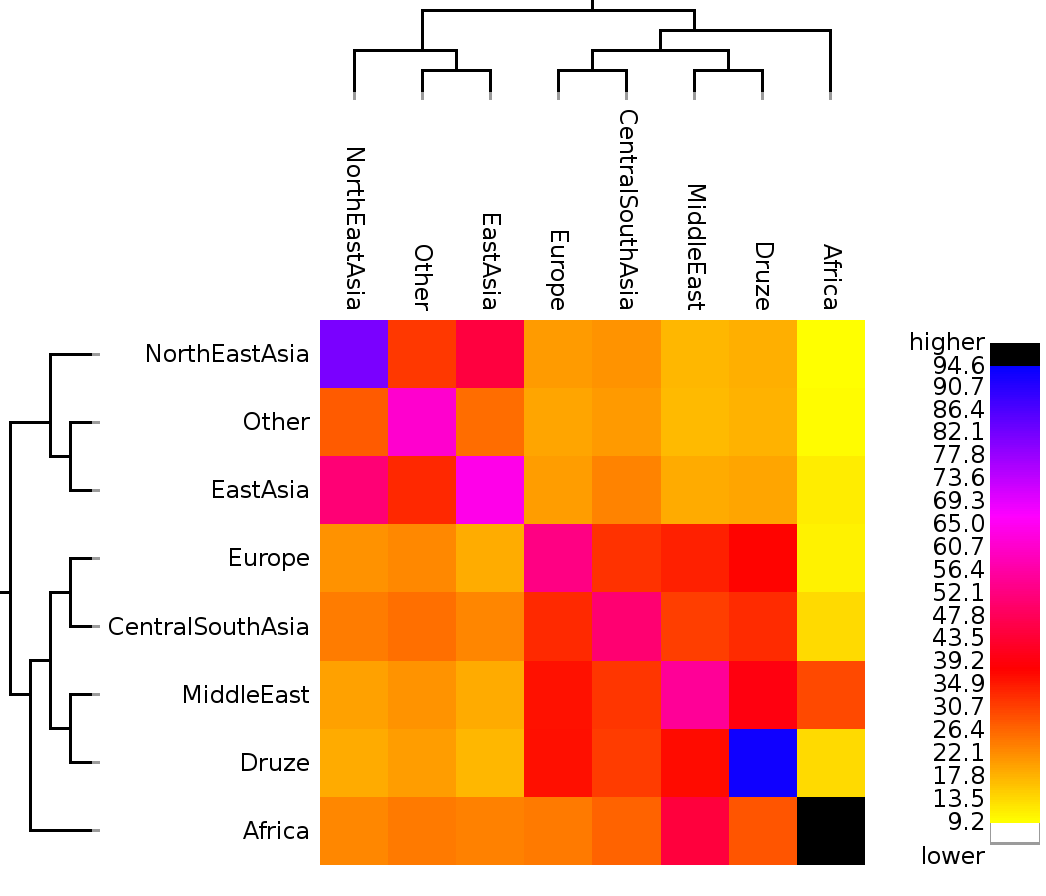

Supplement: Figure S16 — “Sub-continental” coancestry matrix. Groupings as defined in Figure 4 of the main text. Recipient groups are on the left. Note that Africa has been capped, and copies 232 chunks to itself. (TIFF) [file pgen.1002453.s016.tiff]

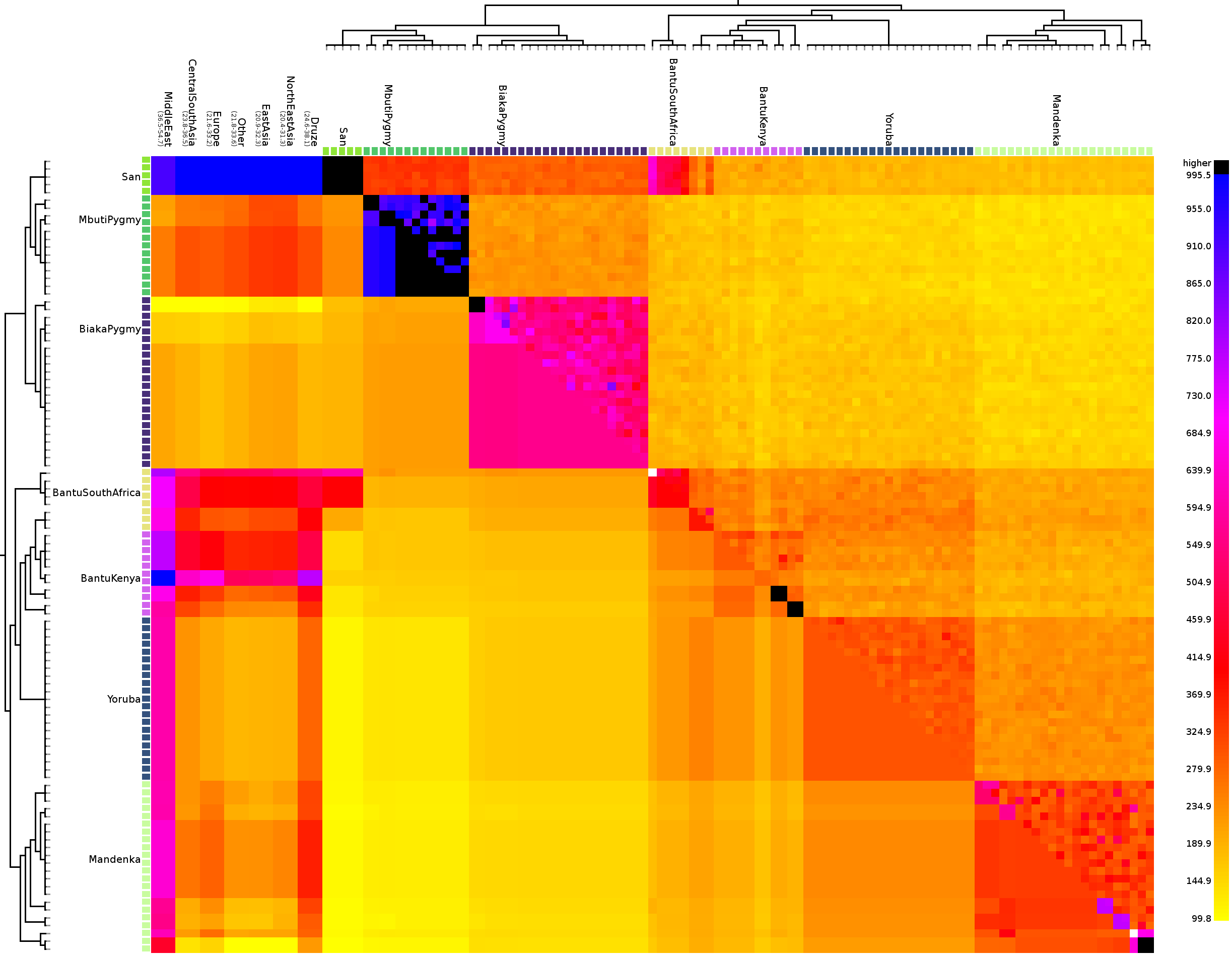

Supplement: Figure S17 — “Sub-continent” of Africa coancestry matrix. (bottom left) the Population coancestry matrix and (top right) the Individual coancestry matrix. (TIFF) [file pgen.1002453.s017.tiff]

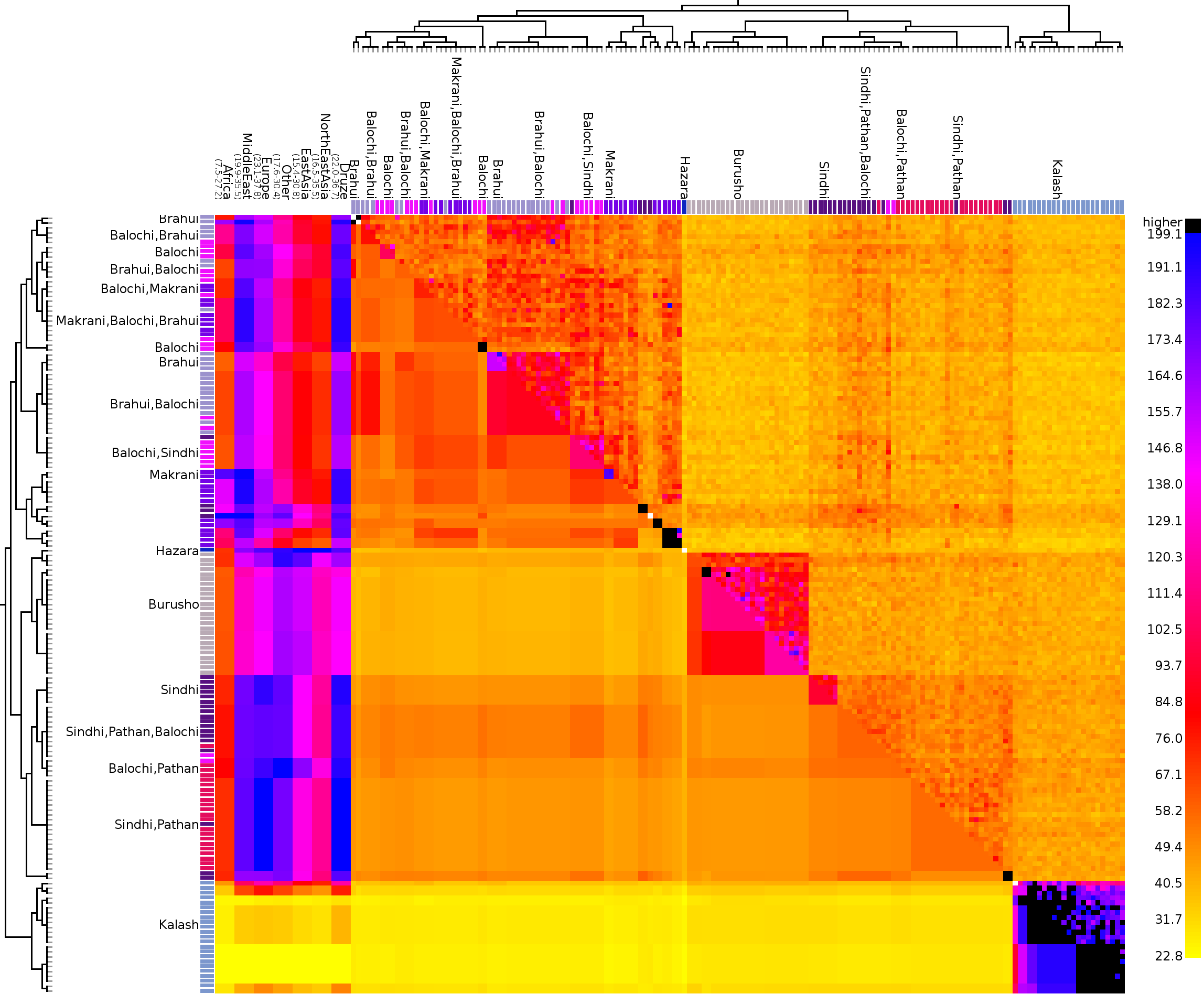

Supplement: Figure S18 — “Sub-continent” of CentralSouthAsia coancestry matrix. (bottom left) the Population coancestry matrix and (top right) the Individual coancestry matrix. (TIFF) [file pgen.1002453.s018.tiff]

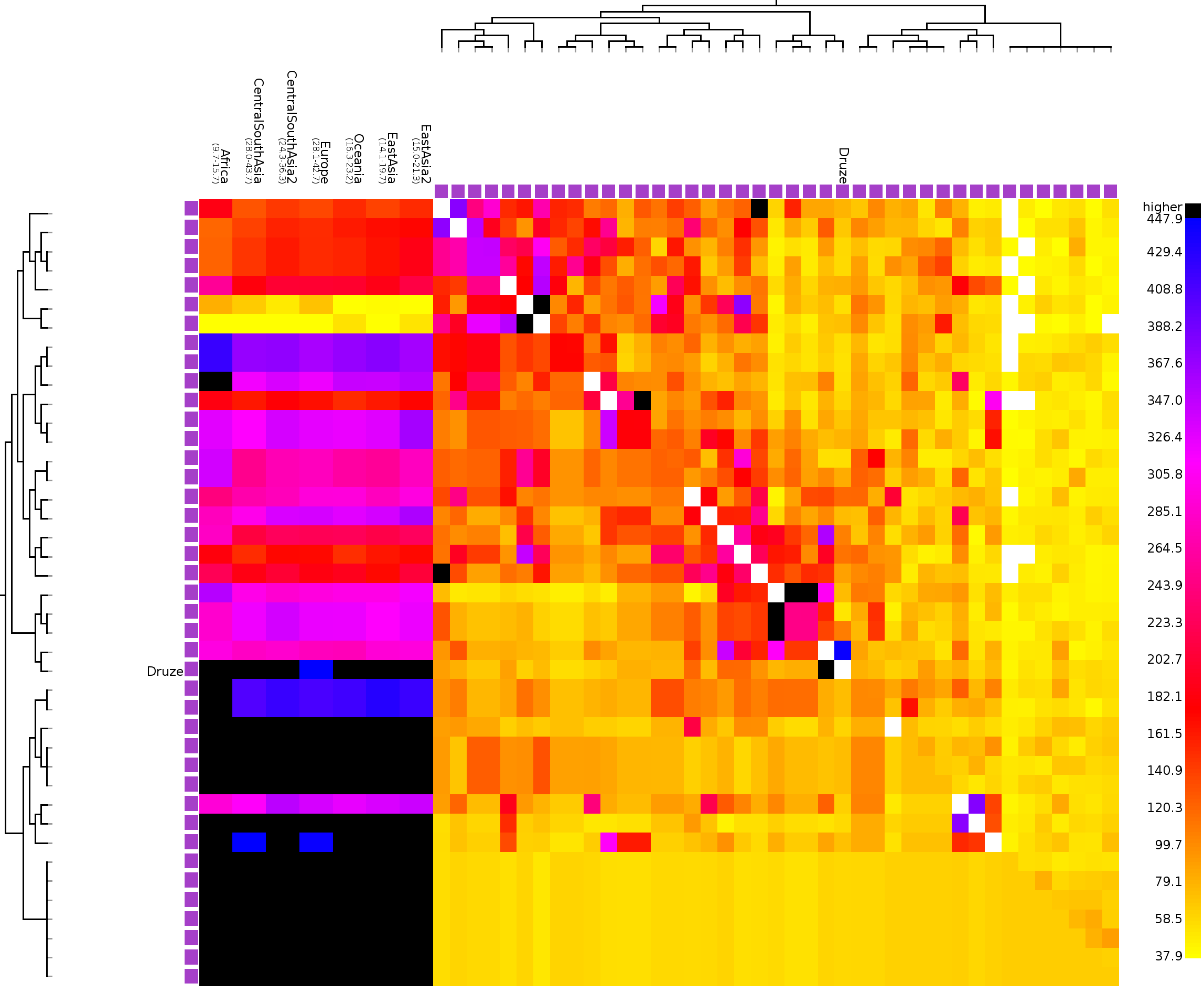

Supplement: Figure S19 — “Sub-continent” of Druze coancestry matrix. (bottom left) the Population coancestry matrix and (top right) the Individual coancestry matrix. (TIFF) [file pgen.1002453.s019.tiff]

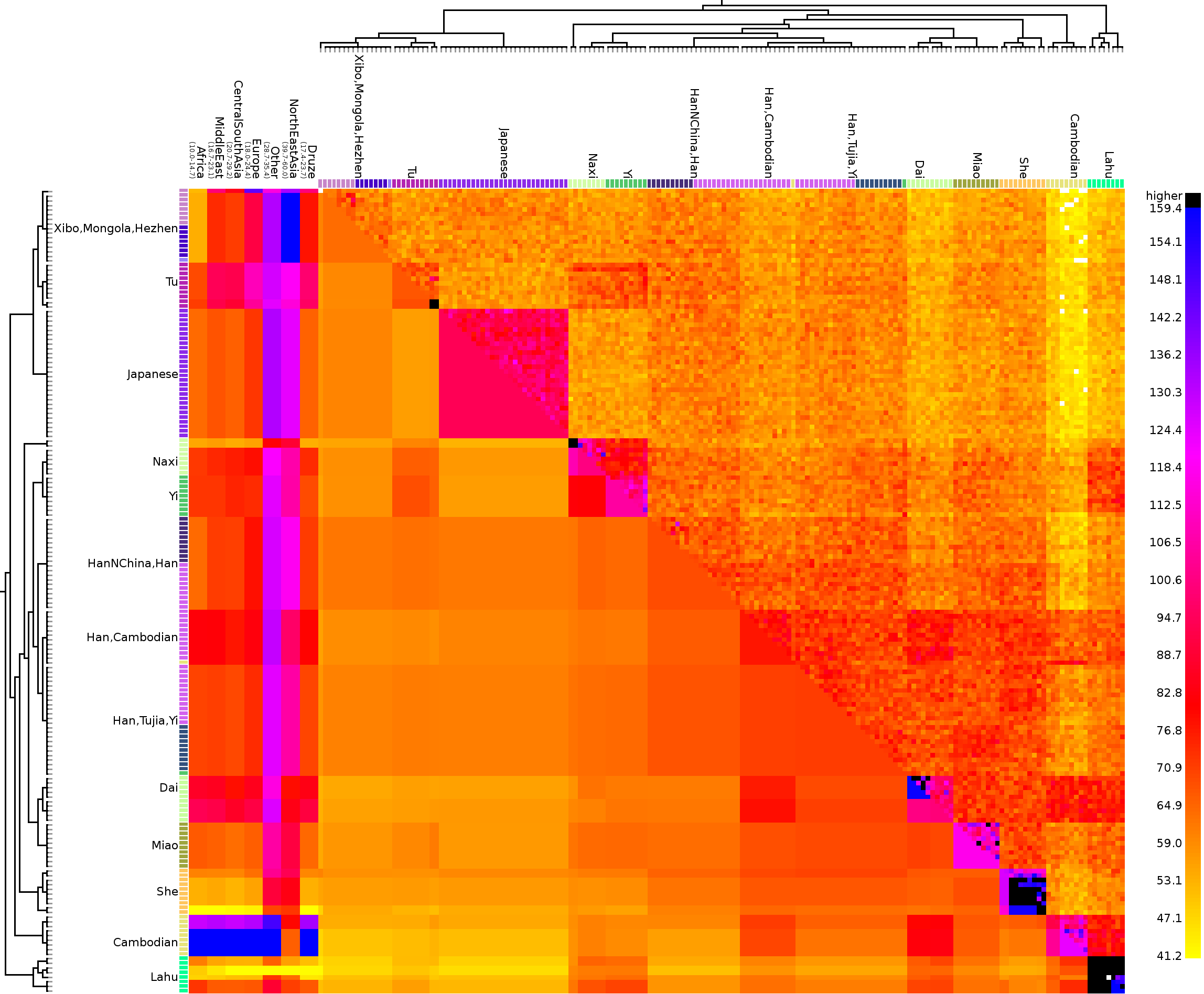

Supplement: Figure S20 — “Sub-continent” of EastAsia coancestry matrix. (bottom left) the Population coancestry matrix and (top right) the Individual coancestry matrix. (TIFF) [file pgen.1002453.s020.tiff]

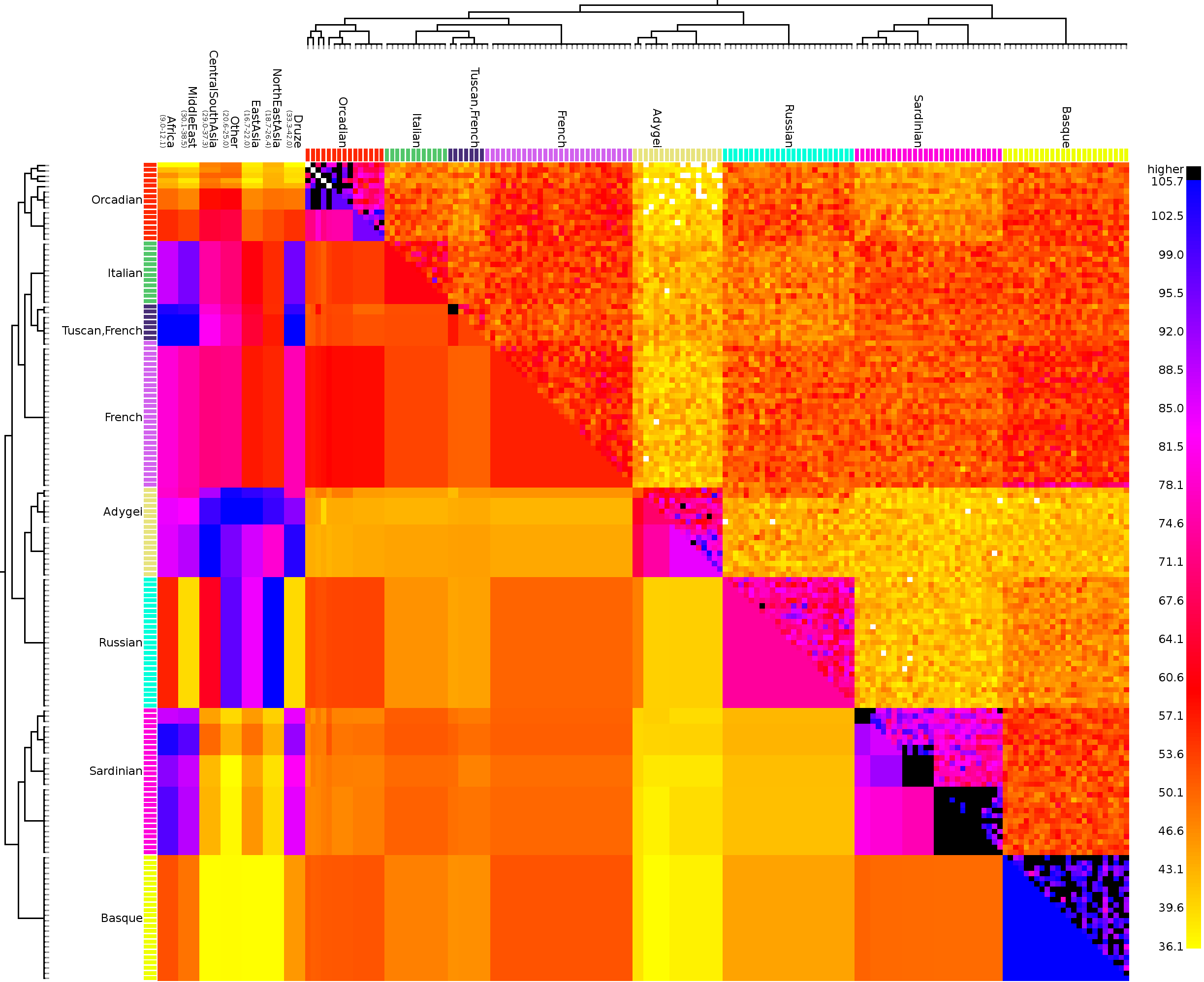

Supplement: Figure S21 — “Sub-continent” of Europe coancestry matrix. (bottom left) the Population coancestry matrix and (top right) the Individual coancestry matrix. (TIFF) [file pgen.1002453.s021.tiff]

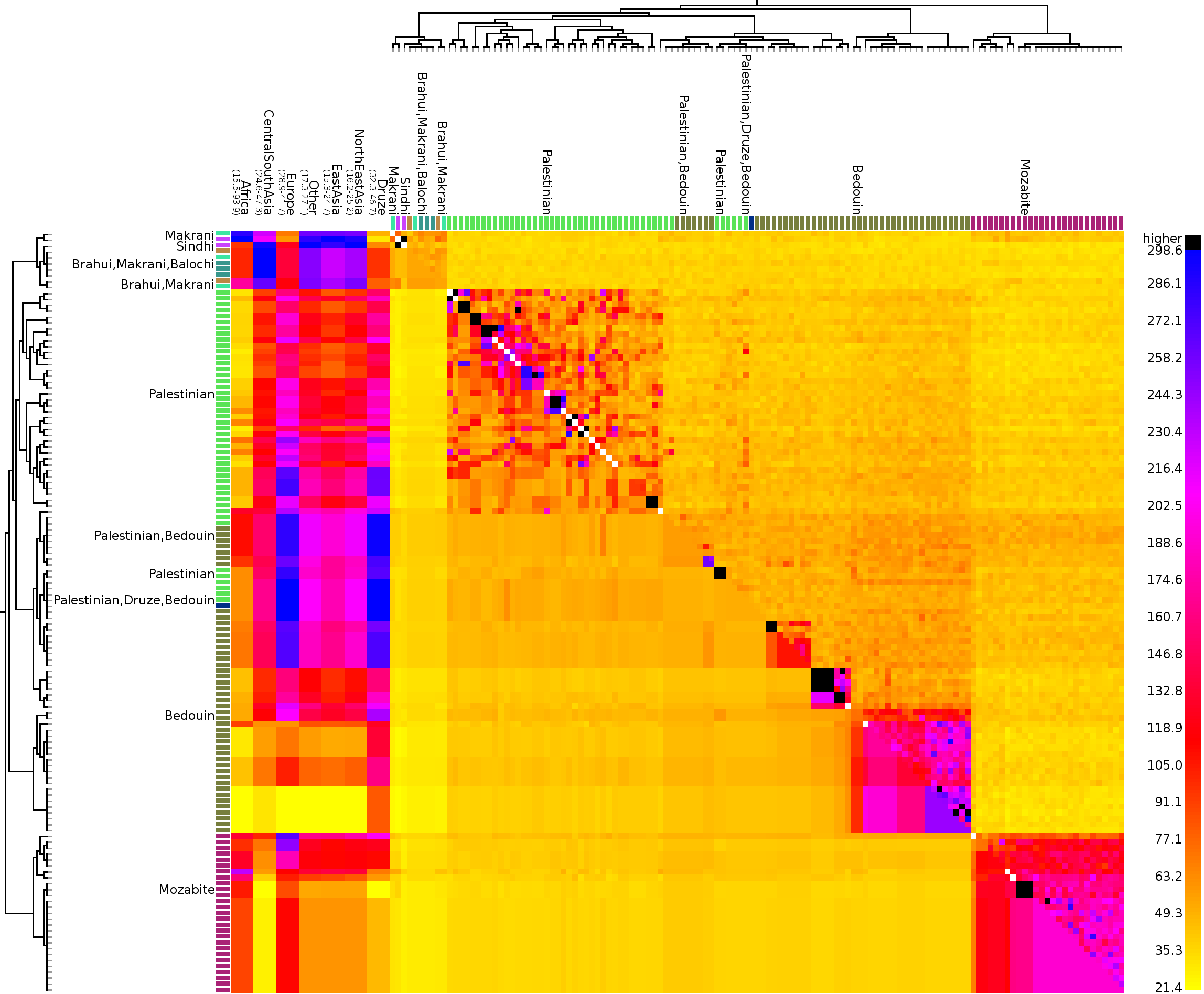

Supplement: Figure S22 — “Sub-continent” of MiddleEast coancestry matrix. (bottom left) the Population coancestry matrix and (top right) the Individual coancestry matrix. (TIFF) [file pgen.1002453.s022.tiff]

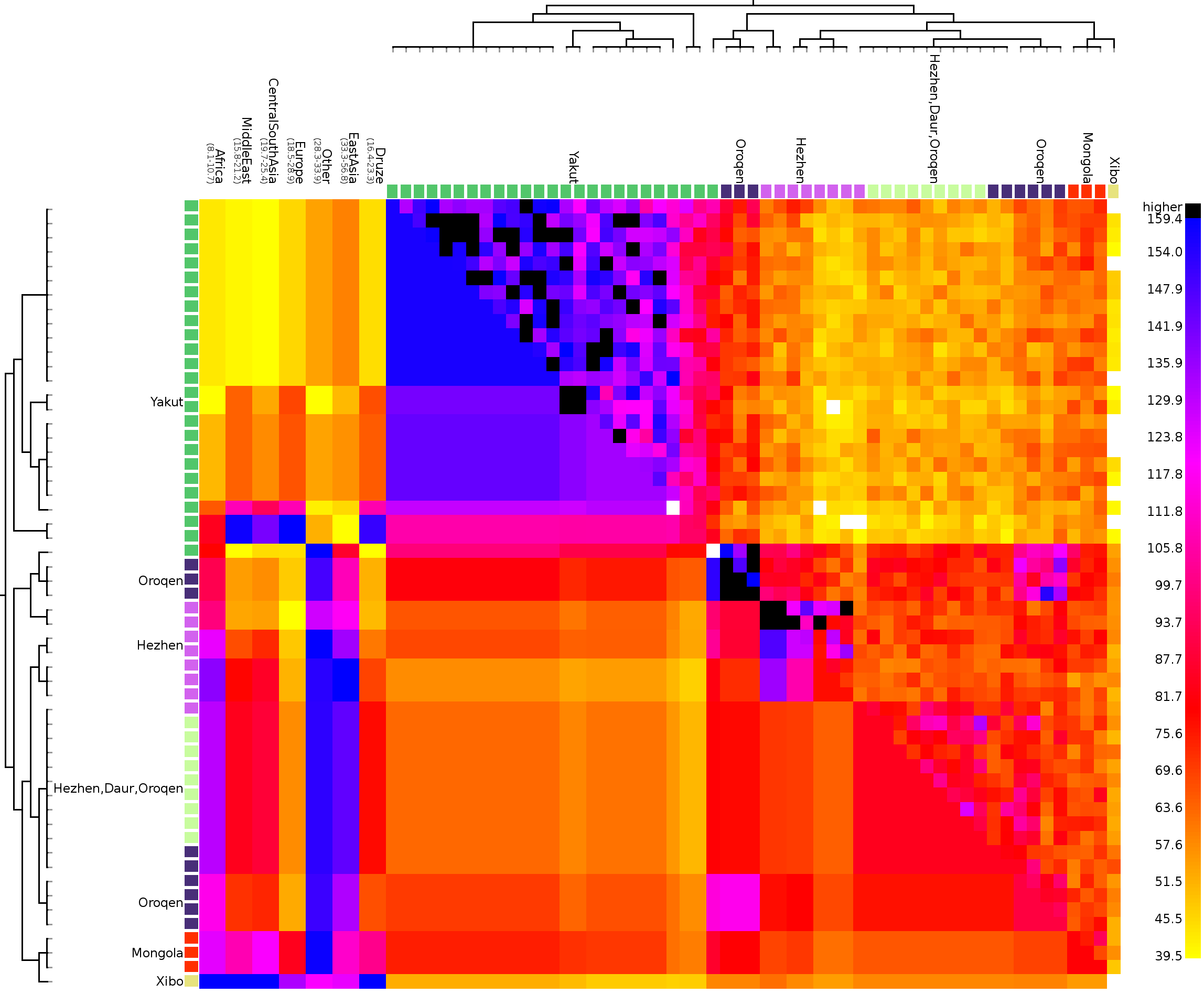

Supplement: Figure S23 — “Sub-continent” of NorthEastAsia coancestry matrix. (bottom left) the Population coancestry matrix and (top right) the Individual coancestry matrix. (TIFF) [file pgen.1002453.s023.tiff]

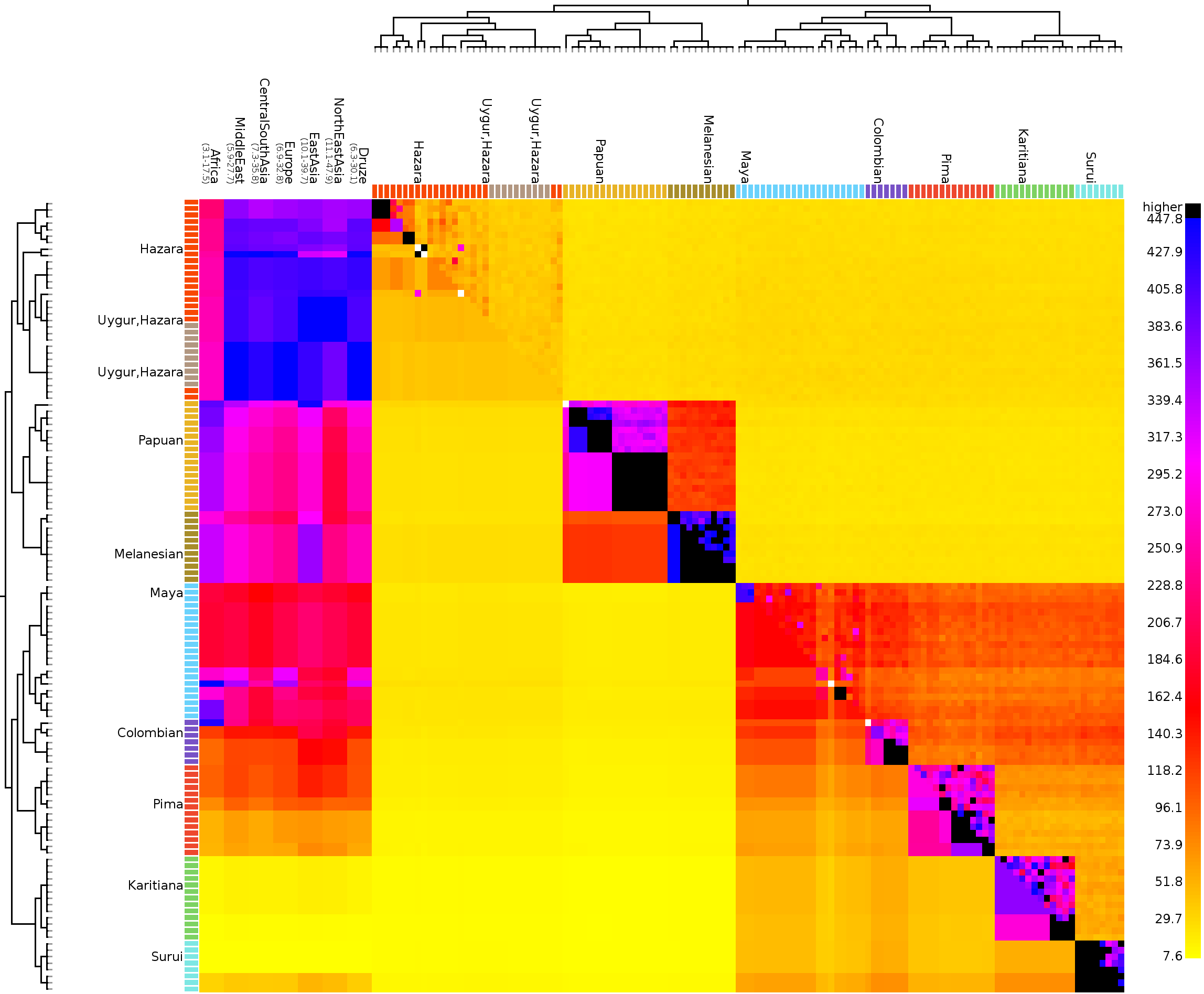

Supplement: Figure S24 — “Sub-continent” of “Other” populations. ‘Other’ is defined as America, Oceania and some Asian individuals. (bottom left) the Population coancestry matrix and (top right) the Individual coancestry matrix. (TIFF) [file pgen.1002453.s024.tiff]

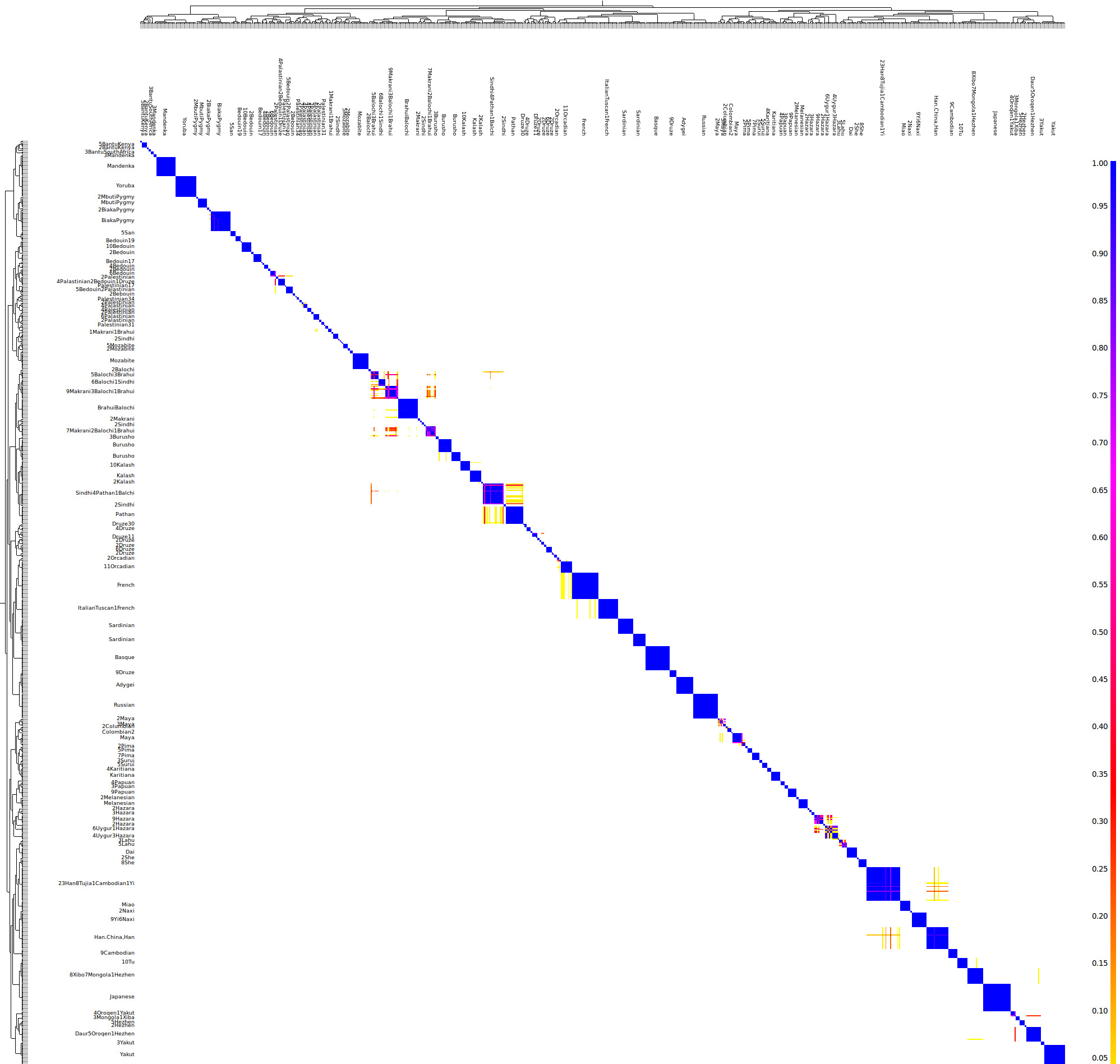

Supplement: Figure S25 — Whole HGDP pairwise coincidence matrix. (bottom left) run 1 and (top right) an independent run 2. It is recommended to view this figure online and use zoom tools. (TIFF) [file pgen.1002453.s025.tiff]

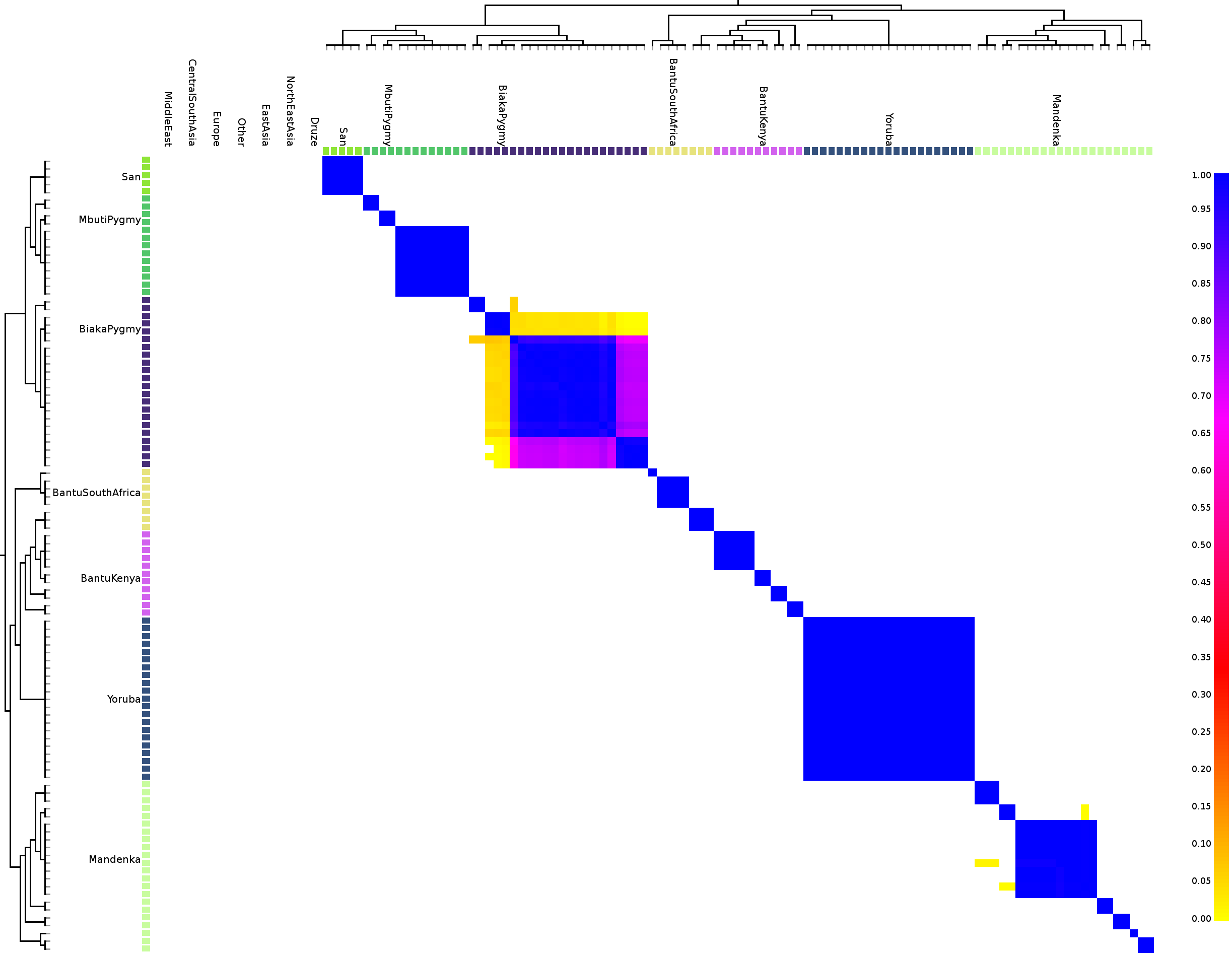

Supplement: Figure S26 — Africa pairwise coincidence matrix. (bottom left) run 1 and (top right) independent run 2. (TIFF) [file pgen.1002453.s026.tiff]

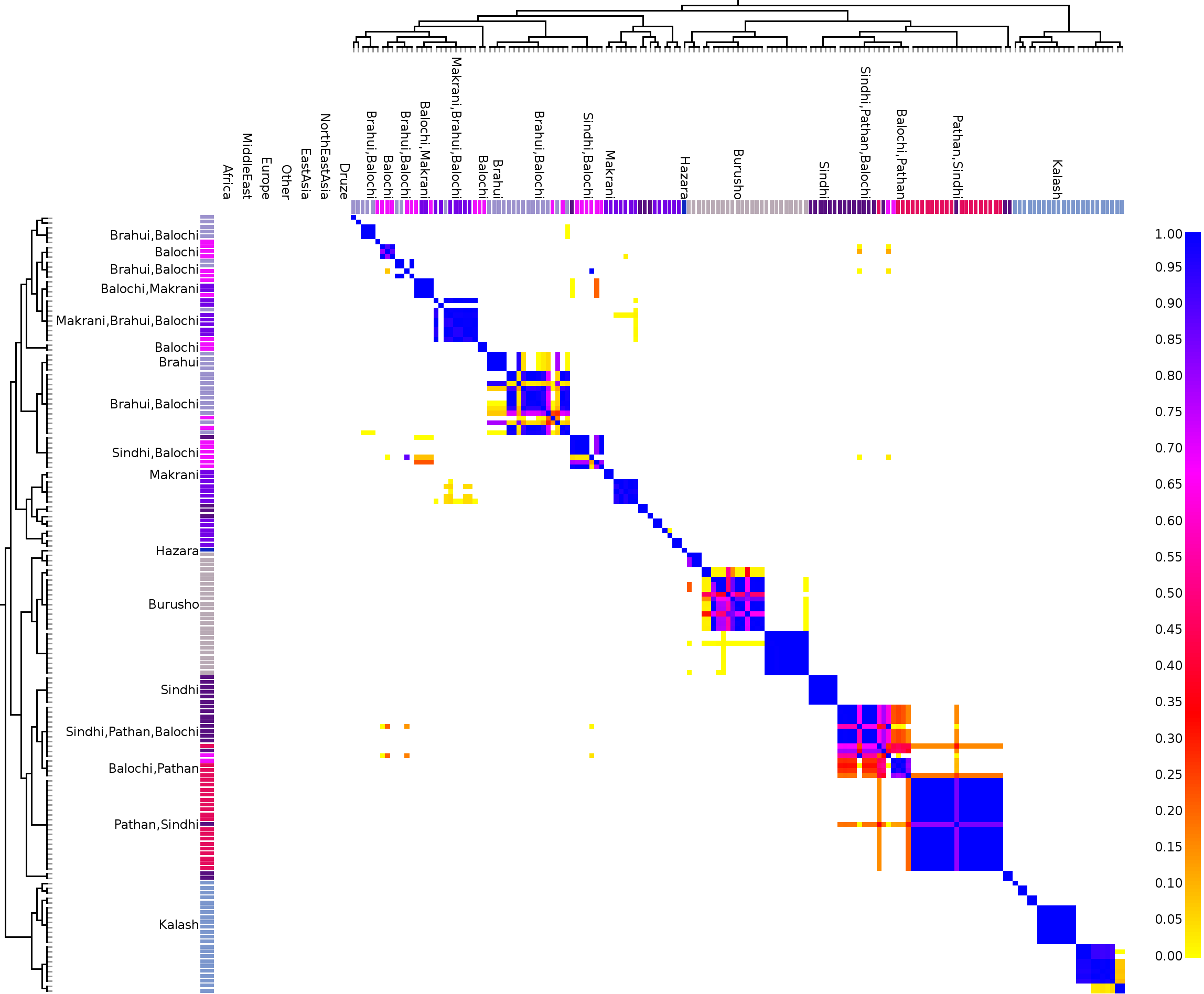

Supplement: Figure S27 — CentralSouthAsia pairwise coincidence matrix. (bottom left) run 1 and (top right) independent run 2. (TIFF) [file pgen.1002453.s027.tiff]

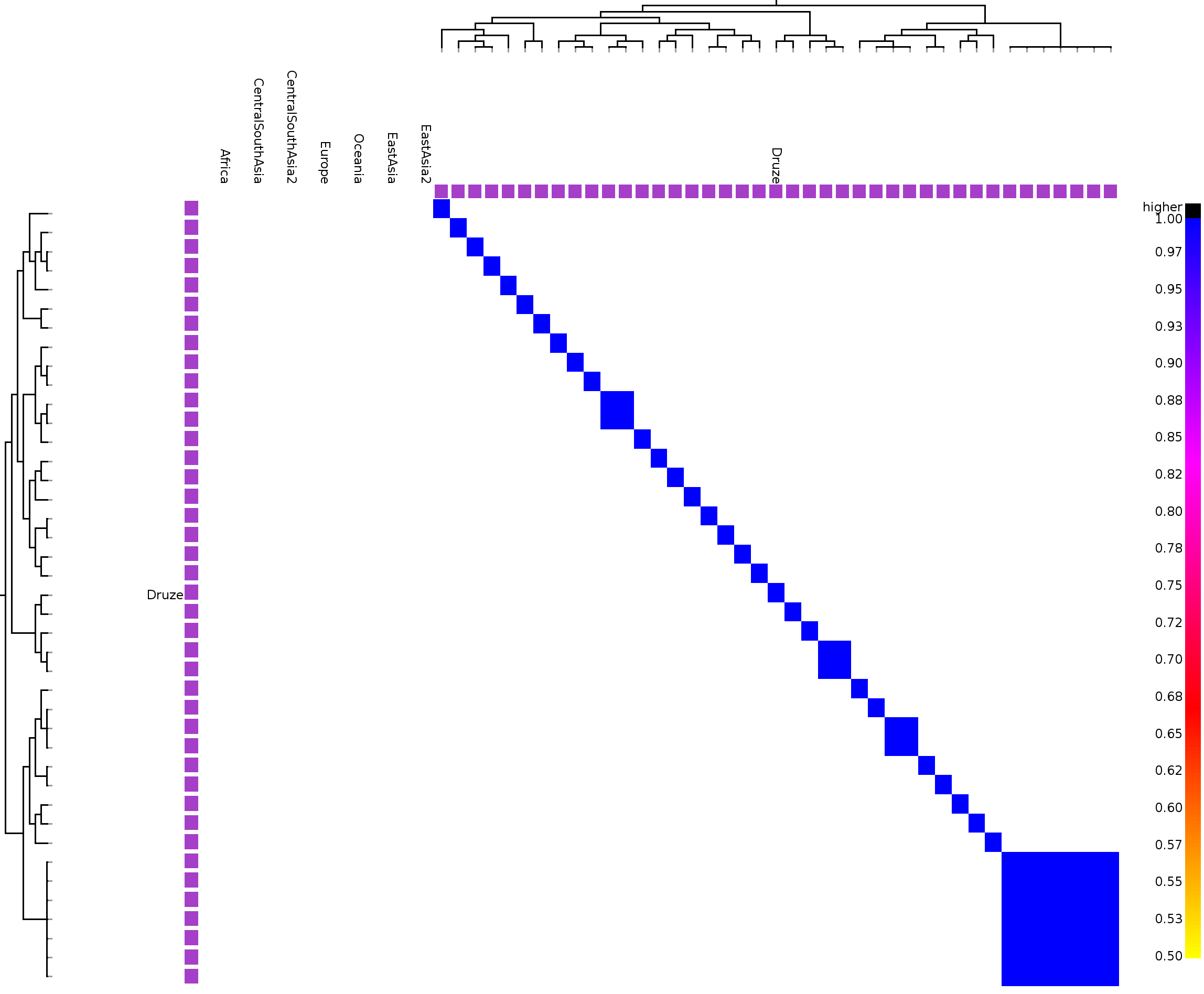

Supplement: Figure S28 — Druze pairwise coincidence matrix. (bottom left) run 1 and (top right) independent run 2. (TIFF) [file pgen.1002453.s028.tiff]

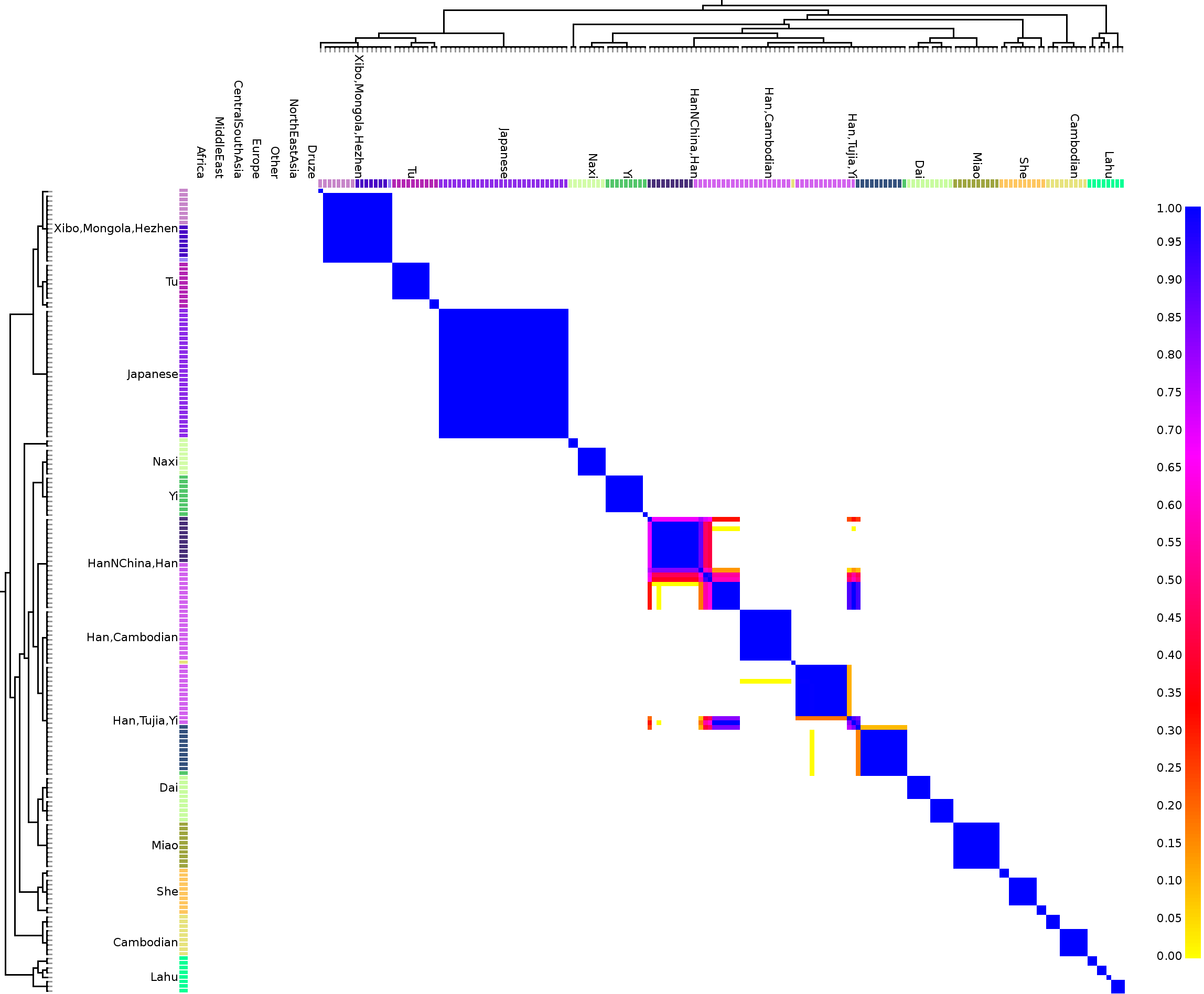

Supplement: Figure S29 — EastAsia pairwise coincidence matrix. (bottom left) run 1 and (top right) independent run 2. (TIFF) [file pgen.1002453.s029.tiff]

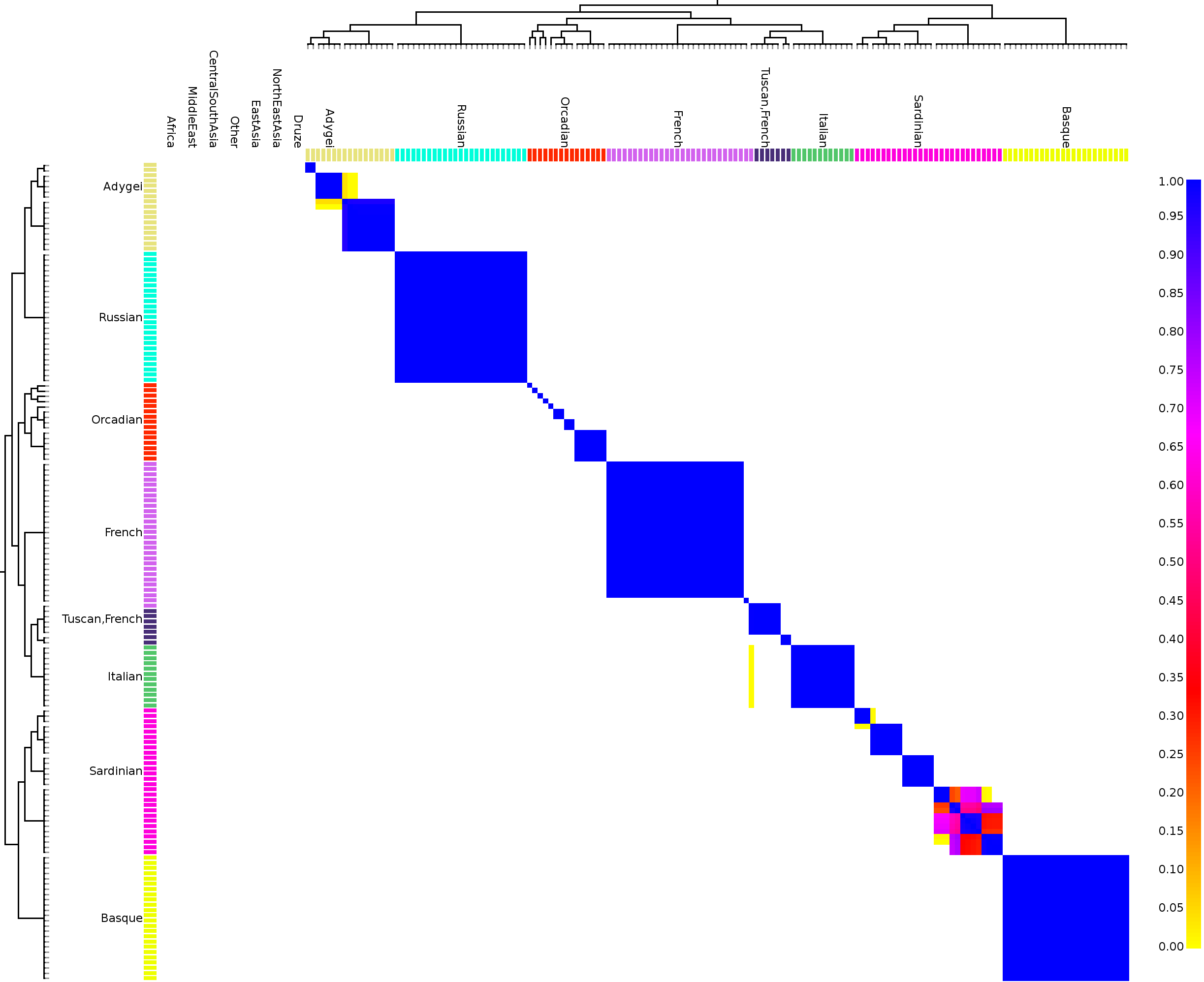

Supplement: Figure S30 — Europe pairwise coincidence matrix. (bottom left) run 1 and (top right) independent run 2. (TIFF) [file pgen.1002453.s030.tiff]

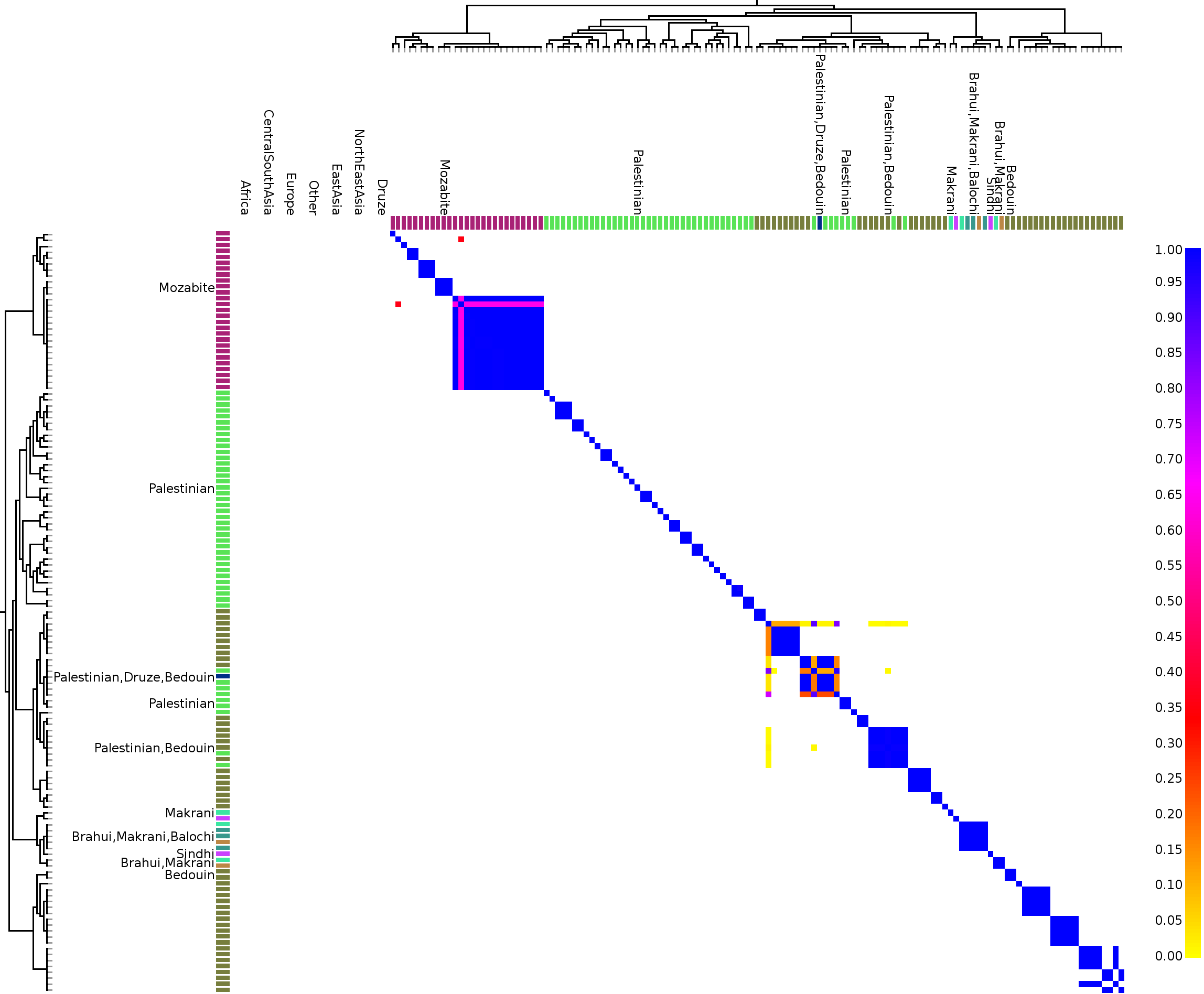

Supplement: Figure S31 — MiddleEast pairwise coincidence matrix. (bottom left) run 1 and (top right) independent run 2. (TIFF) [file pgen.1002453.s031.tiff]

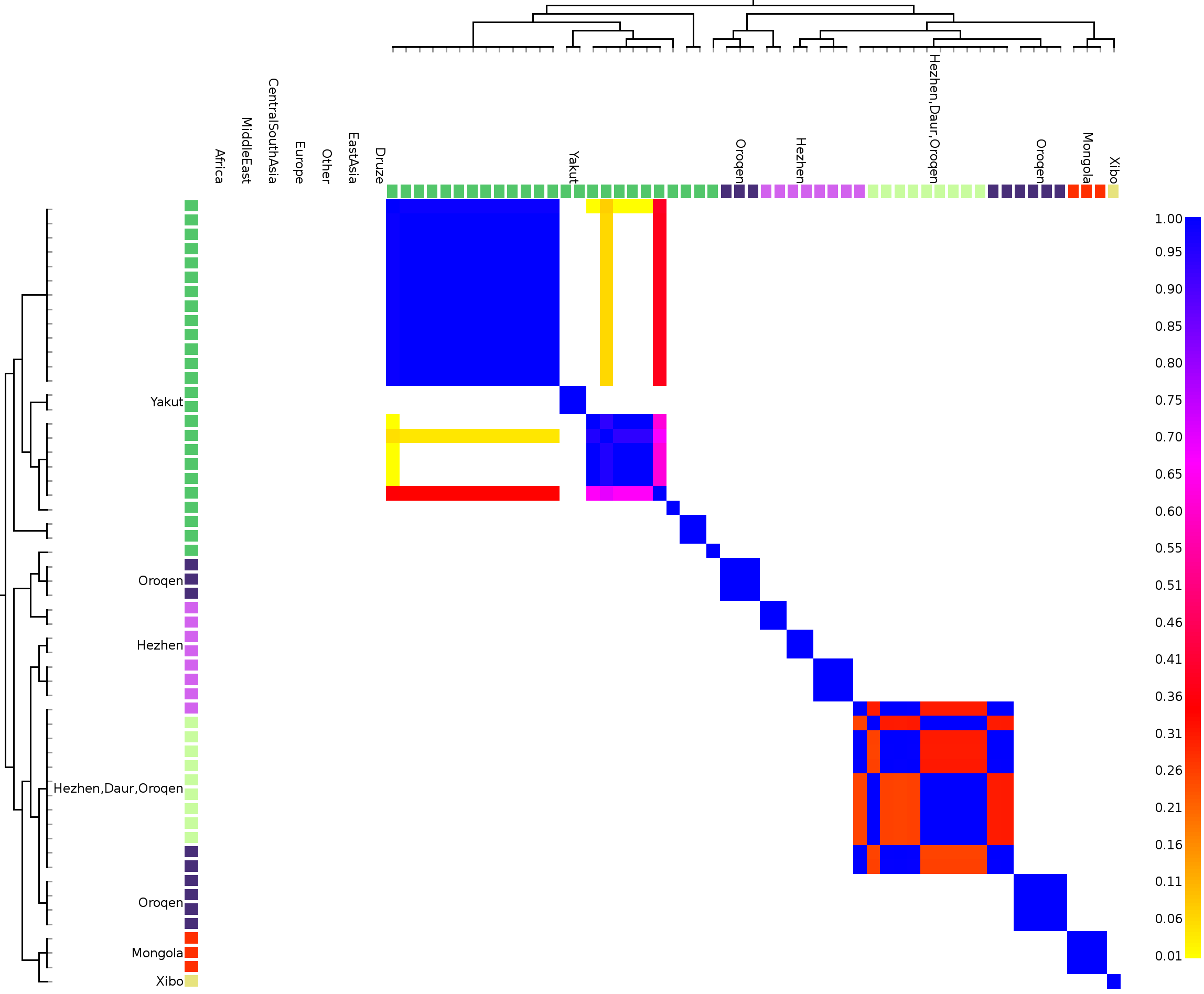

Supplement: Figure S32 — NorthEastAsia pairwise coincidence matrix. (bottom left) run 1 and (top right) independent run 2. (TIFF) [file pgen.1002453.s032.tiff]

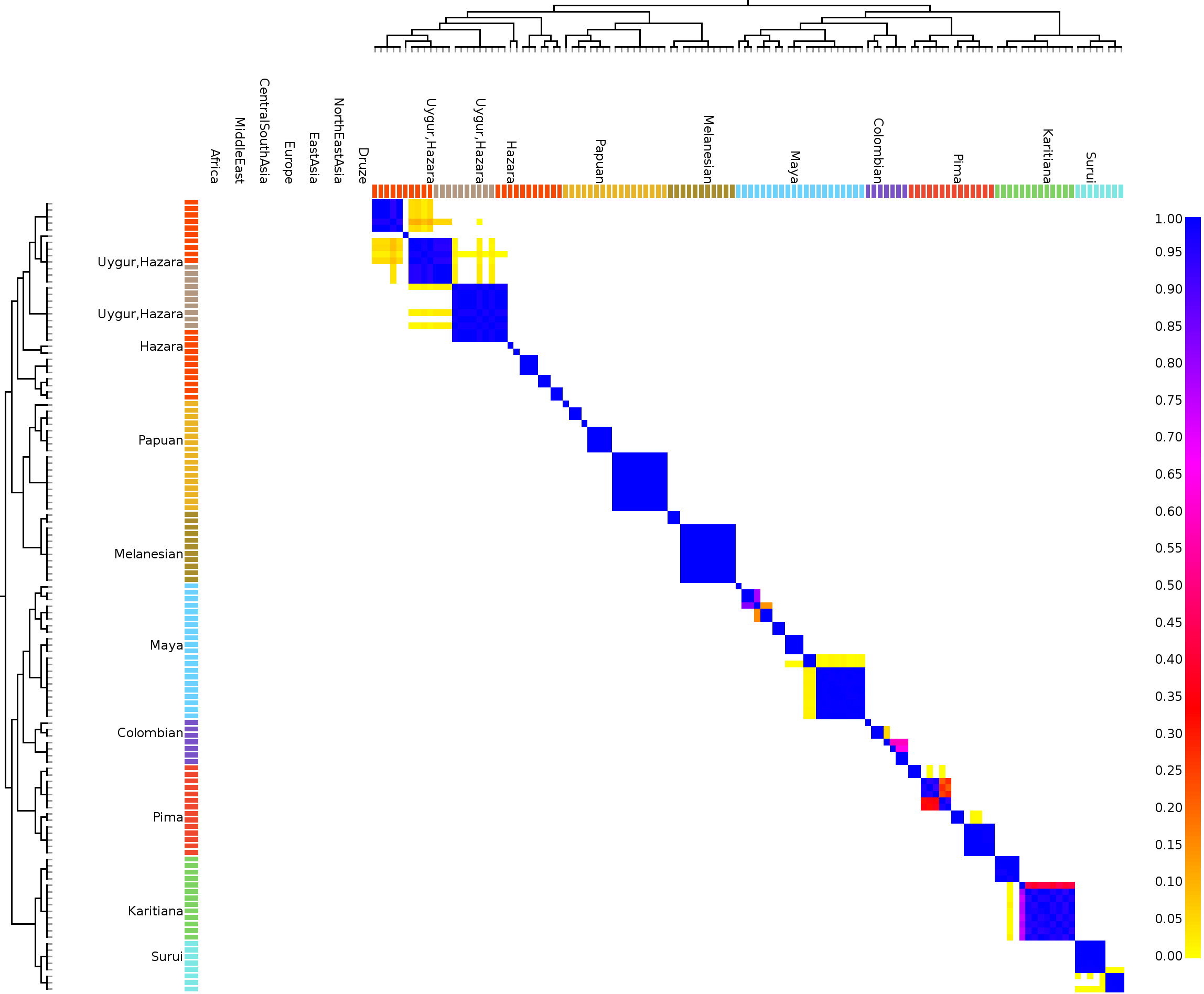

Supplement: Figure S33 — “Other” populations pairwise coincidence matrix. (bottom left) run 1 and (top right) independent run 2. (TIFF) [file pgen.1002453.s033.tiff]

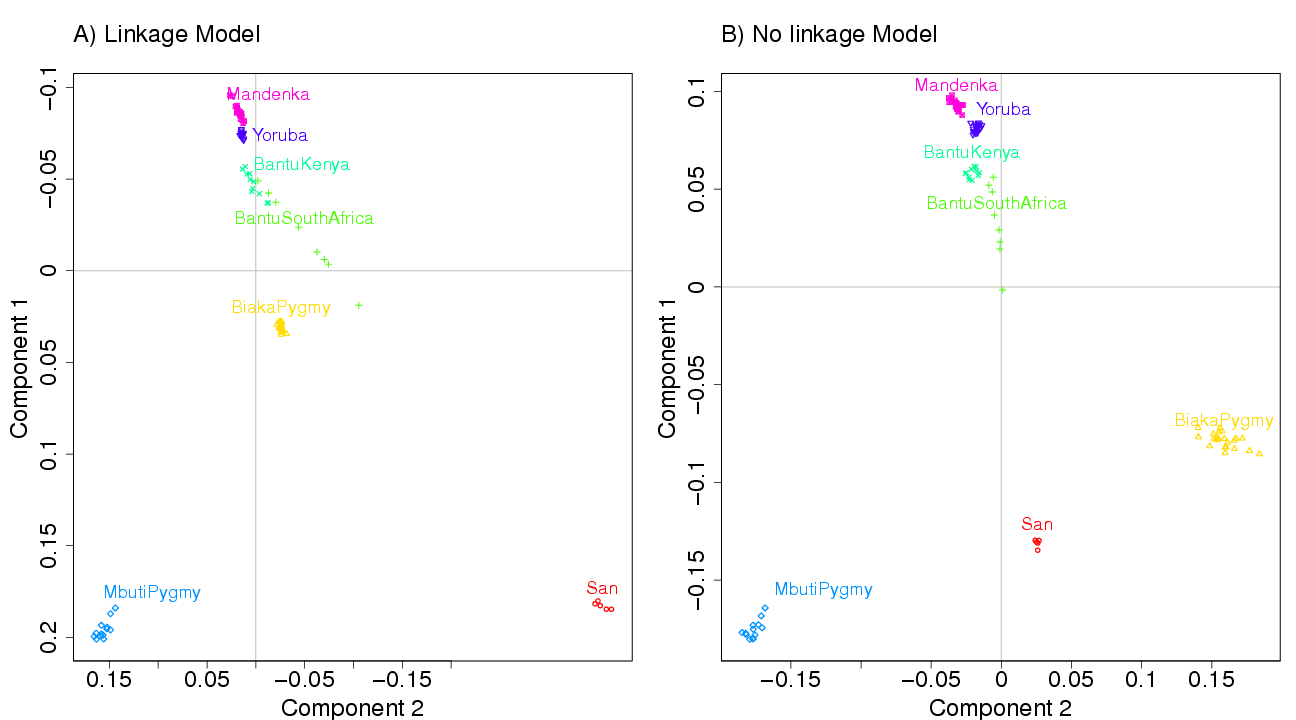

Supplement: Figure S34 — PCA for the continent of Africa. The first two components are shown; furhter structure will be present in the higher components. (TIFF) [file pgen.1002453.s034.tiff]

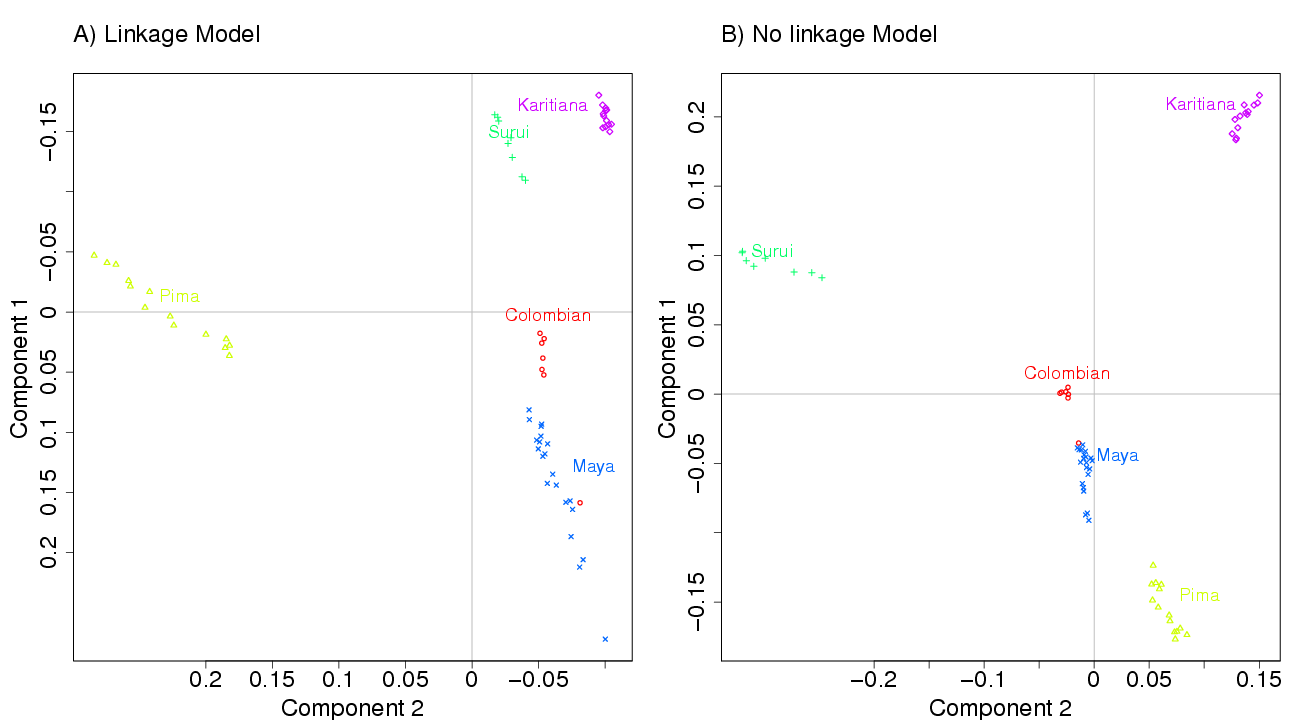

Supplement: Figure S35 — PCA for the continent of America. The first two components are shown; furhter structure will be present in the higher components. (TIFF) [file pgen.1002453.s035.tiff]

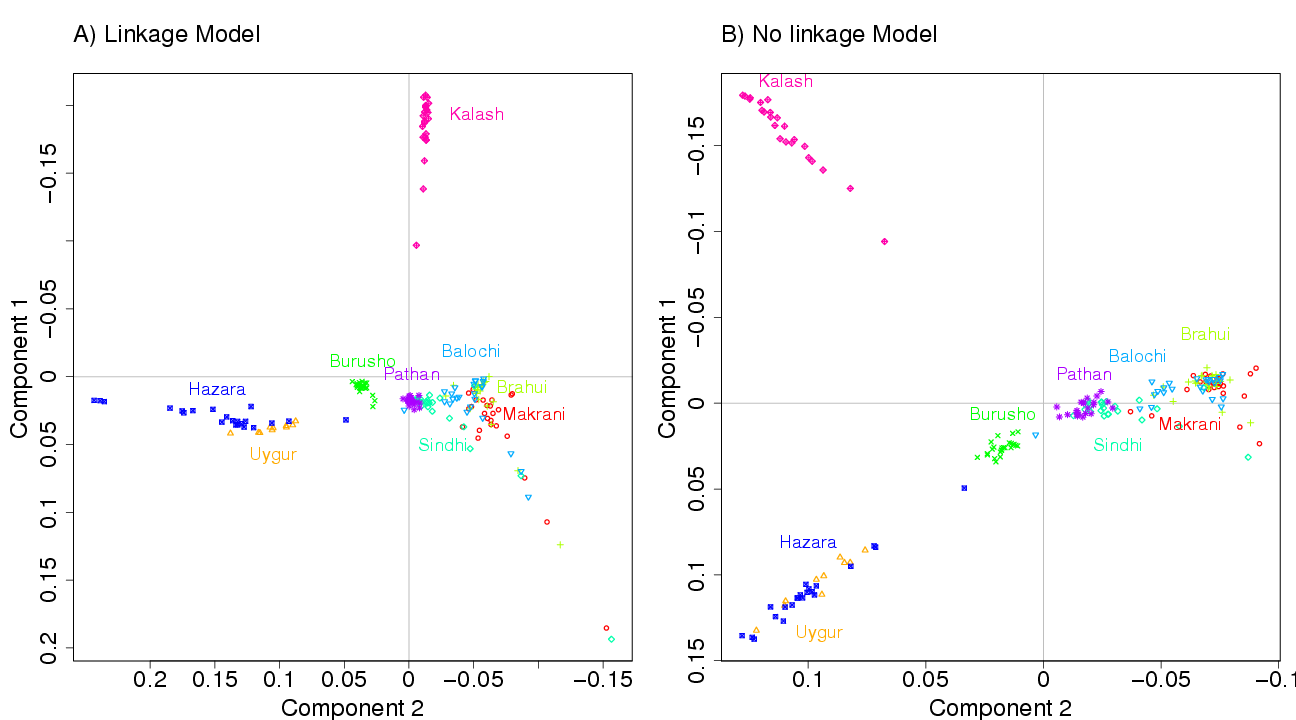

Supplement: Figure S36 — PCA for the continent of CentralSouthAsia. The first two components are shown; furhter structure will be present in the higher components. (TIFF) [file pgen.1002453.s036.tiff]

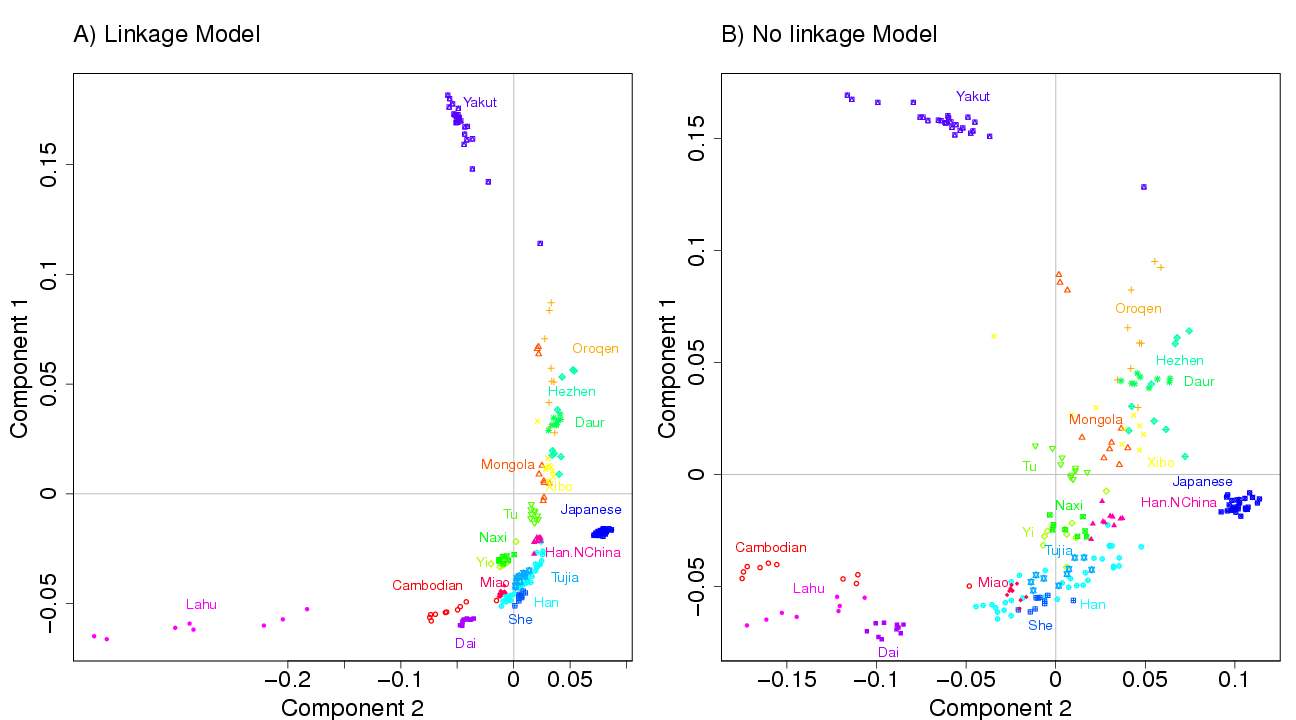

Supplement: Figure S37 — PCA for the continent of EastAsia. The first two components are shown; furhter structure will be present in the higher components. (TIFF) [file pgen.1002453.s037.tiff]

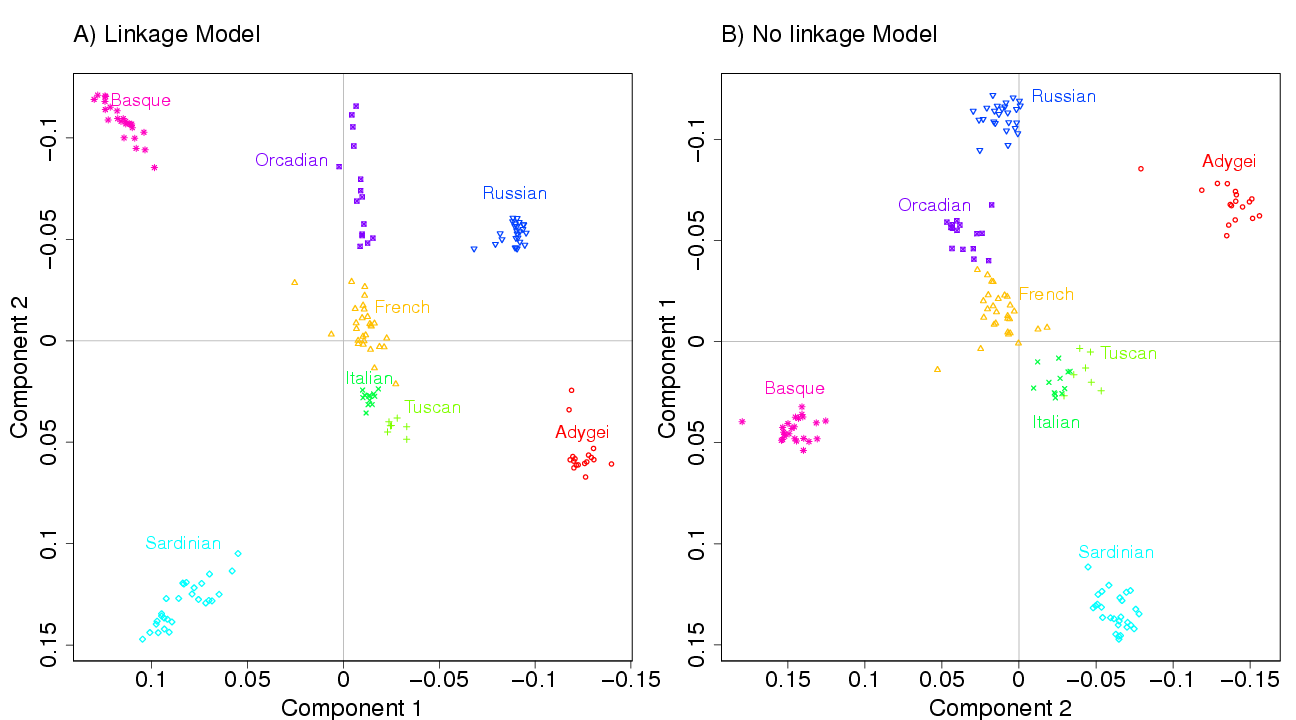

Supplement: Figure S38 — PCA for the continent of Europe. The first two components are shown; furhter structure will be present in the higher components. (TIFF) [file pgen.1002453.s038.tiff]

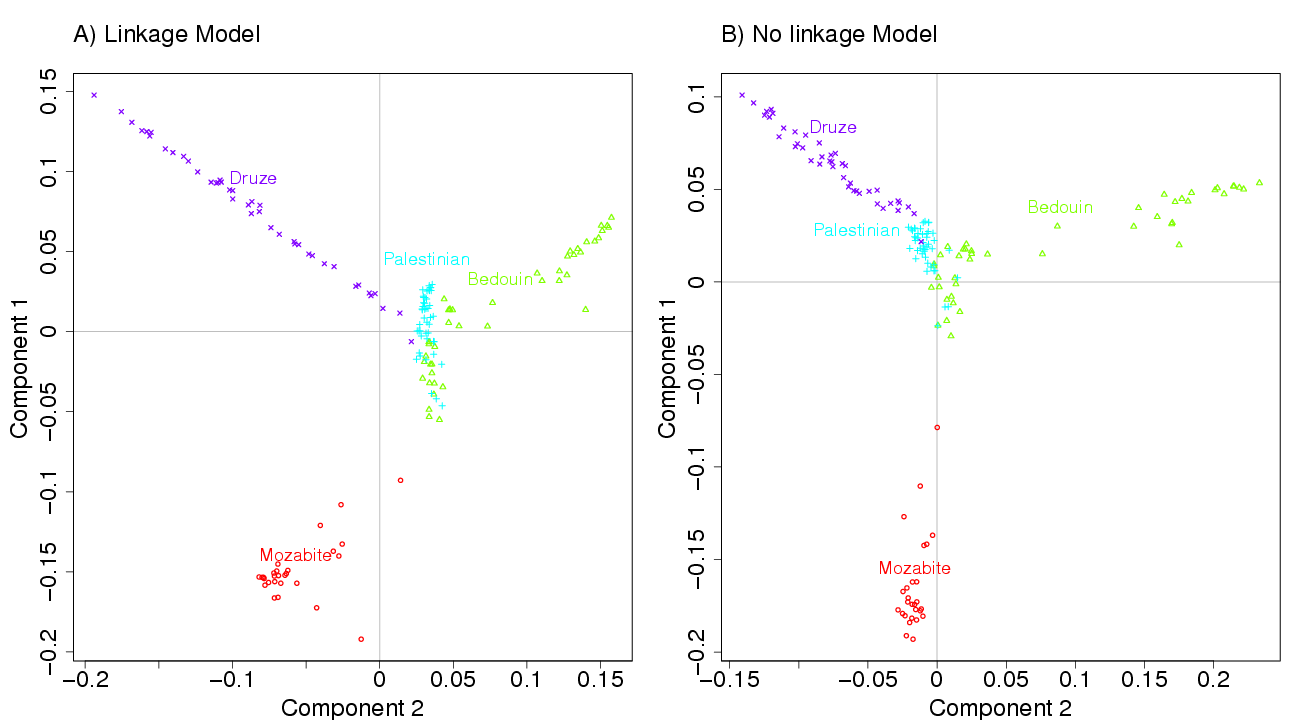

Supplement: Figure S39 — PCA for the continent of MiddleEast. The first two components are shown; furhter structure will be present in the higher components. (TIFF) [file pgen.1002453.s039.tiff]

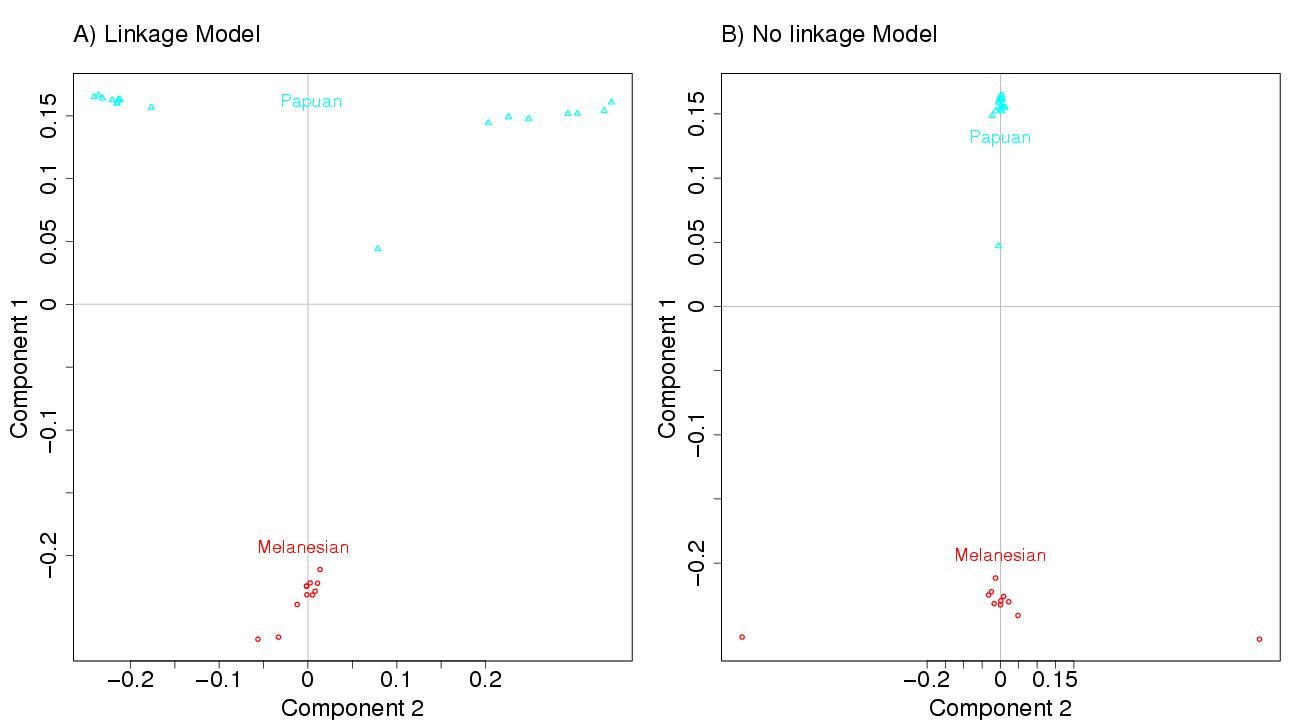

Supplement: Figure S40 — PCA for the continent of Oceania. The first two components are shown; furhter structure will be present in the higher components. (TIFF) [file pgen.1002453.s040.tiff]
