# Supplementary material for: Inference of Population Structure using Dense Haplotype Data
Source: PLoS Genet. 2012 Jan 26;8(1):e1002453. doi: 10.1371/journal.pgen.1002453 (PMC3266881; doi:10.1371/journal.pgen.1002453)
Supplement: Table S1 — Population labels assigned to “continents” for PCA. (PDF) [file pgen.1002453.s041.pdf]

Continent	Populations
Africa	San, BiakaPygmy, BantuSouthAfrica, BantuKenya, MbutiPygmy, Yoruba, Mandenka
America	Colombian, Pima, Surui, Maya, Karitiana
Central South Asia	Makrani, Uygur, Brahui, Burusho, Sindhi, Balochi, Hazara, Pathan, Kalash
East Asia	Cambodian, Mongola, Oroqen, Xibo, Yi, Tu, Naxi, Daur, Hezhen, Han, Tujia, She, Japanese, Yakut, Dai, Lahu, Han.NChina, Miao
Europe	Adygei, French, Tuscan, Italian, Sardinian, Russian, Orcadian, Basque
Middle East	Mozabite, Bedouin, Palestinian, Druze
Oceania	Melanesian, Papuan

Table 1: List of populations assigned to 'continents' for PCA.
