# Supplementary material for: Inference of Population Structure using Dense Haplotype Data
Source: PLoS Genet. 2012 Jan 26;8(1):e1002453. doi: 10.1371/journal.pgen.1002453 (PMC3266881; doi:10.1371/journal.pgen.1002453)
Supplement: Text S1 — Mathematical description of the Painting algorithm. (PDF) [file pgen.1002453.s042.pdf]

TEXT S1 Painting Algorithm

Li and Stephens (2003) described a likelihood based model that captures key features of the genealogical process with recombination while remaining computationally tractable for large datasets. Under the model, a chromosome is generated chunk-by-chunk by 'copying' from a conditional set of fixed haplotypes. In our notation, every individual consists of two haploids, each consisting of a single phased haplotype per chromosome. The L total SNPs in each haploid are listed one chromosome at a time, in order within each chromosome.

Suppose that we wish to generate a particular haploid $h_* = \{h_{*1}, ..., h_{*L}\}$, with h_{*l} the observed allele of h_* at site l, using j pre-existing donor haploids $h_1, ..., h_j$. Let $\vec{\rho} = \{\rho_1, ..., \rho_{L-1}\}$ be a vector of genetic distances, with ρ_l the population-scaled genetic distance between sites l and $l+1$ (i.e. $\rho_l = N_e g_l$, where N_e is analogous to the "effective population size" and g_l is the genetic distance in Morgans between sites l and $l+1$). (Between chromosomes, the genetic distance between the last site of the previous chromosome and the first site of the next chromosome is ∞.) Let $\vec{f} = \{f_1, ..., f_j\}$ be a vector of copying probabilities, with f_k the probability of copying from haploid h_k at any site. Let θ correspond to a per site mutation (or "imperfect copying") parameter. The conditional probability $\Pr(h_* \mid h_1, ..., h_j; \vec{\rho}, \vec{f}, \theta)$ is structured as a Hidden Markov model. Let $\vec{Y} = \{Y_1, ..., Y_L\}$ represent the hidden state sequence vector, with Y_l the existing haploid from the set h_1,...,h_j that haploid h_* copies from at site l. Switches in the haploid being copied between Y_l and Y_{l+1} occur as a Poisson process with rate ρ_l. The transition probabilities for Y between sites l and $l+1$ are as follows (we exclude $h_1, ..., h_j$ and the parameters from the left side of equations (1) and (2) below for ease of reading):

$$\Pr(Y_{l+1} = y_{l+1} | Y_l = y_l) = \begin{cases} \exp(-\rho_l) + \Big(1 - \exp(-\rho_l)\Big) f_{y_{l+1}} & \text{if } y_{l+1} = y_l; \\ \Big(1 - \exp(-\rho_l)\Big) f_{y_{l+1}} & \text{otherwise,} \end{cases} \quad (1)$$

The observed state sequence component of the Hidden Markov Chain, the probability of observing a particular allele given the haploid that h_* is copying from at a given SNP, allows for "imperfect" copying:

$$\Pr(h_{*l} = a | Y_l = y) = \begin{cases} 1.0 - \theta & h_{yl} = a; \\ \theta & h_{yl} \neq a. \end{cases} \quad (2)$$

Here h_{kl} refers to the allelic type of haploid k at SNP l. To calculate $\Pr(D) \equiv \Pr(h_* \mid h_1, ..., h_j; \vec{\rho}, \vec{f}, \theta)$, a summation is performed over all permutations of the copying process, i.e. a summation over all possible y, which can be accomplished efficiently using the forward algorithm (e.g. Rabiner 1989).

For all analyses presented here, we fix the mutation parameter θ to Watterson's estimate (Watterson 1975), as used by Li and Stephens (2003), i.e.

$$\theta = \frac{1}{2}\frac{\left(\sum_{i=1}^{j} 1/i\right)^{-1}}{j + \left(\sum_{i=1}^{j} 1/i\right)^{-1}}$$

for j total haploids. We fix each g_l by taking the build 36 genetic distance estimates from the HapMap website (`http://www.hapmap.org`), which were calculated using Phase II genotypes and averaging values across the three HapMap populations as described by the International HapMap Consortium (2007). We also fix each f_k to be $1/j$ for $k = 1, ..., j$, allowing for equal *a priori* probability of copying from each conditional haploid.

Calculating expected number of chunks copied:

The average number of chunks copied to a haploid $*$ is a random variable denoted $\hat{x}_i = \mathbb{E}_{l=1\cdots L}(X_{il})$, where X_{il} is the probability that a given locus l is a new haplotypic segment copied from individual i. To calculate $\hat{x}_1, ..., \hat{x}_j$, the posterior expected number of chunks for which haploid h_* copies from each of $h_1, ..., h_j$, respectively, we calculate $\hat{f}_{k,l}$, the probability haploid h_* is copying from haploid h_k at site l given at least one "switch" has occurred between $l-1$ and l. Again excluding parameters for ease of reading, let $\alpha_{kl} = \Pr(h_{*1}, ..., h_{*l}, Y_l = h_k)$ and $\beta_{kl} = \Pr(h_{*(l+1)}, ..., h_{*L} \mid Y_l = h_k)$. Then

$$\begin{aligned}\hat{x}_k &= \tfrac{\alpha_{k1}\beta_{k1}}{\Pr(D)} + \textstyle\sum_{l=1}^{L-1}(\tfrac{1}{\Pr(D)})\Big[\alpha_{k(l+1)}\beta_{k(l+1)} - \alpha_{kl}\beta_{k(l+1)}\Pr(h_{*(l+1)}|Y_{l+1} = h_k)\exp(-\rho_l)\Big] \\ &= \tfrac{\alpha_{k1}\beta_{k1}}{\Pr(D)} + \textstyle\sum_{l=1}^{L-1}\hat{f}_{k,l}.\end{aligned} \tag{3}$$

Note that we later drop the 'hat' notation for convenience, and form the matrix of all haplotype recipients $*$ as x_{ij}. Each row of x_{ij} corresponds to the vector $\hat{x}$ calculated above.

We calculate α_{kl} for $k = 1, ..., j$ in the following manner (Rabiner 1989):

1. $\alpha_{k1} = \Pr(h_{*1} \mid Y_1 = h_k) f_k$
2. $\alpha_{kl} = \Pr(h_{*l} \mid Y_l = h_k)\Big(\big[\sum_{i=1}^{j}\alpha_{i(l-1)}\big] f_k \big(1 - \exp(-\rho_l)\big) + \exp(-\rho_l)\alpha_{k(l-1)}\Big)$ for $l = 2, ..., L$.

We calculate β_{kl} for $k = 1, ..., j$ in the following manner (Rabiner 1989):

1. $\beta_{kL} = 1.0$
2. $\beta_{kl} = \big[\sum_{i=1}^{j}\beta_{i(l+1)} f_i \Pr(h_{*(l+1)} \mid Y_{l+1} = h_i)\big]\big(1 - \exp(-\rho_l)\big) + \exp(-\rho_l)\Pr(h_{*(l+1)} \mid Y_{l+1} = h_k)\beta_{k(l+1)}$ for $l = 1, ..., (L-1)$.

Calculating expected lengths of copied chunks:

To calculate $\hat{l}_1, ..., \hat{l}_j$, the posterior expected length (in Morgans) of the total genome for which haploid h_* copies from each of $h_1, ..., h_j$, respectively, we calculate the following (let $\mathrm{Pr}_h \equiv \mathrm{Pr}(h_{*(l+1)} \mid Y_{l+1} = h_k)$):

$$\begin{aligned} \hat{l}_k &= \tfrac{1}{\mathrm{Pr}(D)} \textstyle\sum_{l=1}^{L-1} g_l \Big[\alpha_{kl}\beta_{k(l+1)} \Big(\exp(-\rho_l) + (1.0 - \exp(-\rho_l)) f_k \Big) \mathrm{Pr}_h \\ &\quad + (1/2) \Big[\alpha_{kl}\beta_{kl} + \alpha_{k(l+1)}\beta_{k(l+1)} - 2\alpha_{kl}\beta_{k(l+1)} \Big(\exp(-\rho_l) + (1.0 - \exp(-\rho_l)) f_k \Big) \mathrm{Pr}_h \Big] \Big]. \end{aligned} \quad (4)$$

Note that this involves the approximation that at most only one change point occurs between neighbouring sampled sites. To get the expected length of *each* chunk copied from donor h_k, we divide equation (4) by equation (3) (i.e. $\hat{l}_k / \hat{x}_k$).

Calculating expected number of mutations:

To calculate $\hat{m}_1, ..., \hat{m}_j$, the posterior expected number of SNPs for which haploid h_* copies with mutation (i.e. emission) from each of $h_1, ..., h_j$, respectively, we calculate the following (let $\mathrm{I}_{[h_{*l} \neq h_{kl}]}$ be an indicator that the allelic type carried by h_* does not match the allelic type carried by h_k at SNP l):

$$\hat{m}_k = \tfrac{1}{\mathrm{Pr}(D)} \textstyle\sum_{l=1}^{L-1} \alpha_{kl}\beta_{kl} \mathrm{I}_{[h_{*l} \neq h_{kl}]}. \quad (5)$$

Using the E-M algorithm to estimate the scaling parameter N_e:

One can take a fixed N_e for calculating $\vec{\rho}$, or use the Expectation-Maximisation (E-M) algorithm to find a local maximum of N_e in the following manner. Start with an initial value of N_e (we take $N_e = 400,000/j$), and at each iteration of the E-M replace N_e with:

$$N_e^* = \frac{\sum_{l=1}^{L-1} \left([\sum_{k=1}^{j} \hat{f}_{k,l}][\rho_l]/[1.0 - \exp(-\rho_l)] \right)}{\sum_{l=1}^{L-1} g_l}, \quad (6)$$

where ρ_l and each $\hat{f}_{k,l}$ are calculated using the previous value of N_e. In analyses presented here, we used 10 iterations of E-M to get our final estimate of N_e.

Using the E-M algorithm to estimate the mutation parameter θ

One can take a fixed θ for calculating (2), or use the E-M to find a local maximum of θ in the following manner. Start with an initial value of θ (we start with Watterson's estimate of θ), and at each iteration of the E-M replace θ with:

$$\theta^* = \frac{\sum_{l=1}^{L} \left(\sum_{i=1}^{j} \alpha_{il}\beta_{il} I_{[h_{*l} \neq h_{il}]} / \Pr(D) \right)}{L}. \tag{7}$$

Here $I_{[h_{*l} \neq h_{il}]}$ is an indicator that the allele h_{*l} carried by the recipient is not equal to allele h_{il} carried by donor haploid i at SNP l, and each α_{il}, β_{il} and $\Pr(D)$ are calculated using the previous value of θ.

References

International HapMap Consortium, 2007 A second generation human haplotype map of over 3.1 million SNPs. Nature **449:** 851–61.

Li, N. and M. Stephens, 2003 Modeling Linkage Disequilibrium and Identifying Recombination Hotspots Using Single-Nucleotide Polymorphism Data. Genetics **165:** 2213–2233.

Rabiner, L., 1989 A tutorial on hidden markov models and selected applications in speech recognition. Proceedings of the IEEE **77:** 257–286.

Watterson, G., 1975 On the number of segregating sites in genetical models without recombination. Theor. Popul. Biol. **7:** 256–276.
