# Supplementary material for: Inference of Population Structure using Dense Haplotype Data
Source: PLoS Genet. 2012 Jan 26;8(1):e1002453. doi: 10.1371/journal.pgen.1002453 (PMC3266881; doi:10.1371/journal.pgen.1002453)
Supplement: Text S2 — Derivation of the fineSTRUCTURE Partition Posterior probability. (PDF) [file pgen.1002453.s043.pdf]

TEXT S2 Partition posterior probability evaluation

Here we define the full model for the coancestry matrix of expected copying counts x (dropping the 'hat' notation). Each row of x is distributed according to Multinomial likelihood $F(\cdot)$ as defined in Equation 1 of the main text:

$$x|\eta, P = \prod_{i=1}^{N} x_i|P_{q_i} \sim \prod_{i=1}^{N} F(\cdot|P_{q_i}), \tag{1}$$

where N is the number of individuals, P_{q_i} is the row of P corresponding to the population q_i containing individual i, K is the number of populations and η is the assignment of individuals to populations. Population membership q_i can be thought of as induced by η, as is the set of individuals found in a population S_a. A Dirichlet Process Prior (e.g. Teh 2010) is placed on η, which (approximately, for the purposes of exposition) means that for large $K^* \to \infty$ (and not generally equal to K), the probability of the number of individuals assigned to each population $\mathbf{n}$ (which is related, but not equal to $\{S_a\}_{a=1\cdots K}$) follows $\mathbf{n} \sim \text{Multinomial}(G)$ with $G \sim \text{Dirichlet}(\alpha/K^*, \cdots, \alpha/K^*)$. Note that in this view, many of these populations will be empty, leaving a finite number K of occupied populations.

There are many representations of a Dirichlet Process, with a common choice being $\{P_1, \cdots, P_N\} \sim \text{DP}(\alpha, G_0)$, where G_0 is the the 'base distribution', i.e. we sample parameters P_a from G_0, but obtain clustering by assigning the same parameters to multiple individuals. However, we choose an alternative description that suppressed G_0 which is simpler in our case.

The representation we find most natural is the joint assignment distribution induced on $\{\eta, K\}$, where K is the number of populations observed in our sample. This takes the form (Huelsenbeck and Andolfatto 2007):

$$p(\eta, K|\alpha, N) = \alpha^K \frac{\prod_{a=1}^{K} \Gamma(|S_a|)}{\prod_{i=1}^{N} (\alpha + i - 1)}, \tag{2}$$

where there are N individuals, and α is the 'concentration parameter' determining the number of occupied populations expected under the Dirichlet Process. In this case we can write the distribution of each probability vector P_a:

$$\{P_a, \cdots, P_K\}|\eta = P|\eta \sim \prod_{a=1}^{K} \text{Dirichlet}(\beta_a), \tag{3}$$

which is conjugate to F (and note that β_a is a vector of length K). This representation avoids the need to explicitly manage a G_0 that is itself a function of the number of populations K as is the case in our model. Note that we are free to use any distribution here in principle; this choice of Dirichlet distribution is not related to our use of a Dirichlet Process Prior.

From Equation 2, for fixed N and α the prior on η can be written:

$$\eta \sim p(\eta) \propto \alpha^K \prod_{b=1}^{K} \Gamma(|S_b|), \tag{4}$$

so that when $\alpha = 1$ all possible assignments are given equal prior weight. This allows us to control K in principle (though in practice the likelihood term overwhelms the prior on K), and applies the usual Bayesian penalty for having additional parameters (via additional populations), leading to low K solutions being favoured in the posterior. We wish to calculate the probability of a particular partition η:

$$P(\eta|x) \propto P(\eta) \prod_{a=1}^{K} L(x_{S_a}|\eta) \tag{5}$$

where $L(x_{S_a})$ is the likelihood of all the individuals in population a:

$$L(x_{S_a}) = \prod_{m \in S_a} P_{<m,S_a}(x_m) = \int \prod_{m \in S_a} F(x_m|P_m) dH_{<m,S_a}(P_m), \tag{6}$$

where $P_{<m,S_a}(x_m)$ is the probability of the data row x_m given the data for subset $(1, \cdots, m-1)$ of individuals in S_a, with an incremental probability distribution over P_a called (abusively) P_m. This is split up as the integral over the likelihood $F(x_m|P_m)$ of the probability of the parameters given the previous individuals data, $dH_{<m,S_a}(P_m)$. Conjugacy allows the incremental probability to be written as:

$$dH_{<m,S_a}(P_m) = \text{Dirichlet}\left(P_m; \left\{\beta_{ab} + d^{S_a}_{<m,b}\right\}_{b=1,\cdots,K}\right), \tag{7}$$

where β_{ab} is the prior given by Equation 2 of the main text and $d^{S_a}_{<m,b}$ are the counts from population S_b to population S_a for the individuals $[[1, \cdots, m-1]]$. The final posterior follows from Eq. 3.13 of Lange (2002):

$$P(\eta|x) \propto \alpha^K \prod_{a=1}^{K} \Gamma(|S_a|) \frac{\Gamma(\beta_a)}{\Gamma(d_a + \beta_a)} \prod_{b=1}^{K} \frac{\Gamma(\beta_{ab} + x_{ab})}{\Gamma(\beta_{ab}) \hat{n}^{x_{ab}}}. \tag{8}$$

References

Huelsenbeck, J. P. and P. Andolfatto, 2007 Inference of Population Structure Under a Dirichlet Process Model. Genetics **175:** 1787–1802.

Lange, K., 2002 *Mathematical and statistical methods for genetic analysis.* Springer.

Teh, Y. W., 2010 Dirichlet Process. In C. Sammut and G. Webb (Eds.), *Encyclopedia of Machine Learning*, pp. 280–287. Springer.
