# Supplementary material for: Inference of Population Structure using Dense Haplotype Data
Source: PLoS Genet. 2012 Jan 26;8(1):e1002453. doi: 10.1371/journal.pgen.1002453 (PMC3266881; doi:10.1371/journal.pgen.1002453)
Supplement: Text S3 — Mathematical details of the fineSTRUCTURE MCMC moves and acceptance probabilities. (PDF) [file pgen.1002453.s044.pdf]

TEXT S3 MCMC implementation

There are 4 moves, 2 of which are based on the SAMS scheme from Dahl (2003) and Pella and Masuda (2006), each of which is chosen with equal probability on each iteration.

SAMS proposal: from an initial state η two different individuals are chosen at random. If they are in the same population, it is split to form η'; if they are in different populations they are merged to form η'. On a split of population c (consisting of individuals S_c), the two individuals are each placed in new populations a and b and then each other individual in c is moved to i (either a or b) with probability:

$$P(m, S_i) = \frac{|S_i| \int F(x_m|p_m) dH_{<m,S_i}(p_m)}{|S_a| \int F(x_m|p_m) dH_{<m,S_a}(p_m) + |S_b| \int F(x_m|p_m) dH_{<m,S_b}(p_m)}, \quad (1)$$

where $\int F(x_m|p_m) dH_{<m,S}(p_m)$ is the incremental probability of adding an individual to the population. Because in our model all individuals must be assigned to populations, we approximate it using the source population only and use a rejection step to account for the discrepancy:

$$\int F(x_m|p_m) dH_{<m,S_a}(p_m) \approx \frac{P(a, \{i = 1, \cdots, m\}) P(c, \{i = 1, \cdots, m\})}{P(a, \{i = 1, \cdots, m-1\} P(c, \{i = 1, \cdots, m-1\}}, \quad (2)$$

The notation $i = 1, \cdots, m$ refers to the fact that these individuals have been moved to population a from population c previously when generating the proposal. The incremental probability is calculated as follows:

$$P(a) = \frac{\Gamma(\beta)}{\Gamma(d_a + \beta)} \prod_{b=1}^{K} \frac{\Gamma(\beta_b + x_{ab})}{\Gamma(\beta_b) \hat{n}^{x_{ab}}}, \quad (3)$$

and $P(b)$ is defined similarly. The probability of a given pair of split populations a and b from a single population c is therefore:

$$P(a, b|m \in S_c) = \prod_{m=1}^{|S_c|} \frac{|S_{q_m}| P(q_m)}{|S_a| P(a) + |S_b| P(b)}. \quad (4)$$

Once all individuals from population c have been placed, the new state η' is accepted with probability:

$$\min(1, P(\eta')/P(\eta) P(a, b|m \in S_c)). \quad (5)$$

A merge of two populations a and b in η similarly forms a new state η' and is accepted with probability

$$\min(1, P(a, b|m \in S_c) P(\eta')/P(\eta)). \quad (6)$$

'merge-and-split' (MAS) proposal: following the same strategy as above, but first forces a merge and then a split. If we call the initial two populations d and e in state η, they are merged to form an intermediate state c and resplit (according to the procedure above) to form populations a and b in state η'. If $\eta = \eta'$ the state is accepted (though we count a rejection for acceptance rate purposes) else the state η' is accepted with probability:

$$\min(1, P(a, b|m \in S_c)P(\eta')/P(\eta))P(c, d|m \in S_c). \tag{7}$$

individual proposal: move an individual to a new population. First choose an individual i at random. If $|S_{q_i}| > 1$ propose a new state η' with i moved to a population chosen uniformly from $(1, \cdots, K) \neq q_i$ and accept η' with probability:

$$\min(1, P(\eta')/P(\eta)). \tag{8}$$

parameter proposal: moves all hyperparameters independently.

- Delta: set $\delta' = \delta x$ with $x \sim U(-1, 1)$. Accept with probability $P(\eta|\delta')\Gamma(\delta'; k_\delta, \theta_\delta)/P(\eta|\delta)\Gamma(\delta; k_\delta, \theta_\delta)$, where the prior for δ is a gamma distribution with specified parameters; $(k_\delta, \theta_\delta) = (2, 0.01)$ throughout, providing a wide tailed distribution.

- F: set $f' = fx$ with $x \sim U(-1, 1)$. Accept with probability $P(\eta|f')\Gamma(f'; k_f, \theta_f)/P(\eta|f)\Gamma(f; k_f, \theta_f)$, where the prior for f is a gamma distribution with specified parameters; $(k_\delta, \theta_\delta) = (2, 0.01)$ throughout.

References

DAHL, D. B., 2003 An Improved Merge-Split Sampler For Conjugate Dirichlet Process Mixture Models. Technical Report 1086, University of Wisconsin – Madison. http://www.stat.tamu.edu/~dahl/papers/sams/tr1086.pdf.

PELLA, J. and M. MASUDA, 2006 The Gibbs and split–merge sampler for population mixture analysis from genetic data with incomplete baselines. Can. J. Fish. Aquat. Sci. **63:** 576–596.
