# Supplementary material for: Inference of Population Structure using Dense Haplotype Data
Source: PLoS Genet. 2012 Jan 26;8(1):e1002453. doi: 10.1371/journal.pgen.1002453 (PMC3266881; doi:10.1371/journal.pgen.1002453)
Supplement: Text S4 — Theory linking PCA, STRUCTURE and fineSTRUCTURE. This includes Propositions 1–4 and a brief summary of what they imply. (DOCX) [file pgen.1002453.s045.docx]

# **TEXT S4**

# **Theory linking PCA, STRUCTURE and fineSTRUCTURE**

## Introduction

In this section, we give (in the form of propositions, later backed up empirically) a detailed technical description of results described in the main paper regarding the links between different approaches to infer and analyse population structure. All results in this section refer to *unlinked* markers and we assume *haploid* data, though all results also extend to the diploid case. In a number of situations that we will highlight, we have used these results to naturally extend to the linked case.

Since the subsequent material is somewhat detailed and technical, we begin with a summary of the results, which all relate to *unlinked* markers:

1. We can approximately regard our coancestry matrix (see main text) as a rescaled version of a matrix commonly used to perform principal components analysis (PCA) on genetic data (Price et al. 2006).
2. For all forms of the normal-approximation likelihood (covering a wide class of models) the PCA matrix contains all of the information available on population structure. The form of this likelihood demonstrates a close link between model-based analyses such as that performed by STRUCTURE (Pritchard et al. 2000), and PCA analyses of the type referred to in Proposition 1. Specifically, we show that for large datasets, the PCA matrix of Proposition 1 forms a set of sufficient statistics for the “STRUCTURE likelihood” – i.e. the likelihood of the data used by STRUCTURE’s “no linkage” model, and by other similar software applications. Thus, in practice we expect that almost all the information accessible to even model-based approaches is contained within the PCA matrix, and hence also within our coancestry matrix. The permitted population structure is very general and therefore includes both discrete population models and continuous models capturing admixture.
3. We derive an asymptotic approximation of the likelihood from Proposition 2 that takes a particularly simple form under the assumption of a large number of individuals and small drift.
4. Provided population structure is not very strong, and again for large datasets, the multinomial likelihood form that we use in fineSTRUCTURE gives the same asymptotic likelihood as that used by STRUCTURE (Pritchard et al. 2000) found in Proposition 3. The proof also leads to an explicit rescaling of the multinomial likelihood (our “$c$” factor), and implies an algorithm to infer $c$ in general, whose efficacy we test via simulation in a range of scenarios.

The results of Propositions 1 and 4, in particular, naturally motivate our approaches for analysing linked data, which we describe within the relevant sections.

For clarity, this section has slightly modified notation. We begin by defining quantities used throughout this section.

### Notes on notation

We will make heavy use of vector notation. Matrices and vectors will be in bold, and scalar quantities will be in italics. For a matrix $\mathbf{M}$ of dimension $M\times N$, $M_{ij}$ selects an element, $\mathbf{M}_{i*}$ selects the $i$th row and $\mathbf{M}_{*j}$ selects the $j$th column. We define $\mathbf{1}$ as the (appropriate length) vector containing all ones, hence $\mathbf{M1}\boldsymbol{=}\mathbf{M}_{.*}$ is the vector of row sums, $\boldsymbol{1}^{T}\mathbf{M}\mathbf{=}\mathbf{M}_{*.}$ is the vector of column sums, and $\boldsymbol{1}^{T}\mathbf{M1}\mathbf{=}M_{..}$ is the sum over all elements of $\mathbf{M}$. Where ambiguity is possible we denote the length of one-vectors by subscripts e.g. $\mathbf{1}_{L}$. Note that unlike in the main text we do not use lower case to denote elements.

### Definitions and Initial Assumptions

As in the main text, suppose we have data for $N$ individuals, drawn from $K$ populations, and let $q_{i}$ be the population of individual $i$. Let $L$ be the total number of segregating sites (after appropriate filtering). Let $n_{a}$be the total number of individuals from each population $a$.

We define $\mathbf{D}$ to be the *raw data matrix*. Specifically, elements $D_{il}$ are binary, taking the value 1 if haplotype $i$ carries that form at SNP $l$ and 0 otherwise. We assume no missing data for the purposes of our derivations. We also assume all sites are biallelic, with sites with one or fewer occurrences of 0 or 1 removed and regarded as uninformative.

We define $\mathbf{X}$ to be the observed *coancestry matrix* for these data (see main text, where this is called $x$), where $X_{ij}$ is the total *expected* number of chunks – each consisting of exactly one SNP in the unlinked case – that individual $i$ copies from individual $j$. We view this expectation as calculated using the Li and Stephens (2003) model for genetic data applied with an infinite recombination rate, and arbitrarily small mutation rate in this setting, and thus this matrix is determined by the data $\mathbf{D}$. By the definition of the Li and Stephens algorithm, we have:

$$X_{ij}=\sum_{l=1}^{L} \left[ \frac{D_{il}D_{jl}}{\sum_{k\neq i} D_{kl}}+\frac{\left( 1-D_{il} \right)\left( 1-D_{jl} \right)}{\sum_{k\neq i} \left( {1-D}_{kl} \right)} \right].$$

(Note that if a non-zero mutation rate is instead used, the matrix obtained is simply a rescaled version of this, with a constant term added, except at SNPs with seen only once in the sample, which we are assuming have been removed from the data.)

We define the *parameter matrix* $\mathbf{P}$ to be the underlying donor matrix, where $L\mathbf{P}$ gives the expected values for each entry of $\mathbf{X}$ and so $P_{ij}$ gives the fraction of chunks that individual $i$ is expected to copy from individual $j$. In particular, note we alter notation slightly from the text and view $\mathbf{P}$ as an $N\times N$ matrix (which will have a block-like structure if individuals are assigned to discrete populations).

We define the *Eigenstrat PCA matrix,* $\mathbf{E}$ to be the matrix used for principal components analysis in the Eigenstrat approach (Price et al. 2006). To construct this matrix, viewing mutations as corresponding to columns in the data matrix, columns are first scaled to have zero mean and unit variance, and subsequently the rescaled data matrix is multiplied by its transpose, to give the following:

$$E_{ij}=\sum_{l=1}^{L} \frac{(D_{il}-\hat{f_{l}})(D_{jl}-\hat{f_{l}})}{\hat{f_{l}}(1-\hat{f_{l}})}.$$

Here, we define $\hat{f_{l}}$ to be the observed sample frequency of the “1” allele at SNP $l$:

$$\hat{f_{l}}=\frac{1}{N}\sum_{i=1}^{N} D_{il}$$

We will make use of several assumptions, which are summarized here and more fully defined when they are introduced.

1. **No linkage disequilibrium** between loci within populations**.** Loci are independent conditional on underlying population assignments such that $cov\left( \mathbf{D}_{*l},\mathbf{D}_{*l^{'}} \right)=0$ within populations for $l \neq l^{'}$.
2. **Large sample size.** We only require the leading order contribution in terms of $N^{-1}$ where $N$ is the number of individuals sampled*.*
3. **Normally distributed drift.** Individuals are sampled in such a way that the drift can be approximated by a normal distribution. In population models, this means that the sample contains many individuals per population, and that rare SNPs are excluded.
4. **Large number of loci.** We only require the leading order contribution in terms of $L^{-1}$ where $L$ is the number of loci.
5. **Weak drift.** We only require the leading order contribution of the drift, defined in Section S4.2.
6. (Technical assumption). The distribution of the (weighted) average frequency across all sampled individuals does not contain information on population structure.
7. **More loci than individuals.** The number of individuals $N$ is small in relation to the number of loci $L$.
8. (Technical assumption). We require to integrate out the ancestral SNP frequencies, for which we assume that any prior on these is weak compared to the likelihood.

## S4.1: The coancestry matrix and the PCA matrix

The main result of this section is the following proposition.

Proposition 1: the PCA and coancestry matrices are related

For haploid variation data where sites are treated as unlinked (A1), the off-diagonal elements of the Eigentrat PCA matrix $\mathbf{E}$ and the observed coancestry matrix $\mathbf{X}$ are approximately related by the following equation:

$$E_{ij}=\left( N-1 \right)X_{ij}-L+O\left( N^{-1} \right), i\neq j.$$

#### Proof:

Let $\hat{f_{l}'}=\frac{1}{N-1}\sum_{k\neq i} D_{kl}$ be the empirical frequency of all SNPs excluding individual $i$. From the definition:

$$X_{ij}=\sum_{l=1}^{L} \left[ \frac{D_{il}D_{jl}}{\sum_{k\neq i} D_{kl}}+\frac{\left( 1-D_{il} \right)\left( 1-D_{jl} \right)}{\sum_{k\neq i} \left( {1-D}_{kl} \right)} \right]$$

$$=\frac{1}{N-1}\sum_{l=1}^{L} \left[ \frac{D_{il}D_{jl}}{\hat{f_{l}'}}+\frac{\left( 1-D_{il} \right)\left( 1-D_{jl} \right)}{1-\hat{f_{l}'}} \right]$$

$$=\frac{1}{N-1}\sum_{l=1}^{L} \left[ \frac{\left( D_{il}-\hat{f_{l}'} \right)\left( D_{jl}-\hat{f_{l}'} \right)}{\hat{f_{l}'}\left( 1-\hat{f_{l}'} \right)}+1 \right]$$

$$=\frac{L}{N-1}+\frac{1}{N-1}\sum_{l=1}^{L} \left[ \frac{\left( D_{il}-\hat{f_{l}} \right)\left( D_{jl}-\hat{f_{l}} \right)+\frac{1}{N-1}\left( D_{il}-\hat{f_{l}} \right)^{2}}{\hat{f_{l}}\left( 1-\hat{f_{l}} \right)-\frac{1}{N-1}\left( D_{il}-\hat{f_{l}} \right)^{2}} \right]$$

$$=\frac{L}{N-1}+\frac{1}{N-1}\sum_{l=1}^{L} \left[ \frac{\left( D_{il}-\hat{f_{l}} \right)\left( D_{jl}-\hat{f_{l}} \right)}{\hat{f_{l}}\left( 1-\hat{f_{l}} \right)} \right]+\text{O}\left( N^{-2} \right)$$

$$=\frac{L}{N-1}+\frac{E_{ij}}{N-1}\text{+O}\left( N^{-2} \right)$$

The proposition follows on rearrangement for $E_{ij}$.$∎$

Note that we can write $\boldsymbol{X\approx(E}-diag\left( \mathbf{E} \right))/(N-1)$.

Using properties of the PCA matrix, we can then show that this means the Eigenstrat PCA matrix $\mathbf{E}$ and the observed coancestry matrix $\mathbf{X}$ have similar eigenvectors.

Corollary 1: the PCA and coancestry matrices have the same eigenvectors

For haploid variation data where sites are treated as unlinked, assuming large population size (A2) the PCA matrix $\mathbf{E}$ and the observed coancestry matrix $\mathbf{X}$ have approximately identical eigenvectors.

#### Proof:

First note that

$$E\left( E_{ii} \right)=E\left[ \sum_{l=1}^{L} \frac{\left( D_{il}-\hat{f_{l}} \right)^{2}}{\hat{f_{l}}\left( 1-\hat{f_{l}} \right)} \right]=E\left[ \sum_{l=1}^{L} \frac{{D_{il}}^{2}-2D_{il}\hat{f_{l}}+{\hat{f_{l}}}^{2}}{\hat{f_{l}}\left( 1-\hat{f_{l}} \right)} \right]=\sum_{l=1}^{L} \frac{\hat{f_{l}}-2{\hat{f_{l}}}^{2}+{\hat{f_{l}}}^{2}}{\hat{f_{l}}\left( 1-\hat{f_{l}} \right)}=L,$$

since $E\left( {D_{il}}^{2} \right)=E\left( D_{il} \right)=g_{l}$, the ancestral frequency of the SNP. Additionally, assuming that drift is small, we can replace the denominator $\hat{f_{l}}\left( 1-\hat{f_{l}} \right)$ by $g_{l}\left( 1-g_{l} \right)$ and the result follows. (Note that in many sensible models $g_{l}=\hat{f_{l}}$ as $N$ becomes large, so the result may hold even when drift is moderate).

Now note that the row and column sums of $\mathbf{E}$ are identically zero, so trivially the $N$-vector $\text{1}$ is an eigenvector with eigenvalue 0. Similarly the same eigenvector has eigenvalue 1 for the co-ancestry matrix $\mathbf{X}$. Let **v** be any *other* eigenvector of $\mathbf{E}$ with eigenvalue $\lambda$. We must then have $\sum_{i=1}^{N} v_{i}=0$ and so

$$\mathbf{Xv}=\frac{1}{N-1}\left[ \mathbf{Ev-}diag\left( E_{11},E_{22},\cdots,E_{NN} \right)\mathbf{v} \right]+\frac{L}{N-1}\sum_{i=1}^{N} v_{i}+O\left( N^{-1} \right)$$

$$\approx\frac{1}{N-1}\left[ \lambda-L \right]\mathbf{v}+O\left( N^{-1} \right).$$

Therefore **v** is also an eigenvector of $\mathbf{X}$ with transformed eigenvalue $\frac{1}{N-1}\left[ \lambda-L \right]$ (when *N* is large, hence the approximation) as required.$∎$

#### Discussion of Proposition 1:

The above proposition suggests that a principal components approach based on the coancestry matrix should yield results comparable to those from using standard approaches. We confirmed this fact in the main text. Note that although the proposition excludes the diagonal of the PCA matrix, in practice since each row or column sum of this matrix is exactly zero, we do not expect there to be (much) information from these diagonals. In our coancestry matrix, each row and column automatically sums to 1, with diagonal entries set to zero, and so the information “lost” using this approach, relative to the standard PCA approach, is the value of the row and column sums in the PCA matrix. However, under weak assumptions we have shown that asymptotically the expected value of each element along the diagonal of the PCA matrix is $L$, so the off-diagonal sums in the PCA matrix have expected value $-L$, and add little or no information about population structure.

In implementing these ideas in practice, we modified our matrix slightly to ensure that eigenvalues were ranked equivalently between our approach and the PCA matrix itself (with the most historically relevant eigenvalues taking the largest values). By corollary 1, the eigenvalues of $\mathbf{X}$ are shifted and scaled relative to those from the PCA matrix, with one large eigenvalue equal to 1. To fix this, we first removed the shift by setting diagonal elements equal to the column sums. In the unlinked case, this is identically equal to $\frac{1}{N-1}L$ for each column, and adding these diagonal values trivially leaves eigenvectors unchanged and increases eigenvalues by this constant, removing the shift. To rescale the large eigenvalue to the value zero, we next subtract column means from each entry of the matrix. Again, in the unlinked case this simply removes $\frac{1}{N-1}L$ from each entry of the matrix, so has no effect on either the eigenvalues or eigenvectors, apart from the large eigenvalue corresponding to the *N*-vector $\text{1}$. Finally, the resultant matrix $\mathbf{X}^{'}$will have eigenvalues that are simply rescaled by a factor $\frac{1}{N-1}$ relative to the PCA matrix.

Our new PCA approach obviously extends trivially to using the equivalent coancestry matrix in the *linked* case. For this case, we made one small final modification, to account for the fact that in the linked case our coancestry matrix need not be exactly symmetric, with the resultant drawback that left and right eigenvectors will differ slightly. To fix this, we note that if $\mathbf{G}$ is a general symmetric matrix, then the eigenvectors of $\mathbf{G}\mathbf{G}^{T}$are identical. Motivated by this fact, and following other PCA approaches, we performed PCA in the general case on the matrix $\mathbf{X}'{\mathbf{X}'}^{T}$. In the linked case, this symmetrisation appeared to improve results slightly – other approaches we tried did not yield obvious improvements.

## S4.2: The coancestry matrix and model-based approaches to inferring structure

### Modelling population structure using a normally distributed drift matrix

In general, population structure between separated groups is often modelled using the concept of genetic drift between populations (for details, see e.g. Pritchard et al. (2000), Nicholson et al. (2002), Patterson et al. (2006)). We begin by defining $g_{la}$ to be the frequency of the mutation at the $l$th locus in population $a$. Several models assume a joint prior distribution on $g_{la}$ with some shared mean $f_{l}$ and variance matrix $f_{l}\left( 1-f_{l} \right)\mathbf{G}$. Conceptually, $f_{l}$ can be thought of as representing the frequency of the mutation in an ancestral population before population-specific genetic drift results in new frequencies in each separate population group. The matrix $\mathbf{G}$ defines the covariance structure of this drift between populations. This allows, in general, for correlated drift among populations. Let us go further and assume as in the original Nicholson et al. (2002) formulation (which approximates genetic diffusion models) that

$$\mathbf{g}_{l}\mathbf{=}\left( g_{l1},g_{l2},\cdots,g_{lK} \right)\boldsymbol{\sim}N(f_{l}\mathbf{1}_{K},f_{l}\left( 1-f_{l} \right)\mathbf{G})$$

This “Normal drift” approximation is expected to be quite accurate, provided drift $\mathbf{G}$ is relatively small and (as below) provided it is applied to SNPs that are not at frequency very close to 0 or 1, a condition reasonably appropriate for many datasets involving ascertained SNPs, and which can be easily imposed more generally.

In all that follows, we use only the above assumption regarding drift, and thus our results apply across a fairly general range of settings. In particular, allowing for correlated drift means that if populations successively split, in a tree-like structure, the results still follow. Further, the no-linkage admixture model used by, for example, the program STRUCTURE (Pritchard et al. 2000) can be thought of as simply using an appropriate choice of $\mathbf{G}$ (with each individual representing a population).

Now at an individual level, define $f_{li}$ to be the frequency for SNP $l$ in the population $q_{i}$to which individual *i* belongs: $f_{li}=g_{lq_{i}}$. From the above, we have immediately that

$$\mathbf{f}_{l}\mathbf{|}f_{l}\mathbf{=}\left( f_{l1},f_{l2},\cdots,f_{lN} \right)\mathbf{|}f_{l}\boldsymbol{\sim}N(f_{l}\mathbf{1}_{N},f_{l}\left( 1-f_{l} \right)\mathbf{H})$$

where $\mathbf{H}$ is the individual-level matrix giving drift (from $\mathbf{G}$) between pairs of individuals. (This matrix is singular in general, but that will not affect our analysis.) We will formulate our likelihoods in terms of $\mathbf{f}_{l}$. (A notation reminder: $\mathbf{f}_{l}$ is the vector of population SNP frequencies, and $f_{l}$ is the ancestral frequency).

### Constructing an approximate likelihood

For the remainder of this section, we study inference under normally distributed drift, using an approximation to the likelihood of the data. For this approximation to be valid, we assume (A3) that the number $n_{a}$ of individuals sampled from each population $a$, and corresponding population-specific allele frequencies $f_{la}$ are such that we may use a Normal approximation to the binomial sampling likelihood of the observed data. In practice, this means $n_{a}$should be large, at least ≥20, and $f_{la}$ should not be very close to 0 or 1.

Later, we will need an additional technical assumption (A6) that mean allele frequencies averaged over all populations are uninformative for population structure. Provided that drift is weak we expect that typically these can contain at most weak information about the underlying population assignments, justifying this assumption.

We spend the remainder of this section deriving a form for the approximate likelihood, under these two (main) assumptions.

Preliminary to Proposition 2: Single locus likelihood

Under assumptions A1-3, and assuming also that a weak prior is placed on the ancestral SNP frequency (A8), the likelihood for a single locus $l$ can be expressed in the following form:

$$L\left( \mathbf{D}_{*l} \right)\propto\left\| \boldsymbol{\Sigma} \right\|^{-\frac{1}{2}}\left[ \mathbf{1}^{\mathbf{T}}\boldsymbol{\Sigma}^{-\mathbf{1}} \mathbf{1} \right]^{-\frac{\mathbf{1}}{\mathbf{2}}}exp\left[ -\frac{1}{2\hat{f_{l}}\left( 1-\hat{f_{l}} \right)}\left( \mathbf{D}_{*\boldsymbol{l}}-\hat{f_{l}}\mathbf{1} \right)^{\boldsymbol{T}}\left( \boldsymbol{\Sigma}^{-\mathbf{1}}-\frac{\boldsymbol{\Sigma}^{-\mathbf{1}} \mathbf{1}\text{1}^{\boldsymbol{T}}\boldsymbol{\Sigma}^{-\mathbf{1}}}{\mathbf{1}^{\mathbf{T}}\boldsymbol{\Sigma}^{-\mathbf{1}} \mathbf{1}} \right)\left( \mathbf{D}_{*\boldsymbol{l}}-\hat{f_{l}}\mathbf{1} \right) \right].$$

Where $\boldsymbol{\Sigma}\mathbf{=}\mathbf{I}\mathbf{+}\mathbf{H}$ is the (non-singular) covariance matrix.

#### Proof and derivation:

Conditional on the underlying allele frequencies, the observed allele counts in a (haploid) individual *i* from population $a$ are independent among individuals, and simply Bernoulli with mean $f_{li}=g_{la}$ and variance $f_{li}(1-f_{li}$). By the assumptions, we may approximate the likelihood by behaving as if data are taken from a Normal distribution. If drift is weak, the overall sample mean frequency $\hat{f_{l}}$ can be used to approximate $f_{li}$ in the variance term and we obtain a joint distribution for the data vector $\mathbf{D}_{\boldsymbol{*l}}$for all $N$ individuals, at SNP $l$:

$$\mathbf{D}_{\mathbf{*}\boldsymbol{l}}| f_{l1},f_{l2}\cdots,f_{lK}\dot{\sim}N\left( \mathbf{f}_{l},\hat{f_{l}}\left( 1-\hat{f_{l}} \right)\text{I} \right).$$

Making the similar approximation

$$\mathbf{f}_{l}\mathbf{=}\left( f_{l1},f_{l2},\cdots,f_{lN} \right)\boldsymbol{\sim}N\left( f_{l}\mathbf{1}_{\mathbf{N}},f_{l}\left( 1-f_{l} \right)\mathbf{H} \right)\dot{\sim}N\left( f_{l}\mathbf{1}_{\mathbf{N}},\hat{f_{l}}\left( 1-\hat{f_{l}} \right)\mathbf{H} \right),$$

integrating out the population frequencies we have by properties of Normal distributions:

$$\mathbf{D}_{\mathbf{*}\boldsymbol{l}}\mathbf{|}f_{l}\dot{\sim}N\left( f_{l}\mathbf{1}_{\mathbf{N}},\hat{f_{l}}(1-\hat{f_{l}})\left[ \mathbf{I+H} \right] \right).$$

Using the notation $\boldsymbol{\Sigma= I+H}$, the likelihood is then:

$$L\left( \mathbf{D}_{\mathbf{*}\boldsymbol{l}}\mathbf{|}f_{l} \right)=\left[ 2\pi\hat{f_{l}}(1-\hat{f_{l}}) \right]^{-N/2}\left\| \boldsymbol{\Sigma} \right\|^{-1/2}exp\left[ -\frac{1}{2\hat{f_{l}}(1-\hat{f_{l}})}\left( \mathbf{D}_{\mathbf{*}\boldsymbol{l}}\mathbf{-}f_{l}\mathbf{1} \right)^{\boldsymbol{T}}\boldsymbol{\Sigma}^{\mathbf{-1}}\left( \mathbf{D}_{\mathbf{*}\boldsymbol{l}}\mathbf{-}f_{l}\mathbf{1} \right) \right].$$

Define

$$\text{a}=\frac{\boldsymbol{\Sigma}^{-\mathbf{1}} \mathbf{1}}{\mathbf{1}^{\mathbf{T}}\boldsymbol{\Sigma}^{-\mathbf{1}} \mathbf{1}}.$$

Let $Y=\text{a}^{\boldsymbol{T}}\mathbf{D}_{*l}\boldsymbol{.}$Clearly $Y$ is normally distributed:

$$Y\boldsymbol{|}f_{l}\sim N\left( \boldsymbol{a}^{\boldsymbol{T}}f_{l}\mathbf{1}_{\mathbf{N}},\hat{f_{l}}\left( 1-\hat{f_{l}} \right)\text{a}^{\boldsymbol{T}}\boldsymbol{\Sigma}\text{a} \right)\sim N\left( f_{l},\frac{\hat{f_{l}}\left( 1-\hat{f_{l}} \right)}{\mathbf{1}^{\mathbf{T}}\boldsymbol{\Sigma}^{\mathbf{-1}}\mathbf{1}} \right).$$

Thus, $Y$ has mean $f_{l}$ and variance which decreases as we increase sample size.

We can rewrite the exponent term in our likelihood:

$$\left( \mathbf{D}_{*\boldsymbol{l}}-f_{l}\mathbf{1} \right)^{\boldsymbol{T}}\boldsymbol{\Sigma}^{-\mathbf{1}}\left( \mathbf{D}_{*\boldsymbol{l}}-f_{l}\mathbf{1} \right)=\left( \mathbf{D}_{*\boldsymbol{l}}-\mathbf{1}Y+\mathbf{1}Y-f_{l}\mathbf{1} \right)^{\boldsymbol{T}}\boldsymbol{\Sigma}^{-\mathbf{1}}\left( \mathbf{D}_{*\boldsymbol{l}}-\mathbf{1}Y+\mathbf{1}Y-f_{l}\mathbf{1} \right)$$

$$=\left( \mathbf{D}_{*\boldsymbol{l}}-\mathbf{1}Y \right)^{\boldsymbol{T}}\boldsymbol{\Sigma}^{-\mathbf{1}}\left( \mathbf{D}_{*\boldsymbol{l}}-\mathbf{1}Y \right)+2\left( \mathbf{D}_{*\boldsymbol{l}}-\mathbf{1}Y \right)^{\boldsymbol{T}}\boldsymbol{\Sigma}^{-\mathbf{1}}\left( \mathbf{1}Y-\mathbf{1}f_{l} \right)+\left( \mathbf{1}Y-\mathbf{1}f_{l} \right)^{\boldsymbol{T}}\boldsymbol{\Sigma}^{-\mathbf{1}}\left( \mathbf{1}Y-\mathbf{1}f_{l} \right)$$

$$=\left( \mathbf{D}_{*\boldsymbol{l}}-\mathbf{1}Y \right)^{\boldsymbol{T}}\boldsymbol{\Sigma}^{-\mathbf{1}}\left( \mathbf{D}_{*\boldsymbol{l}}-\mathbf{1}Y \right)+2\left( \mathbf{D}_{*\boldsymbol{l}} \right)^{\boldsymbol{T}}\left( \text{I}-\mathbf{a}\text{1}^{\boldsymbol{T}} \right)\boldsymbol{\Sigma}^{-\mathbf{1}}\mathbf{1}\left( Y-f_{l} \right)+\left( Y-f_{l} \right){\mathbf{1}^{\mathbf{T}}\boldsymbol{\Sigma}}^{-\mathbf{1}}\mathbf{1}\left( Y-f_{l} \right)$$

$$=\left( \mathbf{D}_{*\boldsymbol{l}}-\mathbf{1}Y \right)^{\boldsymbol{T}}\boldsymbol{\Sigma}^{-\mathbf{1}}\left( \mathbf{D}_{*\boldsymbol{l}}-\mathbf{1}Y \right)+2\left( \mathbf{D}_{*\boldsymbol{l}} \right)^{\boldsymbol{T}}\left( \boldsymbol{\Sigma}^{-\mathbf{1}}\mathbf{1}-\boldsymbol{\Sigma}^{-\mathbf{1}} \mathbf{1} \right)\left( Y-f_{l} \right)+\mathbf{1}^{\mathbf{T}}\boldsymbol{\Sigma}^{-\mathbf{1}} \mathbf{1}\left( Y-f_{l} \right)^{\mathbf{2}}$$

$$=\left( \mathbf{D}_{*\boldsymbol{l}}-\mathbf{1}Y \right)^{\boldsymbol{T}}\boldsymbol{\Sigma}^{-\mathbf{1}}\left( \mathbf{D}_{*\boldsymbol{l}}-\mathbf{1}Y \right)+\mathbf{1}^{\mathbf{T}}\boldsymbol{\Sigma}^{-\mathbf{1}} \mathbf{1}\left( Y-f_{l} \right)^{\mathbf{2}}$$

and substituting back in we find:

$$L\left( \mathbf{D}_{*l}\mathbf{|}f_{l} \right)=\left[ 2\pi\hat{f_{l}}(1-\hat{f_{l}}) \right]^{-N/2}\left\| \boldsymbol{\Sigma} \right\|^{-1/2}exp\left[ -\frac{1}{2\hat{f_{l}}(1-\hat{f_{l}})}\left( \left( \mathbf{D}_{*l}\mathbf{-1}Y \right)^{\boldsymbol{T}}\boldsymbol{\Sigma}^{\mathbf{-1}}\left( \mathbf{D}_{*l}\mathbf{-1}Y \right)\mathbf{+}\mathbf{1}^{\mathbf{T}}\boldsymbol{\Sigma}^{\mathbf{-1}}\mathbf{1}\left( Y\mathbf{-}f_{l} \right)^{\mathbf{2}} \right) \right]$$

$$=\left[ 2\pi\hat{f_{l}}(1-\hat{f_{l}}) \right]^{-(N-1)/2}\left\| \boldsymbol{\Sigma} \right\|^{-1/2}\left[ \mathbf{1}^{\mathbf{T}}\boldsymbol{\Sigma}^{\mathbf{-1}}\mathbf{1} \right]^{\mathbf{-1/2}}exp\left[ -\frac{1}{2\hat{f_{l}}(1-\hat{f_{l}})}\left( \mathbf{D}_{*l}\mathbf{-1}Y \right)^{\boldsymbol{T}}\boldsymbol{\Sigma}^{\mathbf{-1}}\left( \mathbf{D}_{*l}\mathbf{-1}Y \right) \right]\times L\left( Y\mathbf{|}f_{l} \right)$$

where only the final term in the likelihood depends on $f_{l}.$

Intuitively, we view $Y$ as a weighted mean of the individual allele counts, specifically as an estimator of the ancestral allele frequency $f_{l}$. We have shown that $Y$ is a sufficient statistic for estimating $f_{l}.$ Further we can then write

$$L\left( \mathbf{D}_{\mathbf{*}\boldsymbol{l}}\mathbf{|}f_{l} \right)=L\left( \mathbf{D}_{\mathbf{*}\boldsymbol{l}}\mathbf{|}Y \right)L\left( Y\mathbf{|}f_{l} \right).$$

The ancestral allele frequency at an individual SNP is often not of direct interest in inferring structure, and is typically integrated out of the likelihood as a nuisance parameter. Therefore, suppose that we have placed some prior distribution $h$ on $f_{l}$. (For example, the original implementation of STRUCTURE uses a $U(0,1)$ prior for $h$.) We will also suppose that in most practical settings, this prior is relatively diffuse while the data are more informative, so that over the support of $L\left( Y\boldsymbol{|}f_{l} \right)$ we may view $h$ as unvarying: $h\left( f_{l} \right)\approx h(y)$. We now may approximately integrate out the ancestral allele frequency to give unconditionally:

$$L\left( \mathbf{D}_{*l} \right)=L\left( \mathbf{D}_{*l}\mathbf{|}Y=y \right)\int_{-\infty}^{\infty} L\left( y\mathbf{|}f_{l} \right)h\left( f_{l} \right)df_{l}\approx L\left( \mathbf{D}_{*l}\mathbf{|}Y=y \right)h\left( y \right)\int_{-\infty}^{\infty} L\left( y\mathbf{|}f_{l} \right)df_{l}$$

$$=L\left( \mathbf{D}_{*l}\mathbf{|}Y=y \right)h\left( y \right)$$

In our setting, we are interested in inferring structure. Thus, terms that concern us in the likelihood are only those that depend on this structure, which is wholly characterised by the covariance matrix $\boldsymbol{\Sigma}$ and so up to a constant of proportionality:

$$L\left( \mathbf{D}_{*l}\mathbf{|}f_{l} \right)\propto\left\| \boldsymbol{\Sigma} \right\|^{-1/2}\left[ \mathbf{1}^{\mathbf{T}}\boldsymbol{\Sigma}^{\mathbf{-1}}\mathbf{1} \right]^{\mathbf{-1/2}}exp\left[ -\frac{1}{2\hat{f_{l}}(1-\hat{f_{l}})}\left( \mathbf{D}_{*l}\mathbf{-1}Y \right)^{\boldsymbol{T}}\boldsymbol{\Sigma}^{\mathbf{-1}}\left( \mathbf{D}_{*l}\mathbf{-1}Y \right) \right].$$

In the large-sample size setting, this likelihood approximates the truth and contains almost all available information about population structure (ignoring information present in the overall average allele frequency $Y$ at a given SNP).

We must take only one more simplifying step, and that is to rewrite the above in terms of $\hat{f_{l}}$ rather than $Y$. Specifically, note that:

$$\left( \mathbf{D}_{*\boldsymbol{l}}-\mathbf{1}Y \right)^{\boldsymbol{T}}\boldsymbol{\Sigma}^{-\mathbf{1}}\left( \mathbf{D}_{*\boldsymbol{l}}-\mathbf{1}Y \right)=\left( \mathbf{D}_{*\boldsymbol{l}} \right)^{\boldsymbol{T}}\left( \text{I}-\mathbf{a}\text{1}^{\boldsymbol{T}} \right)\boldsymbol{\Sigma}^{-\mathbf{1}}\left( \text{I}-\mathbf{1}\text{a}^{\boldsymbol{T}} \right)\left( \mathbf{D}_{*\boldsymbol{l}} \right)$$

$$=\left( \mathbf{D}_{*\boldsymbol{l}} \right)^{\boldsymbol{T}}\left( \text{I}-\frac{\boldsymbol{\Sigma}^{-\mathbf{1}} \mathbf{1}\text{1}^{\boldsymbol{T}}}{\mathbf{1}^{\mathbf{T}}\boldsymbol{\Sigma}^{-\mathbf{1}} \mathbf{1}} \right)\boldsymbol{\Sigma}^{-\mathbf{1}}\left( \text{I}-\frac{{\mathbf{1}\text{1}}^{\boldsymbol{T}}\boldsymbol{\Sigma}^{-\mathbf{1}}}{\mathbf{1}^{\mathbf{T}}\boldsymbol{\Sigma}^{-\mathbf{1}} \mathbf{1}} \right)\left( \mathbf{D}_{*\boldsymbol{l}} \right)$$

$$=\left( \mathbf{D}_{*\boldsymbol{l}} \right)^{\boldsymbol{T}}\left( \boldsymbol{\Sigma}^{-\mathbf{1}}-\frac{\boldsymbol{\Sigma}^{-\mathbf{1}} \mathbf{1}\text{1}^{\boldsymbol{T}}\boldsymbol{\Sigma}^{-\mathbf{1}}}{\mathbf{1}^{\mathbf{T}}\boldsymbol{\Sigma}^{-\mathbf{1}} \mathbf{1}} \right)\left( \mathbf{D}_{*\boldsymbol{l}} \right)$$

$$=\left( \left[ \boldsymbol{I}-\frac{\mathbf{1}\text{1}^{\boldsymbol{T}}}{\mathbf{1}^{\mathbf{T}} \mathbf{1}} \right]\mathbf{D}_{*\boldsymbol{l}} \right)^{\boldsymbol{T}}\left( \boldsymbol{\Sigma}^{-\mathbf{1}}-\frac{\boldsymbol{\Sigma}^{-\mathbf{1}} \mathbf{1}\text{1}^{\boldsymbol{T}}\boldsymbol{\Sigma}^{-\mathbf{1}}}{\mathbf{1}^{\mathbf{T}}\boldsymbol{\Sigma}^{-\mathbf{1}} \mathbf{1}} \right)\left( \left[ \boldsymbol{I}-\frac{\mathbf{1}\text{1}^{\boldsymbol{T}}}{\mathbf{1}^{\mathbf{T}} \mathbf{1}} \right]\mathbf{D}_{*\boldsymbol{l}} \right)$$

$$=\left( \mathbf{D}_{*\boldsymbol{l}}-\hat{f_{l}}\mathbf{1} \right)^{\boldsymbol{T}}\left( \boldsymbol{\Sigma}^{-\mathbf{1}}-\frac{\boldsymbol{\Sigma}^{-\mathbf{1}} \mathbf{1}\text{1}^{\boldsymbol{T}}\boldsymbol{\Sigma}^{-\mathbf{1}}}{\mathbf{1}^{\mathbf{T}}\boldsymbol{\Sigma}^{-\mathbf{1}} \mathbf{1}} \right)\left( \mathbf{D}_{*\boldsymbol{l}}-\hat{f_{l}}\mathbf{1} \right)$$

where in the last line, $\hat{f_{l}}$ is simply the overall (unweighted) sample mean frequency. Finally, we have an approximate likelihood contribution for SNP $l$:

$$L\left( \mathbf{D}_{*l} \right)\propto\left\| \boldsymbol{\Sigma} \right\|^{-\frac{1}{2}}\left[ \mathbf{1}^{\mathbf{T}}\boldsymbol{\Sigma}^{-\mathbf{1}} \mathbf{1} \right]^{-\frac{\mathbf{1}}{\mathbf{2}}}exp\left[ -\frac{1}{2\hat{f_{l}}\left( 1-\hat{f_{l}} \right)}\left( \mathbf{D}_{*l}-\hat{f_{l}}\mathbf{1} \right)^{\boldsymbol{T}}\left( \boldsymbol{\Sigma}^{-\mathbf{1}}-\frac{\boldsymbol{\Sigma}^{-\mathbf{1}} \mathbf{1}\text{1}^{\boldsymbol{T}}\boldsymbol{\Sigma}^{-\mathbf{1}}}{\mathbf{1}^{\mathbf{T}}\boldsymbol{\Sigma}^{-\mathbf{1}} \mathbf{1}} \right)\left( \mathbf{D}_{*l}-\hat{f_{l}}\mathbf{1} \right) \right]. ∎$$

Given a set of drift parameters, and observed data $\mathbf{D}_{*l}$ for SNP $l,$ the likelihood can readily be calculated for inference purposes. However, the more useful feature of this approximation is that it makes it straightforward to combine information across loci, which we show leads to a strong link between certain forms of the principal components matrix, and model-based approaches using likelihoods of the general form discussed above.

### Linking the PCA/coancestry matrix and the model-based approach

Under our simplifying assumptions, we can now obtain a likelihood for the entire dataset by multiplying likelihoods across (unlinked) loci. The result is given by the following proposition, which easily extends to the genotype case.

Proposition 2

Suppose we have haploid variation data where sites are treated as unlinked (A1), that we sample $n_{1},$ $n_{2},\cdots,n_{K}$ individuals from each of $K$ underlying populations, where each $n_{k}$ is large, and that we exclude very rare SNPs (A2-3). Consider a general model of normally distributed allele frequency drift at each locus, with an individual level drift covariance matrix $\mathbf{H}$. If the prior placed on the ancestral allele frequency is weak (A8), and if we assume there is no information about drift from the overall allele frequency of each mutation in the sample (A6), then the likelihood of the data $\mathbf{D}$ can be approximated solely in terms of the “Eigenstrat” PCA matrix $\mathbf{E}$ from Proposition 1:

$$L\left( \mathbf{H |D} \right)\propto exp\left( \frac{L}{2}tr\left[ \log\left( \boldsymbol{\Sigma}^{\mathbf{-1}} \right) \right]-\frac{L}{2}\log\left( {\boldsymbol{(\Sigma}^{\mathbf{-1}}\mathbf{)}}_{..} \right)-\frac{L}{2}\left[ \sum_{i=1}^{N} \sum_{j=1}^{N} E_{ij}\left( \Sigma_{ij}^{-1}-\frac{\Sigma_{i\cdot}^{-1}\Sigma_{j\cdot}^{-1}}{\Sigma_{\cdot\cdot}^{-1}} \right)-\sum_{i=1}^{N} E_{ii} \right] \right)$$

where $\boldsymbol{\Sigma}=\mathbf{I}+\mathbf{H}.$

Hence in this model, *all* available information regarding the underlying population structure is contained in the PCA matrix.

#### Proof:

The stated assumptions mean that the result derived in Preliminary Proposition 2 holds. Hence, multiplying the likelihood across $L$ independent sites we obtain:

$$L\left( \mathbf{H |D} \right)\propto\left\| \boldsymbol{\Sigma} \right\|^{-\frac{L}{2}}\left[ {\boldsymbol{(\Sigma}^{\mathbf{-1}}\mathbf{)}}_{..} \right]^{-\frac{L}{2}}exp\left[ -\sum_{l=1}^{L} \frac{1}{2\hat{f_{l}}\left( 1-\hat{f_{l}} \right)}\left( \mathbf{D}_{\mathbf{*}\boldsymbol{l}}\mathbf{-}\hat{f_{l}}\mathbf{1} \right)^{\boldsymbol{T}}\left( \boldsymbol{\Sigma}^{\mathbf{-1}}\mathbf{-}\frac{\Sigma_{i\cdot}^{-1}\Sigma_{j\cdot}^{-1}}{\Sigma_{\cdot\cdot}^{-1}} \right)\left( \mathbf{D}_{\mathbf{*}\boldsymbol{l}}\mathbf{-}\hat{f_{l}}\mathbf{1} \right) \right]$$

$$\propto\left\| \boldsymbol{\Sigma} \right\|^{-\frac{L}{2}}\left[ {\boldsymbol{(\Sigma}^{\mathbf{-1}}\mathbf{)}}_{..} \right]^{-\frac{L}{2}}exp\left[ -\sum_{i=1}^{N} \sum_{j=1}^{N} \sum_{l=1}^{L} \frac{1}{2\hat{f_{l}}\left( 1-\hat{f_{l}} \right)}\left( D_{il}\mathbf{-}\hat{f_{l}} \right)\left( \boldsymbol{\Sigma}^{\mathbf{-1}}\mathbf{-}\frac{\Sigma_{i\cdot}^{-1}\Sigma_{j\cdot}^{-1}}{\Sigma_{\cdot\cdot}^{-1}} \right)_{ij}\left( D_{jl}\mathbf{-}\hat{f_{l}} \right) \right]$$

$$\propto\left\| \boldsymbol{\Sigma} \right\|^{-\frac{L}{2}}\left[ {\boldsymbol{(\Sigma}^{\mathbf{-1}}\mathbf{)}}_{..} \right]^{-\frac{L}{2}}exp\left( -\frac{L}{2}\left[ \sum_{i=1}^{N} \sum_{j=1}^{N} E_{ij}\left( \Sigma_{ij}^{-1}-\frac{\Sigma_{i\cdot}^{-1}\Sigma_{j\cdot}^{-1}}{\Sigma_{\cdot\cdot}^{-1}} \right)\mathbf{-}\sum_{i=1}^{N} E_{ii} \right] \right)$$

(noting that the last term in the exponent is a constant, so does not affect proportionality: this is included to avoid a large term in the exponent for $i=j$), from which the required result is immediate by properties of determinants.$∎$

This result is general for the study of datasets containing mainly common mutations. It applies in both models incorporating discrete population structure, and also in no-linkage admixture models, where sample size is in a sense “large”, in that the Normal approximation to the likelihood still applies. An implication is that apart from the weak information contained in the average allele frequencies of mutations across groups, and neglecting information from occasional variants that reach loss or fixation in some groups (expected to occur for stronger drift), the Eigenstrat style PCA matrix contains all the available information about population structure. Thus in these models, we expect model-based approaches to generally only succeed if there is a signal of structure from the PCA approach, as has been observed previously in practice (S5).

## S4.3: Asymptotic behaviour of models of population structure, with weak drift

If population structure is strong, we anticipate that all reasonable approaches are likely to identify it. Of more interest is then the setting where population structure is weak (in a sense we will specify precisely below). In this section, we show that in the setting of weak structure the likelihood of a particular underlying drift matrix $\mathbf{H}$ has a particularly simple approximate form. This likelihood is a function of our coancestry matrix. Further, we show that the likelihood form is approximately equivalent to that obtained by assuming that an appropriate rescaling of the coancestry matrix yields a multinomial distribution. Thus, in the weak drift case and for unlinked sites, the likelihood form we use in the main text (with an appropriate choice of scaling parameter) approximates the full likelihood of the data. (In the next section we will show how we generalise these ideas to the linked case, and describe our approach for estimating $c$.)

Proposition 3: Asymptotic behavior for subtle population structure

Assume that the conditions of Proposition 2 hold (A1-3,A6,A8), and also assumptions A4 and A5, where (A5) is precisely that the drift is small in the sense that order $\mathbf{H}^{3}$ and higher terms are negligible. The likelihood given by Proposition 2 can be simplified to

$$L\left( \mathbf{H |D} \right)\propto exp\left( -\frac{L}{4}\sum_{i=1}^{N} \sum_{j=1}^{N} \left[ Z_{ij}{\mathbf{-}Q}_{ij} \right]^{2} \right),$$

Where $\mathbf{Q}$ is the row and mean zero’d drift matrix with $Q_{ij}=H_{ij}\mathbf{-}\frac{1}{N}H_{i\cdot}-\frac{1}{N}H_{\cdot j}+\frac{1}{N^{2}}H_{\cdot\cdot}$, and $Z_{ij}=\frac{1}{L}\left( E_{ij}+\frac{L}{N} \right)$ is the per-locus PCA matrix.

#### Proof:

We will approximate terms in the likelihood shown in Proposition 2. Recalling $\boldsymbol{\Sigma}\mathbf{=}\mathbf{I}\mathbf{+}\mathbf{H}$, under assumption A5 we then have correct to second order:

$$\boldsymbol{\Sigma}^{\mathbf{-1}}\mathbf{=I-H+}\mathbf{H}^{\mathbf{2}}\boldsymbol{+ \cdots}$$

$$\Sigma_{\cdot\cdot}^{-1}=N-H_{\cdot\cdot}+H_{\cdot\cdot}^{2}\boldsymbol{+ \cdots}$$

$$\log\left( {\boldsymbol{(\Sigma}^{\mathbf{-1}}\mathbf{)}}_{..} \right)\mathbf{=}\log N-\frac{1}{N}H_{\cdot\cdot}+{\frac{1}{N}H}_{\cdot\cdot}^{2}-\frac{1}{2N^{2}}\left[ H_{\cdot\cdot} \right]^{2}\boldsymbol{+ \cdots}$$

$$\log\left( \boldsymbol{\Sigma}^{\mathbf{-1}} \right)\mathbf{=-H+}\frac{1}{2}\mathbf{H}^{\mathbf{2}}\boldsymbol{+ \cdots}$$

$$tr\left[ \log\left( \boldsymbol{\Sigma}^{\mathbf{-1}} \right) \right]\mathbf{=-}tr\left[ \mathbf{H} \right]\mathbf{+}\frac{1}{2}tr\left[ \mathbf{H}^{\mathbf{2}} \right]\boldsymbol{+ \cdots}$$

We start with the likelihood from Proposition 2:

$$L\left( \mathbf{H |D} \right)\propto exp\left( \frac{L}{2}tr\left[ \log\left( \boldsymbol{\Sigma}^{\mathbf{-1}} \right) \right]-\frac{L}{2}\log\left( {\boldsymbol{(\Sigma}^{\mathbf{-1}}\mathbf{)}}_{..} \right)-\frac{L}{2}\left[ \sum_{i=1}^{n} \sum_{j=1}^{n} E_{ij}\left( \Sigma_{ij}^{-1}-\frac{\Sigma_{i\cdot}^{-1}\Sigma_{j\cdot}^{-1}}{\Sigma_{\cdot\cdot}^{-1}} \right)\mathbf{-}\sum_{i=1}^{n} E_{ii} \right] \right)$$

Again correct to second order and after a little simplification we find:

$$\frac{L}{2}\left[ \sum_{i=1}^{N} \sum_{j=1}^{N} E_{ij}\left( \Sigma_{ij}^{-1}-\frac{\Sigma_{i\cdot}^{-1}\Sigma_{j\cdot}^{-1}}{\Sigma_{\cdot\cdot}^{-1}} \right)-\sum_{i=1}^{N} E_{ii} \right]\approx\frac{L}{2}\sum_{i=1}^{N} \sum_{j=1}^{N} E_{ij}\left( -H_{ij}+{H^{2}}_{ij}-\frac{1}{N}H_{i\cdot}H_{j\cdot} \right).$$

Note that viewing the PCA matrix as estimating the drift, and evaluating the expected asymptotic values for this matrix without drift as $N$ becomes large, we have $Z_{ij}=\frac{1}{L}\left( E_{ij}+\frac{L}{N} \right)=O(H_{ij})$ in large datasets, unless $i=j$. For the $i=j$ case write $\frac{1}{L}\left( E_{ii}+\frac{L}{N} \right)=1+Z_{ii}$. Again, for large datasets, $Z_{ii}=O(H_{ii})$ is small. Then, correct to second order in $H_{ij}$ we have:

$$\sum_{i=1}^{N} \sum_{j=1}^{N} E_{ij}\left( -H_{ij}+{H^{2}}_{ij}\mathbf{-}\frac{1}{N}H_{i\cdot}H_{j\cdot} \right)=\sum_{i=1}^{N} \sum_{j=1}^{N} \left( {LZ}_{ij}-\frac{L}{N} \right)\left( -H_{ij}+{H^{2}}_{ij}\mathbf{-}\frac{1}{N}H_{i\cdot}H_{j\cdot} \right)+L\sum_{i=1}^{N} \left( -H_{ii}+{H^{2}}_{ii}\mathbf{-}\frac{1}{N}H_{i\cdot}H_{i\cdot} \right)$$

$$\approx L\left[ \left( \sum_{i=1}^{N} \sum_{j=1}^{N} -Z_{ij}H_{ij} \right)+\frac{1}{N}H_{\cdot\cdot}+\frac{1}{N^{2}}\left[ H_{\cdot\cdot} \right]^{2}-\frac{1}{N}\left[ \mathbf{H}^{2} \right]_{\cdot\cdot}-tr\left[ \mathbf{H} \right]+tr\left[ \mathbf{H}^{2} \right]-{\frac{1}{N}\left[ H_{\cdot\cdot} \right]}^{2} \right]$$

Substituting into the likelihood:

$$L\left( \mathbf{H |D} \right)\propto exp\left( \frac{L}{2}\left( -tr\left[ \mathbf{H} \right]\mathbf{+}\frac{1}{2}tr\left[ \mathbf{H}^{\mathbf{2}} \right] \right)+\frac{L}{2}\left( \frac{1}{N}H_{\cdot\cdot}-\frac{1}{N}\left[ \mathbf{H}^{2} \right]_{\cdot\cdot}+\frac{1}{2N^{2}}\left[ H_{\cdot\cdot} \right]^{2} \right)-\frac{L}{2}\left[ \left( \sum_{i=1}^{N} \sum_{j=1}^{N} -Z_{ij}H_{ij} \right)+\frac{1}{N}H_{\cdot\cdot}+\frac{1}{N^{2}}\left[ H_{\cdot\cdot} \right]^{2}-\frac{1}{N}\left[ H^{2} \right]_{\cdot\cdot}-tr\left[ \mathbf{H} \right]+tr\left[ \mathbf{H}^{2} \right]-{\frac{1}{N}\left[ H_{\cdot\cdot} \right]}^{2} \right] \right)$$

and after simplification, we obtain

$$L\left( \mathbf{H |D} \right)\propto exp\left( -\frac{L}{2}\left( \frac{1}{2}tr\left[ \mathbf{H}^{\mathbf{2}} \right] \right)+\frac{L}{2}\frac{1}{N}\left[ \mathbf{H}^{2} \right]_{\cdot\cdot}-\frac{L}{2}\left( \frac{1}{2N^{2}}\left[ H_{\cdot\cdot} \right]^{2} \right)-\frac{L}{2}\left[ \sum_{i=1}^{N} \sum_{j=1}^{N} -Z_{ij}H_{ij} \right] \right)$$

$$L\left( \mathbf{H |D} \right)\propto exp\left( -\frac{L}{4}\left[ tr\left[ \mathbf{H}^{\mathbf{2}} \right]\mathbf{-}\frac{2}{N}\left[ \mathbf{H}^{2} \right]_{\cdot\cdot}+\frac{1}{N^{2}}\left[ H_{\cdot\cdot} \right]^{2}-2\sum_{i=1}^{N} \sum_{j=1}^{N} Z_{ij}H_{ij} \right] \right).$$

We now substitute $Q_{ij}=H_{ij}\mathbf{-}\frac{1}{N}H_{i\cdot}-\frac{1}{N}H_{\cdot j}+\frac{1}{N^{2}}H_{\cdot\cdot}$ , which clearly has zero row and column means, so $Q_{ij}$ is the relative drift among individuals. Further, by expansion of the desired result it is clear that all other terms from ${Q_{ij}}^{2}$ cancel, and ${Z_{ij}}^{2}$ is a constant independent of the parameters so can be included or excluded in the proportionality. The result immediately follows.$∎$

#### Discussion of Proposition 3:

This is the key result; to second order the likelihood only depends on the data through the usual principal components matrix $\mathbf{E}$ which is transformed to give $Z_{ij}$, and the transformed drift matrix $\mathbf{Q}$. Hence only the relative drift $\mathbf{Q}$ is identifiable from data, and the absolute overall value of drift cannot be inferred. Further, the likelihood behaves asymptotically as if the transformed entries $Z_{ij}$in the principal components matrix are independent and normally distributed, with mean $\mathbf{Q}$ and variance $\frac{2}{L}$.

We have used a series of approximations in deriving this result, which suppose essentially that the sample size is large, while drift is small. Examining the latter assumption in more detail, it can be seen that our approximations require the setting $\frac{H_{\cdot\cdot}}{N}\ll1$, so the average drift is small compared to $\frac{1}{N}$. By Price et al. (2009), overall structure is strong if

$$\frac{H_{\cdot\cdot}}{N^{2}}\gg\sqrt{\frac{1}{LN}}$$

In this setting, our assumptions may not hold but we expect (and see in practice) all competitive approaches to perform well and identify structure. In the case where structure is much weaker, then for some non-negative $k$ we can consider:

$$\frac{H_{\cdot\cdot}}{N}\sim k\sqrt{\frac{N}{L}}$$

which will be considerably less than 1 provided $N\ll L$, as is usually the case in current genetic studies where there are many more markers than individuals. Nevertheless, it is clearly important to evaluate the performance of both our approximation to the likelihood, and the resulting inference framework, via simulation, which we do extensively in Section S6. The results verify excellent agreement between the theory and observed results.

## S4.4: fineSTRUCTURE model

The likelihoods as written above produce a dimension reduction by avoiding the need to consider SNPs individually. However, this likelihood is not particularly convenient to work with directly – neither is it straightforward to extend to incorporate LD information. It is more natural to attempt to perform inference based on our coancestry matrices, which give expected counts, and *do* extend immediately to the LD case, while still giving a dimension reduction. We showed above that these matrices also relate closely to the PCA matrix, which our approximate likelihoods are defined in terms of.

For count data (and by extension our *expected* count data matrix), a natural model is the multinomial distribution, which is a member of the exponential family of distributions, enabling the use of computationally convenient conjugate prior choices. Although we have expected counts, we may nevertheless consider a model of the same form, where individuals are ordered. In general, we allow ourselves to multiply the count matrix by a constant $1/c$ before applying the likelihood. We can view this as calculating an *effective* number of loci across the genome.

In this section, we first attempt to identify a relationship between parameters in this multinomial likelihood, and the STRUCTURE model. The parameter in a multinomial model, for group $m$, is the probability an observation falls in this bin. Suppose we consider a general multinomial distribution for our setting, where we have a mean probability $P_{ij}$ that individual $i$ copies a SNP (or a chunk) from individual $j$. It is immediate from Proposition 1 that if $N$ is large, $i\neq j$, the expected count proportion for individual $i$ from individual $j$ is expressible in terms of the expectation of the PCA matrix and to order $N$ :

$$P_{ij}=E\left( \frac{1}{L}X_{ij} \right)$$

$$=\frac{1}{N-1}+\frac{1}{N-1}E\left[ {\frac{1}{L}E}_{ij} \right]$$

$$=\frac{1}{N}+\frac{1}{N-1}E\left[ Z_{ij} \right]$$

$$=\frac{1}{N}+\frac{1}{N-1}Q_{ij}\mathbf{.}$$

so we can relate this parameter to the individual by individual relative drift matrix $\mathbf{Q}$. Note we could conceptually also include the case $i=j$, even though we have disallowed self-copying in practice.

Proposition 4: Asymptotic behaviour of the multinomial likelihood

Assuming A1-6 and additionally (A7) that the whilst both the population size $N$ and number of loci $L$ are large, additionally $L\gg N$

$$L\left( \mathbf{X;P} \right)= \prod_{i=1}^{N} L\left( \mathbf{X}_{i}\mathbf{;}\mathbf{P}_{i} \right),$$

$$L\left( \mathbf{X}_{i}\mathbf{;}\mathbf{P}_{i} \right)\mathbf{=}\left[ \binom{L}{X_{i1},{\cdots,X}_{in}}\prod_{j=1,j\neq i}^{N} {P_{ij}}^{X_{ij}} \right]^{1/c}\mathbf{,}$$

has the same asymptotic form found in Proposition 3, with (for haploids) the choice $c=2/(N-2)$.

#### Proof and derivation:

The expected total number of chunks copied by individual $i$ from each other individual $j$ is large provided drift is small and the number of loci is large compared to the number of individuals.

We again employ the central limit theorem, this time relying on the fact that the number of *loci* is large. We approximate the joint distribution of the counts $\mathbf{X}_{i}$ using a multivariate normal distribution of dimension $N-1$ (because $X_{ii}=\mu_{ii}=0$ this entry is therefore removed from the likelihood). $\mathbf{X}_{i}$ is the count vector, $\boldsymbol{\mu}_{i}\boldsymbol{=}L\mathbf{P}_{i}$ is the expected number of counts, and $\boldsymbol{\Sigma}^{\mathbf{i}}$ is the model covariance matrix for row $i$ with the other rows $j$. Since $\boldsymbol{\Sigma}^{\mathbf{i}}\mathbf{=}diag({L\mathbf{P}}_{i})$, we can avoid having to use the generalised inverse directly, and have the standard likelihood form:

$$L\left( \mathbf{X;P} \right)\boldsymbol{\propto}\left( \prod_{i,j=1,j\neq i}^{N} {P_{ij}}^{-1/2c} \right)exp\left( \frac{-1}{2c}\sum_{i,j=1,j\neq i}^{N} \frac{\left( X_{ij}-LP_{ij} \right)^{2}}{{LP}_{ij}} \right)$$

$$=\left( \prod_{i,j=1,j\neq i}^{N} {P_{ij}}^{-1/2c} \right)exp\left( \frac{-L}{2c}\sum_{i,j=1,j\neq i}^{N} \frac{\left( \hat{P_{ij}}-P_{ij} \right)^{2}}{P_{ij}} \right)\mathbf{,}$$

where $\hat{P_{ij}}=X_{ij}/L$ is the empirical frequency of copying from individual $i$ from individual $j$. Then since $\sum_{j=1,j\neq i}^{N} P_{ij}=\sum_{j=1,j\neq i}^{N} \hat{P_{ij}}=1$,

$$exp\left( \frac{-L}{2c}\sum_{j=1,j\neq i}^{N} \frac{\left( \hat{P_{ij}}-P_{ij} \right)^{2}}{P_{ij}} \right)=exp\left( \frac{-L}{2c}\sum_{j=1,j\neq i}^{N} \frac{{\hat{P_{ij}}}^{2}}{P_{ij}}+\frac{L}{2c} \right).$$

Using Proposition 1, and noting that $E\left( E_{ij}/L \right)={O(N}^{-1})$:

$${\hat{P_{ij}}}^{2}\approx{(N-1)}^{-2}\left( \frac{E_{ij}}{L}+1 \right)^{2}={(N-1)}^{-2}\left( \frac{{2E}_{ij}}{L}+1+O(N^{-2}) \right)\approx{(N-1)}^{-2}\left( {2Z}_{ij}+1-2/N \right)$$

Substituting for $Q_{ij}$, and discarding lower order terms in $N$, we have:

$$L\left( \mathbf{X;P} \right)\dot{\boldsymbol{\propto}}\left( \prod_{i,j=1,j\neq i}^{N} {P_{ij}}^{-1/2c} \right)exp\left( \frac{-L}{2Nc}\sum_{i,j=1}^{N} \frac{1+2Z_{ij}-\frac{1}{N}}{1+Q_{ij}-\frac{1}{N}}+\frac{L^{2}}{2c} \right)$$

where $Z_{ij}$ is defined as above, and where in the final line we include an additional term in the exponent corresponding to $Z_{ii}$, assuming that the relative contribution of this single within individual term to the likelihood is small if $N$ is large.

The first term in this likelihood can be ignored for two reasons. Firstly, we can incorporate this term into the prior distribution on $\mathbf{P}_{i}$ since it does not depend on the data or the number of loci or individuals except for a constant of proportionality, for example by fitting the prior variance in copying fractions based on the data. Secondly, the contribution of this term is small. Substituting $P_{ij}=N^{-1}(1+N{{(N-1)}^{-1}Q}_{ij})$ we have:

$$\left( \prod_{i,j=1,j\neq i}^{N} {P_{ij}}^{-\frac{1}{2c}} \right)\mathbf{=}N^{\frac{N\left( N-1 \right)}{2c}}exp\left( \frac{-1}{2c}\sum_{i,j=1,j\neq i}^{N} log\left( 1+\left[ \frac{N}{N-1} \right]Q_{ij} \right) \right)$$

$$\mathbf{=}N^{\frac{N(N-1)}{2c}}exp\left( \frac{-1}{2c}\sum_{i,j=1,j\neq i}^{N} \left[ \left[ \frac{N}{N-1} \right]Q_{ij}-\frac{1}{2}\left[ \frac{N}{N-1} \right]^{2}{Q_{ij}}^{2}+\cdots\right] \right)\approx N^{\frac{N(N-1)}{2c}}exp\left( \frac{1}{4c}\left[ \frac{N}{N-1} \right]^{2}\sum_{i,j=1}^{N} {Q_{ij}}^{2} \right).$$

(Here we have discarded higher order terms in *N* and $\mathbf{Q}$, and noted that rows of $\mathbf{Q}$ sum to 0). The constant term can be incorporated into the proportionality, and the term inside the exponent is small compared to the remaining term in the likelihood. Choosing $c=\frac{2N}{\left( N-1 \right)^{2}}=\frac{2}{N-2}+O\left( N^{-3} \right)$, we see that for small drift ($L\gg N$) we can discard the above term, leading to:

$$L\left( \mathbf{X;P} \right)\boldsymbol{\propto} exp\left( \frac{-L}{4}\frac{\left( N-1 \right)^{2}}{N^{2}}\sum_{i,j=1}^{N} \frac{1+2Z_{ij}-\frac{1}{N}}{1+Q_{ij}-\frac{1}{N}} \right)$$

$$=exp\left( \frac{-L}{4}\frac{\left( N-1 \right)^{2}}{N^{2}}\left[ \sum_{i,j=1}^{N} \frac{1+2{\left[ \frac{N}{N-1} \right]Z}_{ij}}{1+\left[ \frac{N}{N-1} \right]Q_{ij}} \right] \right)$$

$$\approx exp\left( \frac{-L}{4}\frac{\left( N-1 \right)^{2}}{N^{2}}\left[ \sum_{i,j=1}^{N} \left( 1+2{\left[ \frac{N}{N-1} \right]Z}_{ij} \right)\left( 1-{\left[ \frac{N}{N-1} \right]Q}_{ij}+\left[ \frac{N}{N-1} \right]^{2}{Q_{ij}}^{2} \right) \right] \right)$$

$$\approx exp\left( \frac{-L}{4}\frac{\left( N-1 \right)^{2}}{N^{2}}\left[ N^{2}+\sum_{i,j=1}^{N} \left[ \frac{N}{N-1} \right]^{2}{Q_{ij}}^{2}-2\sum_{i,j=1}^{N} \left[ \frac{N}{N-1} \right]^{2}Q_{ij}Z_{ij} \right] \right).$$

On simplification and discarding higher than second order terms in the drift we have

$$L\left( \mathbf{X;P} \right)\propto exp\left( -\frac{L}{4}\sum_{i=1}^{N} \sum_{j=1}^{N} \left[ Z_{ij}{-Q}_{ij} \right]^{2} \right)$$

which precisely matches the form we derived in Proposition 3.$∎$

Thus by choosing $c=\frac{2}{N-2}$, our multinomial likelihood can be viewed as approximating the likelihood used by STRUCTURE run on the same data, at least in the case of a large number of unlinked loci, many individuals, and weak drift. Extension to the diploid case is trivial by substituting $N_{hap}=2N$ into $=\frac{2}{N_{hap}-2}$ , giving $c=\frac{1}{N-1}$ in this case.

#### Discussion of Proposition 4:

The above results motivate the extension that modelling the coancestry matrix as multinomial may be appropriate even in the case where linkage disequilibrium is present, particularly when the number of loci is large, provided an appropriate value is chosen for $c$. In the unlinked case, the value $c=\frac{2}{N-2}$ (for haploids) can be viewed as an adjustment for the fact that the variance of the entries in the count matrix is overestimated by the multinomial likelihood. Indeed, if structure is weak, the true counts variance is approximately a factor $N-2$ lower than that given in the multinomial model. Further, the symmetry of the matrix gives an additional factor 2 for the off-diagonal terms yielding $c=\frac{2}{N-2}$. Noting that the multinomial distribution is generally approximated by a multivariate normal, we suggest that in general the multinomial model may still give a reasonable approximation, provided we use a value of $c$ equal to twice the ratio of the correct underlying variance of the coancestry matrix to the “multinomial” estimated variance (at least if structure is “weak” – in the strong structure case results are insensitive to the value of $c$).

In the general case it is not possible to analytically identify $c$, so we instead estimate the true underlying variance of the number of chunks copied using a bootstrapping approach, calculate the required ratio using the genome-wide copying fractions, and substitute in this value of $c$ into the likelihood. The above theory demonstrates that this approach will work in the simple unlinked loci case at least. In Section S6 we demonstrate that the bootstrap approach leads to the same value of $c$ as the theory in the no-linkage case, and additionally works well in the linkage case using our copying model.
