# Supplementary material for: Inference of Population Structure using Dense Haplotype Data
Source: PLoS Genet. 2012 Jan 26;8(1):e1002453. doi: 10.1371/journal.pgen.1002453 (PMC3266881; doi:10.1371/journal.pgen.1002453)
Supplement: Text S5 — Simulation procedure for linked data using SFS_CODE. (PDF) [file pgen.1002453.s046.pdf]

TEXT S5 Simulation Procedure

Genetic recombination maps were produced as described by the International HapMap Consortium (2007). Each map corresponds to the following regions of the genome (in cM): 6.946, 12.265, 3.423, 8.391, 2.888, 2.140, 8.708, 3.323, 8.531 and 11.764. Each is a cumulative distribution function describing the relative rate of recombination in the 5Mb region, along with an overall recombination rate <rho>.

For each of the 10 genetic maps, we generated 20 regions of length 5Mb by running the program SFS_CODE (Hernandez 2008) 20 times with the command:

```
 sfscode 5 1 -Td 0 0.3133
-TS 0.087084 0 1 -TS 0.087084 0 2 -TS 0.094777 1 3 -TS 0.102469 2
4 -TE 0.110162 -Tg 0 26.861714 -N 5000 -n 100 -A -L 999 ... -l p 1 --rho F
<recmapfile> <rho>
```

where ... is 998 entries of 5003 followed by 5009. This is a trick to create a region of exactly 5Mb consisting of 999 linked regions at distance 1 from each other each of approximate length 5000 bases (the remaining bases are gaps). This split is required for efficient simulation. This generates 20 individuals from each of 5 populations with a split structure described in Figure 2A of the main text, using a model with exponential growth following a bottleneck; consult the SFS_CODE manual for details. This generates a sample of 100 individuals per population; the first n from each were sampled where a smaller number was required. The output of each of the 200 runs was converted to phase format using a script written in R (R Development Core Team 2009). We then used ChromoPainter to perform painting on the output of each region independently in order to get 200 coancestry matrices. Coancestry (i.e. chunk copy count) matrices are summed, and when less than 200 regions are used they are ordered to give an even contribution from the different genetic maps.

Note that although we do not use them here, chunk length and mutation count matrices are available. These can be combined across runs as follows: chunk length matrices are averaged with weights given by the number of counts, to give the average length of a chunk. Mutation matrices are averaged with weights given by (length matrix times count matrix) to obtain mutation rates (proportional to) per site. Our software includes 'ChromoCombine', a tool to combine multiple ChromoPainter files as described above which is helpful for parallelization of large (e.g. genomic) datasets.

References

Hernandez, R., 2008 A flexible forward simulator for populations subject to selection and demography. Bioinformatics **24:** 2786–2787.

International HapMap Consortium, 2007 A second generation human haplotype map of over 3.1 million SNPs. Nature **449:** 851–61.

R Development Core Team, 2009 R: A Language and Environment for Statistical Computing. ISBN 3-900051-07-0.
