# Supplementary material for: Inference of Population Structure using Dense Haplotype Data
Source: PLoS Genet. 2012 Jan 26;8(1):e1002453. doi: 10.1371/journal.pgen.1002453 (PMC3266881; doi:10.1371/journal.pgen.1002453)
Supplement: Text S6 — Empirical evaluation procedure for the scaling parameter . This includes the simulation procedure for unlinked data, and the empirical validation that our procedure correctly identifies . (PDF) [file pgen.1002453.s047.pdf]

TEXT S6 Empirical validation of c

TEXT S6.1 Calculation of c

We first segment the genome into contiguous segments of constant *number of chunks* d. The number of chunks donated to individual i from j in segment k is x_{ijk}, and d is chosen such that x_{ijk} is approximately independent (for different k and conditional on i and j). This means that different individuals may have a different number of segments if they have different patterns of recombination. In practice, we found that $d = 100$ works well for the HGDP data, but due to the high LD present in the linked simulation data, there were only an average of 20 chunks per region. We therefore took the whole region to be a segment in this case and computed c using the full 200 region dataset. Then we compute $s_{ij} = \sum_k(x_{ijk})$ and $s^2_{ij} = \sum_k(x^2_{ijk})$. If individual i has R_i segments in total, we can calculate the theoretical variance for x_{ij} by first estimating the rate of inheriting from each other individual $\hat{P}_{ij} = s_{ij}/\sum_j s_{ij}$ and substituting into the multinomial variance:

$$V_T(x_{ij}; P_{ij}) \approx V_T(x_{ij}; \hat{P}_{ij}) = \sum_j s_{ij}\hat{P}_{ij}(1 - \hat{P}_{ij})/R_i$$

The empirical variance is:

$$V_E(x_{ij}) = \frac{s^2_{ij}}{R_i - 1} - \frac{(s_{ij})^2}{R_i(R_i - 1)}$$

This leads (with correction for the known overcounting factor of 2) to the estimate of c:

$$c_{ij} = 2\frac{V_E(x_{ij})}{V_T(x_{ij}; \hat{P}_{ij})}$$

and we simply take the mean value as our estimate:

$$c = \frac{1}{N(N-1)} \sum_{i=1}^{N} \sum_{j=1 \neq i}^{N} c_{ij}$$

Note that we provide a helper program called 'ChromoCombine' that calculates this, and which can easily use the two options of d described. It also handles summation of multiple files in case of parallelization was used for processing individuals and/or chromosomes separately.

TEXT S6.2 Validation

In this section we present evidence of the effect of varying the rescaling factor 'c' on inference. Note that we view c as a summary of the data in the same way as

the coancestry matrix X, and not as a parameter - it is therefore not appropriate to perform inference for it in the standard Bayesian way.

For interpretation of the empirical evaluation presented, we note that when c is 'too large', the effective number of chunks is reduced and therefore any mistakes in population assignment will tend to be under-split, i.e. we will not distinguish efficiently between similar populations. When c is 'too small' our model believes it has more independent chunks than is true and therefore will tend to over-split populations. The smallest c that does not over-split is called efficient, and larger c are called conservative.

We start with the unlinked model in the case where there is no population structure. Provided population sizes are large, we expect the theoretical results derived in Section S4 above to hold. Specifically, the theoretical prediction (for the approximately correct data likelihood) in the case of unlinked data is (Proposition 4 of Section S4.4):

$$F(x|p) = \prod_{i=1,j=1}^{N} \left(\frac{P_{q_i q_j}}{\hat{n}_{q_j}} \right)^{x_{ij}(n-1)} \tag{1}$$

which is equal to Equation 1 of the main text:

$$F(x|p) = \prod_{i=1,j=1}^{N} \left(\frac{P_{q_i q_j}}{\hat{n}_{q_j}} \right)^{x_{ij}/c} \tag{2}$$

when $c = 1/(n-1)$.

For this section we have generated datasets containing 15000 non-rare ($> 5\%$ allele frequency) unlinked SNPs (at varying N) under the same splitting scenario as the main text. Simulation for each SNP was by a) generating the 'ancestral frequency' f with $p(f) \approx 1/f$ (since this is not a probability distribution, we first choose which of 20 bins in the range 0 and 1 the SNP is from, then sample f conditional on this), then b) applying a normally distributed drift matrix for population level drift Σ, giving population level frequency vector $\mathbf{g} \sim \text{MVN}(\mathbf{f}, f(1-f)\Sigma)$, and c) sampling individuals SNP values according to this frequency. (SNPs with empirical frequency below the 5% threshold were resampled). The covariance matrix for the drift was:

$$\Sigma = \begin{pmatrix} 0.02 & 0 & 0 & 0 & 0 \\ 0 & 0.02 & 0.015 & 0 & 0 \\ 0 & 0.015 & 0.02 & 0 & 0 \\ 0 & 0 & 0 & 0.02 & 0.01 \\ 0 & 0 & 0 & 0.01 & 0.02 \end{pmatrix}$$

The theoretical prediction is compared to our empirical estimate of c on this dataset in Figure S1 which shows that our theoretical understanding of c is correct,

i.e. that the correlation with the truth is 1 at the predicted value and that both the theoretical and empirical estimates of c are approximately efficient and equal for large N. The empirical estimate is conservative for small N.

Our empirical estimate of c is also applicable in the case of linked data, whether using our linked or unlinked models. For the linked simulated data described in the Results section of the main text, we perform a similar scan of c and N to check that our algorithm is computing an appropriate value of c in realistic circumstances. Figure S2 shows these results.

The value of c estimated by the empirical method again appears to be conservative for small N and approximately efficient for large N. In both cases, the 'truth' (if obtainable from the data) is still obtained for a very wide range of $c' > c$ i.e. greater than the estimated value. This demonstrates that exact specification of c is not an important issue for many practical purposes.

Note also that the value of c is significantly larger for linked data (in both the linkage and no-linkage models) than for the case of unlinked data. When the unlinked model is used, correlations between neighbouring loci due to linkage disequilibrium increase the variance between regions. When the linked model is used, c values do not fall substantially below 1, even for very large population sizes. Intuition behind the different behaviour of the linked and unlinked models comes from considering the uncertainty in chunk assignment. For the unlinked model, the number of haplotypes which a particular allele is identical to increases linearly with sample size. For the linked model, each addition individual in the sample has the chance of having a haplotype that is a still better match than any preceding haplotype. For this reason, the uncertainty of assignment of each haplotype does not change substantially as additional individuals are added.

We now describe a scenario in which neither the theoretical nor the empirical estimate of c work well; this is because there is not a single suitable value of c for which our model holds in this case. This is the case of unlinked markers with *strong* differentiation between populations, large numbers of markers and large sample sizes (Figure S4).Here the estimated value of c gives confident assignment of incorrect splits. The model predictions break down because individuals within the same population all share SNPs with individuals in other populations. If genetic drift is strong at individual SNPs, then sharing this coancestry can give inappropriately weighted information that the individuals are related to each other. In other words, the assumptions of Section S4, Propositions 3-4 do not hold.

It is important to note that this problem is less dramatic in linked data, and essentially does not arise in the linked model. To see why, we note that these correlations arise when an (unlinked) SNP is found in a individual that is common in *another* population but rare/absent otherwise. Such SNPs can only arise through strong drift, are not excluded because they are not rare overall, and are

interpreted as overly strong evidence of shared ancestry. As N becomes large with population sizes $n_a \propto N$, most SNPs provide $O(N^{-1})$ information on population level copying proportions (which is why $c = O(N^{-1})$). However, strongly drifted SNPs provide $O(1)$ evidence because they are shared with a high fraction of another population, and not with any other individual. For (truly) linked data, such a SNP will be down-weighted due to the average level of correlation between nearby SNPs, so even in the unlinked model we will infer a larger value of c. For the linked model, all chunks are approximately unique and therefore provide $O(1)$ information per chunk, so a strongly drifted SNP will not have a dramatic influence since all *chunks* are already 'strongly drifted'. The success of our algorithm for simulated linked data also supports this argument.

For the strong drift and unlinked case, we have developed an alternative algorithm in which the likelihood is modified so that these correlations are accounted for. We do this in effect by considering only within-population counts as important, so that when considering a merge move, the between-population counts are normalised to have the same mean. We re-normalise using:

$$x'_{ij} = x_{ij} - \frac{\sum_{k \in q_i} x_{kj}}{|q_i|} - \frac{\sum_{l \in q_j} x_{il}}{|q_j|} + 2\frac{\sum_{k \in q_i} \sum_{l \in q_j} x_{kj}}{|q_i||q_j|} \tag{3}$$

where q_i is the index of the population for individual i, and $|q_i|$ is the number of individuals in that population. This corresponds to ensuring that all row and column sums copying from population b to population a are equal. This is illustrated in Figure S3 which shows the coancestry heatmap for the unnormalized and normalised cases, as well as the difference between them. The coancestry heatmaps are visually very similar, but the undesired correlation structure is clearly visible in this difference. Some individuals have an elevated number of donated chunks to all individuals *within a specific population*, leading to a 'striped' pattern. The bottom plots show the same thing but where we consider a potential merged population (merging the most recent split). It is clear that the presence of population structure is preserved under this procedure because the two populations have a different profile *within the population being merged.* Therefore the standard likelihood applied to both the merged and split matrices can correctly identify both populations due to their different rates of copying within and between, and additionally it is not mislead by the correlated copying from other populations. However, were the only distinction between populations B1 and B2 the copy rate from some third population, this would be normalised out.

Note that our simple likelihood is not a well defined entity under this modification, because the data depends on the population assignment. There is however an implicit likelihood induced by the modification of the data which is well defined and is correctly comparable both within states of a given number of populations K and for states of differing K, provided that we consider moving an individual

between two populations as creating a merged state between the two populations (which defines the normalisation), and creating a split state corresponding to the move.

Although this procedure is more robust than the use of the raw coancestry matrix, it is not recommended for general use because firstly, it discards information about a split that comes from differential chunk counts from other populations, and secondly, it is not a clearly defined model. We have tested this procedure on the HGDP data (unlinked model, results not shown) and obtain broadly similar results to those quoted in the main text with some subtle splits lost: for example, the Tuscan/Italian split is not fully supported. We recommend using it as a conservative check if the value of c is very low (say less than 0.05) and there is strong structure in the dataset.
