# Supplementary material for: Inference of Population Structure using Dense Haplotype Data
Source: PLoS Genet. 2012 Jan 26;8(1):e1002453. doi: 10.1371/journal.pgen.1002453 (PMC3266881; doi:10.1371/journal.pgen.1002453)
Supplement: Text S7 — Empirical comparison of fineSTRUCTURE to STRUCTURE. (PDF) [file pgen.1002453.s048.pdf]

TEXT S7 Comparison to STRUCTURE

We have shown that in theory, and in the unlinked model case, STRUCTURE and fineSTRUCTURE are using approximately the same data and the same model, under certain limiting conditions. It is important to assess how these conditions apply in practice. Figure S5 shows the correlation with the truth, as the number of SNPs changes, for both fineSTRUCTURE and STRUCTURE for N=100 individuals sampled from the same population structure as described in the main text for the unlinked case. From this figure two things are evident. Firstly, at low SNP numbers, STRUCTURE outperforms fineSTRUCTURE by a small margin. However, as the number of SNPs increases, STRUCTURE does not keep improving its performance due to two effects. Firstly, it becomes very difficult to mix the SNP frequencies with the other parameters, and so the MCMC sampling becomes poor. We can see this by starting STRUCTURE both at the truth and from random starting locations; for large numbers of SNPs it fails to find even an adequate K=3 solution (we here show the best solution found in several runs). Secondly, the prior (F-model) STRUCTURE uses assumes independent drift for all populations, and scales with the number of SNPs. Therefore the correlated drift observed in this population scenario looks equally unlikely in the model regardless of the number of SNPs, and even when started at the truth STRUCTURE favours lower values of K. Although fineSTRUCTURE also does not have explicit correlated drift in the prior, the prior does not scale with the number of SNPs and therefore the data can overwhelm any prior structure placed on the coancestry matrix. This leads to slightly conservative splitting at all scales, as we must have positive evidence of a split, hence the very abrupt change from a K=3 to a K=4 solution (and similarly for K=5).

From the theory, we would expect that as the number of individuals increases, fineSTRUCTURE tends towards the STRUCTURE performance at lower SNP counts. The message from this comparison is that the loss of information in performing the summary step is not high for datasets with hundreds of markers, but that if few, genuinely unlinked markers are used, the STRUCTURE model is preferable. For larger numbers of markers, fineSTRUCTURE is to be preferred even if the markers are unlinked.
