# Supplementary material for: Inference of Population Structure using Dense Haplotype Data
Source: PLoS Genet. 2012 Jan 26;8(1):e1002453. doi: 10.1371/journal.pgen.1002453 (PMC3266881; doi:10.1371/journal.pgen.1002453)
Supplement: Text S8 — Details of the ADMIXTURE linked simulation evaluation procedure. (PDF) [file pgen.1002453.s049.pdf]

TEXT S8 ADMIXTURE linked simulations analysis

For the linked simulations we have compared our results with the program ADMIXTURE (Alexander, Novembre, and Lange 2009). ADMIXTURE computes the same likelihood as STRUCTURE but performs maximum-likelihood analysis, i.e. it does not perform MCMC sampling and does not apply a prior. This makes is significantly faster and avoids many mixing problems, and is easily applicable to the HGDP dataset. To perform this analysis, we took the same phased haplotype data used as input for ChromoPainter and converted it to PLINK format (Purcell, Neale, Todd-Brown, Thomas, Ferreira, Bender, Maller, Sklar, de Bakker, Daly, and Sham 2007) (PLINK version 1.07, downloaded from `http://pngu.mgh.harvard.edu/~purcell/plink/`) using which we extracted the SNPs with minor frequency > 0.01. The filesizes were too large for manipulation within PLINK with 200 regions and therefore we used a minor frequency cutoff of 0.02 in this case. We then ran ADMIXTURE for various numbers of populations K and for varying number of regions.

We here show the details of the ADMIXTURE results since direct comparison between the methods is not possible, fineSTRUCTURE being an MCMC based no-admixture model and ADMIXTURE reporting only maximum-likelihood admixture results. The correlation reported in the paper is created by forcing the admixture solution to choose the most likely population for each individual; however, performing the correlation on the admixed solution does not change the results qualitively.

References

Alexander, D. H., J. Novembre, and K. Lange, 2009 Fast model-based estimation of ancestry in unrelated individuals. Genome Research **19:** 1655–1664.

Purcell, S., B. Neale, K. Todd-Brown, L. Thomas, M. Ferreira, D. Bender, J. Maller, P. Sklar, P. de Bakker, M. Daly, and P. Sham, 2007 PLINK: a toolset for whole-genome association and population-based linkage analysis. American Journal of Human Genetics **81:** 559–75.
