# Supplementary material for: Inference of Population Structure using Dense Haplotype Data
Source: PLoS Genet. 2012 Jan 26;8(1):e1002453. doi: 10.1371/journal.pgen.1002453 (PMC3266881; doi:10.1371/journal.pgen.1002453)
Supplement: Text S9 — Details of the ADMIXTURE HGDP analysis. (PDF) [file pgen.1002453.s050.pdf]

TEXT S9 ADMIXTURE HGDP Europe analysis

For the European HGDP dataset we have compared our results with the program ADMIXTURE (Alexander, Novembre, and Lange 2009). ADMIXTURE computes the same likelihood as STRUCTURE but performs maximum-likelihood analysis, i.e. it does not perform MCMC sampling and does not apply a prior. This makes it significantly faster and avoids many mixing problems, and is easily applicable to the HGDP dataset. To perform this analysis, we took the same phased haplotype data used as input for ChromoPainter and converted it to PLINK format (Purcell, Neale, Todd-Brown, Thomas, Ferreira, Bender, Maller, Sklar, de Bakker, Daly, and Sham 2007) (PLINK version 1.07, downloaded from `http://pngu.mgh.harvard.edu/~purcell/plink/`) using which we extracted the SNPs variable in Europe with minor frequency > 0.01 and merged the chromosome 1–22 data leaving 585420 SNPs. We then ran ADMIXTURE for various numbers of populations K.

The results of this analysis are shown in Figure S12. The ADMIXTURE analysis requires that the user specify the value of K. In the standard (Bayesian) STRUCTURE approach, this can be estimated by computing the marginal probability of the data given the model with a particular K. The Bayesian approach with this model is however not feasible in the HGDP dataset. There is no robust way to compute the marginal probability in the maximum-likelihood setting and therefore ADMIXTURE instead tries to minimise the 'cross-validation error', that is, the error in predicting the value of a SNP under a cross validation scheme. In general this should be high both when the model is too simple or when it is overfitted. However, the HGDP dataset has a large number of uninformative SNPs and as Figure S13 shows the cross-validation error is minimised at $K = 1$. This occurs in part because the between-population variance is small compared to the within-population variance and hence adding population structure doesn't aid prediction. We are interested in explanatory power rather than prediction, and therefore have decided in the paper to show $K = 7$ which seems to capture many of the features in our fineSTRUCTURE analysis without obvious overfitting. We expect that the cross-validation error varies across SNPs and that higher K could be obtained by appropriate restriction to only informative SNPs.

As a check, we also used PLINK to perform linkage-based trimming of SNPs as suggested in the ADMIXTURE manual; this left a dataset of 121613 SNPs and provided broadly the same results (not shown). This shows that linkage disequilibrium has not dramatically distorted the analysis. We have discussed the results in the main text; we note here that the results as K is increased demonstrate an interesting pattern of successive population identification that correlate with our identification of populations that have drifted since admixture.

References

Alexander, D. H., J. Novembre, and K. Lange, 2009 Fast model-based estimation of ancestry in unrelated individuals. Genome Research **19:** 1655–1664.

Purcell, S., B. Neale, K. Todd-Brown, L. Thomas, M. Ferreira, D. Bender, J. Maller, P. Sklar, P. de Bakker, M. Daly, and P. Sham, 2007 PLINK: a toolset for whole-genome association and population-based linkage analysis. American Journal of Human Genetics **81:** 559–75.
