# Supplementary material for: Inference of Population Structure using Dense Haplotype Data
Source: PLoS Genet. 2012 Jan 26;8(1):e1002453. doi: 10.1371/journal.pgen.1002453 (PMC3266881; doi:10.1371/journal.pgen.1002453)
Supplement: Text S10 — Results for HGDP data. These comments interpret Figures S14, S15, S16, S17, S18, S19, S20, S21, S22, S23, S24, S25, S26, S27, S28, S29, S30, S31, S32, S33, S34, S35, S36, S37, S38, S39, S40, i.e. the fineSTRUCTURE and PCA-based continent and sub-continent analyses for the HGDP dataset. (PDF) [file pgen.1002453.s051.pdf]

TEXT S10 Results for HGDP data

The full coancestry matrix for the world is given in Figure S14. Note that the colour scale is non-linear and that small changes produce a large colour change at the lower end of the scale; this does however correspond roughly to meaningful changes since these colours are present at higher frequency in the matrix, and therefore small variation at this scale may be picked up by the model as it can involve many individuals.

Continents are defined according to Table 1. For the sub-continental analysis, Figure S15 shows the tree formed by forming a tree from each sub-continent and then the super-tree formed by continuing the tree-building process from these clusters. The coancestry matrix at the sub-continent level is shown in Figure S16 and throughout, sub-continents are defined in Figure 4 of the main text. Note that the same groupings appear in Figure S15 as Figure 4, but at a different height due to a different number of sub-populations found in each and therefore cannot be 'cut' at a single height. Figures S17 – S24 show the coancestry matrices for each of the sub-continents individually.

Figures S25 – S33 show convergence results (i.e. the pairwise coincidence matrices of two independent runs) for each HGDP dataset that we consider, i.e. the full dataset and the sub-continents.

Principal Components Analysis (Figures S24 – S40) are shown only for *continents*, as defined in Table 1 based on labels, and not the sub-continents. We omitted the whole-world PCA analysis because it is dominated by fairly trivial between-continent variation and shows a complex picture for structure within-continents at higher components. The sub-continental analysis broadly resembles the continental analysis but is less clear when labels cross sub-continental boundaries.
